# A Phase II Trial of the WEE1 Inhibitor Adavosertib in *SETD2-*Altered Advanced Solid Tumor Malignancies (NCI 10170)

**DOI:** 10.1158/2767-9764.CRC-24-0213

**Published:** 2024-07-23

**Authors:** Edward Maldonado, W. Kimryn Rathmell, Geoffrey I. Shapiro, Naoko Takebe, Jordi Rodon, Devalingam Mahalingam, Nikolaos A. Trikalinos, Arash R. Kalebasty, Mamta Parikh, Scott A. Boerner, Celene Balido, Gregor Krings, Timothy F. Burns, Emily K. Bergsland, Pamela N. Munster, Alan Ashworth, Patricia LoRusso, Rahul R. Aggarwal

**Affiliations:** 1 University of California, San Francisco, San Francisco, California.; 2 Vanderbilt University Medical Center, Nashville, Tennessee.; 3 Dana-Farber Cancer Institute, Boston, Massachusetts.; 4 National Cancer Institute, Bethesda, Maryland.; 5 Investigational Cancer Therapeutics, MD Anderson Cancer Center, Houston, Texas.; 6 Northwestern University, Evanston, Illinois.; 7 Washington University St. Louis, St. Louis, Missouri.; 8 University of California, Irvine, Irvine, California.; 9 University of California, Davis, Davis, California.; 10 Yale University, New Haven, Connecticut.; 11 University of Pittsburgh Medical Center, Pittsburgh, Pennsylvania.

## Abstract

We sought to evaluate the efficacy of WEE1 inhibitor adavosertib in patients with solid tumor malignancies (cohort A) and clear cell renal cell carcinoma (ccRCC; cohort B). NCT03284385 was a parallel cohort, Simon two-stage, phase II study of adavosertib (300 mg QDAY by mouth on days 1–5 and 8–12 of each 21-day cycle) in patients with solid tumor malignancies harboring a pathogenic *SETD2* mutation. The primary endpoint was the objective response rate. Correlative assays evaluated the loss of H3K36me3 by IHC, a downstream consequence of *SETD2* loss, in archival tumor tissue. Eighteen patients were enrolled (9/cohort). The median age was 60 years (range 45–74). The median duration of treatment was 1.28 months (range 0–24+). No objective responses were observed in either cohort; accrual was halted following stage 1. Minor tumor regressions were observed in 4/18 (22%) evaluable patients. Stable disease (SD) was the best overall response in 10/18 (56%) patients, including three patients with SD > 4 months. One patient with ccRCC remains on treatment for >24 months. The most common adverse events of any grade were nausea (59%), anemia (41%), diarrhea (41%), and neutropenia (41%). Nine patients (50%) experienced a Grade ≥3 adverse event. Of eight evaluable archival tissue samples, six (75%) had a loss of H3K36me3 by IHC. Adavosertib failed to exhibit objective responses in *SETD2*-altered ccRCC and other solid tumor malignancies although prolonged SD was observed in a subset of patients. Combination approaches may yield greater depth of tumor response.

**Significance:**

WEE1 inhibition with adavosertib monotherapy demonstrated limited clinical activity in patients with *SETD2*-altered solid tumors despite compelling preclinical data indicating a synthetic lethal effect, which did not translate into robust tumor regression. Loss of the H3K36me3 trimethylation mark caused by *SETD2*-deficiency was confirmed in the majority of evaluable tumors. A subset of patients derived clinical benefit as manifested by minor tumor regressions and prolonged SD.

## Introduction

SET domain containing 2 (*SETD2*) is a common tumor suppressor gene that encodes for a histone H3 lysine 36 (H3K36) methyltransferase. Pathogenic loss-of-function mutations have been observed across a wide range of solid tumor malignancies including clear cell renal cell carcinoma (ccRCC; range 3%–14%, though has been reported as high as 35% in some studies; refs. 1–6). *SETD2* mutations may not necessarily be biallelic. In ccRCC, the vast majority of tumors undergo chromosome 3p deletion in a region known to harbor several genes including *VHL*, *SETD2*, *PBRM1*, and *BAP1* (4, 6). Although most ccRCC tumors are monoallelic for these 3p genes, a smaller portion of ccRCC tumors acquire a second loss of function *SETD2* mutation in the remaining allele rendering the loss biallelic with complete loss of H3K36 methyltransferase.

*SETD2* has a canonical function as a key methyltransferase responsible for trimethylation of histone H3K36 (H3K36me3), a mark which plays a role in transcription, splicing, DNA damage repair, and maintenance of genomic integrity and stability (7). *SETD2* also has methyltransferase activity toward alternative targets such as α-tubulin and interacts with several other proteins including TP53 (3, 8, 9). Haploinsufficiency for *SETD2* causes a subset of the genomic instability seen with biallelic loss (10). Clinically in ccRCC, biallelic *SETD2* alterations have been associated with adverse cancer-specific outcomes such as higher tumor stage, increased likelihood for recurrence or metastatic disease, and worse cancer-specific survival (11, 12). There have also been reports of worse outcomes for other *SETD2-*deficient tumors including breast (3, 13) The widespread loss of *SETD2* in various malignancies and association with poor prognosis warrants therapeutic development of agents targeting this alteration.

WEE1 is an inhibitory tyrosine kinase, interacting with CDK1 and CDK2 at various points in the cell cycle including the S-phase and G2/M-phase transition, resulting in S-phase and G2/M-phase transition delays and the suppression of early mitotic events and mitotic catastrophe (14). Inhibition of WEE1 was previously found to promote unscheduled mitotic entry through CDK1 activation, leading to loss of genome integrity (15). Additionally, preclinical studies of WEE1 inhibition with adavosertib (also known as AZD1775) have demonstrated a synthetic lethal effect in *SETD2*-deficient cancers in cell lines (16). *SETD2* deficiency results in reduced trimethylation of histone H3K36 (H3K35me3), leading to reduced expression of ribonucleotide reductase regulatory subunit M2 (RRM2), which leads to further reduction in dNTP subunits. Therefore, resulting in effects on transcription, splicing, DNA damage repair, and maintenance of genomic integrity and stability (16) With the addition of WEE1 inhibition, preclinical studies showed the abolishment of DNA replication in *SETD2*-deficient cells through further depletion of dNTP pools (monomeric units of DNA) via further reduced expression of RRM2 (16). Adavosertib monotherapy demonstrated both synthetic lethal effects and resulted in significant tumor regression in *SETD2*-deficient renal cell carcinoma xenograft models (16).

Adavosertib (AZD1775) is a potent and selective WEE1 inhibitor that was previously evaluated in a phase I trial investigating doses ranging from 200 to 400 mg once daily (days 1–5 and 8–12 of each 21-day cycle) in 42 patients with advanced solid tumors (17). There were two dose-limiting toxicities observed at the 400 mg daily dosing level (grade 4 pancytopenia), thereby establishing 300 mg once daily (days 1–5 and 8–12 of each 21-day cycle) as the RP2D with similar plasma exposures to those from twice-daily dosing, with partial responses in 6/42 (14%) of patients (17). Thus, this RP2D was also used for this subsequent phase 2 study.

The purpose of this phase II study (NCT03284385) was to evaluate the efficacy and safety of adavosertib monotherapy in patients with *SETD2-*altered ccRCC and other locally advanced or metastatic solid tumor malignancies.

## Materials and Methods

### Study design and participants

This was a Simon two-stage, phase II, parallel cohort study that evaluated adavosertib monotherapy in biologically male and female patients at least age 18 years and older, with histologically confirmed locally advanced or metastatic solid tumor malignancies in two cohorts: (A) Solid tumor malignancies other than ccRCC and (B) ccRCC. Patients were not randomized and were not blinded in this phase 2 study. Cohort A (*N* = 9) included patients with solid tumor malignancies other than ccRCC with disease progression on at least one prior systemic therapy. Cohort B (*N* = 9) included patients with ccRCC with disease progression on at least one prior systemic therapy including prior tyrosine kinase inhibitor or immune checkpoint inhibitor. All patients were required to have evidence of a pathogenic *SETD2* mutation in archival tumor tissue by a local or central Clinical Laboratory Improvement Amendments–approved assay. Molecular profiling platforms included the following: Caris Comprehensive Molecular Profiling (2/18 patients; ref. 18), FoundationOne CDx (8/18 patients; ref. 19), Tempus Xt (4/18 patients; ref. 20), the Massachusetts General Brigham Dana–Farber Cancer Institute OncoPanel (3/18 patients; ref. 21), and the MD Anderson Solid Tumor Genomic Assay Tumor DNA Panel (1/18 patients; ref. 22). Additional eligibility criteria included measurable disease by RECIST 1.1 criteria, an Eastern Cooperative Oncology Group performance status of 0 to 1, and adequate hematologic, renal, and hepatic function. A full list of exclusion criteria is provided in Supplementary Protocol S1, which provides the study protocol.

The study was conducted in accordance with the International Conference on Harmonisation Guidelines for Good Clinical Practice and the principles of the Declaration of Helsinki. All patients provided written informed consent. This study (NCT03284385) was conducted at 11 different cancer centers in the United States through the National Cancer Institute (NCI) Experimental Therapeutics Clinical Trials Network under an NCI-sponsored investigational new drug application.

### Study treatments and procedures

Patients received adavosertib at a starting dose of 300 mg once daily by mouth on days 1 to 5 and 8 to 12 of each 21-day cycle. Dose modifications were allowed based on established criteria without the need for weight-based dosing (see Supplementary Protocol S1). Intra-patient dose re-escalation was not permitted. Treatment with adavosertib continued until disease progression by RECIST 1.1 criteria, unacceptable adverse events, clinical progression, and general or specific changes in the condition of the patient that rendered the patient unacceptable for further treatment as judged by the investigator.

Safety assessments were performed at regular intervals per the protocol. These included routine vital signs, physical exams, safety labs (complete blood count with differential; complete metabolic panel, LDH), and adverse event evaluations on Cycle (C) 1 Day (D) 1, C1D8, C1D15, C2D1, C2D8, and subsequently at the beginning of each cycle from C3D1 onward. ECGs were obtained at baseline and at the beginning of each cycle from C1D1 onward. Radiologic evaluation with tumor measurements was obtained at baseline and every 9 weeks ± 1 week.

For correlative analysis, formalin-fixed, paraffin-embedded tissue was obtained from archival tumor specimens during the screening process for enrolled patients when available. IHC was performed on formalin-fixed, paraffin-embedded tissue sections in the UCSF Cancer Center Tissue Core using a rabbit polyclonal antibody against H3K36me3 (Cell Signaling Technology, #9763). All immunostains were scored by a board-certified anatomic pathologist (GK). Negative H3K36me3 expression was defined as no nuclear staining in the tumor cells. Positive H3K36me3 expression was quantitated as a percentage of tumor cell nuclei stained per total tumor cell nuclei and scored as weak (1+), moderate (2+), or strong (3+) intensity. Background stromal and inflammatory cells were used as internal controls (positive nuclear staining). External controls included tumors confirmed to harbor inactivating *SETD2* mutations (H3K36me3-negative), tumors confirmed to lack *SETD2* mutations (H3K36me3-positive), and nonneoplastic normal liver tissue.

## Outcomes

The primary endpoint was the investigator-assessed objective response rate (ORR) by RECIST 1.1. Patients who received study treatment and at least one imaging assessment on study after C1D1 were considered evaluable for the primary endpoint. Secondary endpoints include progression-free survival (PFS), duration of response, and frequency and severity of adverse events. PFS was defined as the duration from the start of treatment to the date of progression by RECIST criteria. Safety analyses were obtained for all patients who received at least one dose of adavosertib, by patient summary as graded by the NCI Common Terminology Criteria for Adverse Events Version 5.0. Exploratory analyses included evaluation of the loss of trimethylation H3K36me3 mark by IHC from archival tumor tissue along with specific types of *SETD2* alteration and their association with clinical outcomes.

### Statistical analysis

The study employed a Simon two-stage design for each cohort. Nine patients were accrued in the first stage of the study in each of the two cohorts. In either cohort, if one or more confirmed objective responses were observed, an additional 21 patients were planned to be accrued during stage 2 in that particular patient cohort. If more than four confirmed objective responses were observed in total, the null hypothesis was rejected. The target total sample size achieved 90% power to detect a difference in objective response of 20% [25% vs. 5% historical control from prior phase 1 studies (18, 19)] with a one-sided type I error rate of 5%. The cutoff date for efficacy analysis was November 4, 2023 (one patient in Cohort B remained on treatment at that time). All 18 evaluable patients who received at least one dose of adavosertib were included in the safety population.

### Data availability

The data generated in this study are not publicly available because of patient privacy requirements but are available upon reasonable request from the corresponding author. Other data generated in this study are available within the article and its supplementary data files.

## Results

### Patient characteristics

Between May 2019 and October 2021, 18 patients were enrolled in the intention-to-treat population. Baseline characteristics are shown in Table 1. The median age at study entry was 60 years (range 45–74), Eastern Cooperative Oncology Group PS 0 in 8/18 patients (44.4%). In Cohort A (other solid tumors), 44.4% received prior immunotherapy, 44.4% received prior tyrosine kinase inhibitors, and 77.8% received “other” chemotherapy. In Cohort B (ccRCC), 88.9% of patients received prior immunotherapy, 66.7% received prior tyrosine kinase inhibitors, and 11.1% received “other” chemotherapy. In both cohorts, the median number of prior systemic therapies was 2.

**Table 1:** Baseline characteristics, *SETD2* mutations, co-occurring mutations, sites of metastases, and prior therapies

Characteristic	Total
Age, median (range), years	62 (45–74)
Biological sex, *N* (%)	
Male	11 (61.1%)
Female	7 (38.9%)
Race, *N* (%)	
White	16 (88.9%)
American Indian/Alaska Native	1 (5.6%)
Unknown	1 (5.6%)
Ethnic origin, *N* (%)	
Non-Hispanic	8 (44.4%)
Unknown	10 (55.6%)

	Cohort A: Solid tumors other than ccRCC (*N* = 9)	Cohort B: ccRCC (*N* = 9)	Total (*N* = 18)
Other solid tumor types, *N* (%)			
Poorly differentiated carcinoma	2 (22.2%)		
Salivary gland carcinoma	2 (22.2%)		
Rectal adenocarcinoma	1 (11.1%)		
Neuroendocrine carcinoma	1 (11.1%)		
Pancreatic adenocarcinoma	1 (11.1%)		
Lung adenocarcinoma	1 (11.1%)		
Papillary (nonclear cell) kidney cancer	1 (11.1%)		
*SETD2* alteration type, N (%)			
Missense	1 (11.1%)	0 (0%)	1 (5.6%)
Nonsense	3 (33.3%)	4 (44.4%)	7 (38.9%)
Frameshift	5 (55.5%)	4 (44.4%)	9 (50.0%)
Splice site	0 (0%)	1 (11.1%)	1 (5.6%)
Co-occurring genomic alterations, *N* (%)			
*TP53*	2 (22.2%)	1 (11.1%)	3 (16.7%)
*CDKN2A/B* loss	2 (22.2%)	1 (11.1%)	3 (16.7%)
*KRAS*	2 (22.2%)	0 (0%)	2 (11.1%)
*ATM*	1 (11.1%)	0 (0%)	1 (5.6%)
*FANCA*	1 (11.1%)	0 (0%)	1 (5.6%)
*MYC* amplification	0 (0%)	1 (11.1%)	1 (5.6%)
*RB1*	0 (0%)	0 (0%)	0 (0%)
*BRCA 1/2*^a^	0 (0%)	0 (0%)	0 (0%)
*CCNE1 amplification*	0 (0%)	0 (0%)	0 (0%)
*Myt1*	0 (0%)	0 (0%)	0 (0%)
Loss of H3K36me3 biomarker by IHC			
Yes	3 (33.3%)	3 (33.3%)	6 (33.3%)
No	2 (22.2%)	0	2 (11.1%)
Unknown	4 (44.4%)	6 (66.7%)	10 (55.6%)
Baseline sites of involvement, *N* (%)			
Lung/pleura (including effusion)	6 (66.7%)	8 (89.9%)	14 (77.8%)
Lymph nodes (all sites)	5 (55.6%)	6 (66.7%)	11 (61.1%)
Bones	1 (11.1%)	5 (55.6%)	6 (33.3%)
Gastrointestinal	6 (66.7%)	3 (33.3%)	9 (50%)
Hepatobiliary/adrenal	5 (55.6%)	4 (44.4%)	9 (50%)
Kidney (including nephrectomy bed)	1 (11.1%)	3 (33.3%)	4 (22.2%)
Other (thyroid, pericardium, pelvis, and muscle)	2 (22.2%)	5 (55.6%)	7 (38.9%)
Prior therapies			
Surgery	8 (88.9%)	8 (88.9%)	16 (88.9%)
Radiation therapy	5 (55.6%)	4 (44.4%)	9 (50.0%)
Median lines of systemic therapy (range)	2 (1–7)	2 (1–7)	2 (1–7)
Prior types of systemic therapy			
Immunotherapy (anti-PD1, PDL1, and CTLA4)	4 (44.4%)	8 (88.9%)	12 (66.7%)
Tyrosine kinase inhibitor (TKI)	4 (44.4%)	6 (66.7%)	10 (55.6%)
Other chemotherapy	7 (77.8%)	1 (11.1%)	8 (44.4%)

a Co-occurring mutations in DNA damage repair genes other than *ATM*, *BRCA1*, or *BRCA2* (i.e., *PALB2*, *ATR*, *CDK12*, *CHEK1*, *CHEK2*, *RAD51B*, *RAD51C*, and *RAD51D*) were not identified in the entire study population (0%).

**Figure 1:**
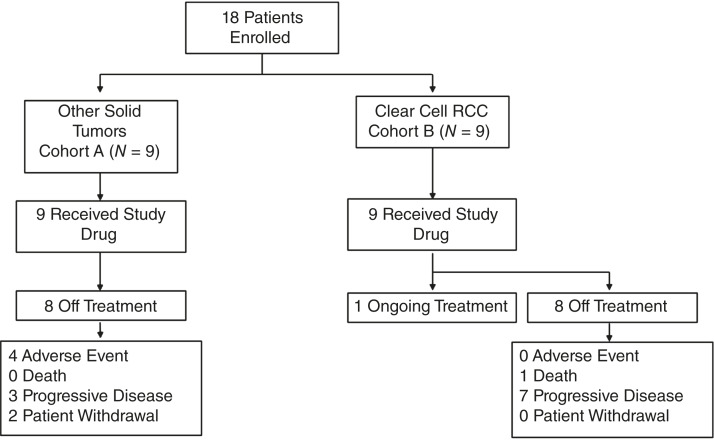
Patient disposition.

Patient disposition is shown in Fig. 1.

**Figure 2:**
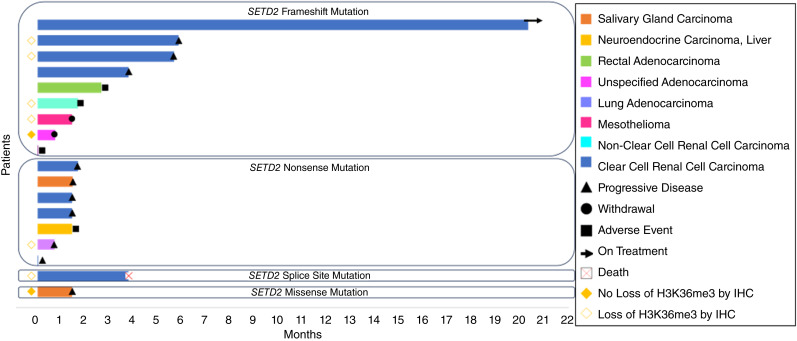
Duration of treatment by *SETD2* mutation type.

The median follow-up for the study population was 20.87 months. Seventeen patients discontinued treatment because of disease progression (*n* = 10), adverse event (*n* = 4), patient withdrawal (*n* = 2), or death (*n* = 1). The median duration of treatment was 1.28 months (range 0.04–21+ months; Fig. 2).

### Treatment efficacy

**Figure 3:**
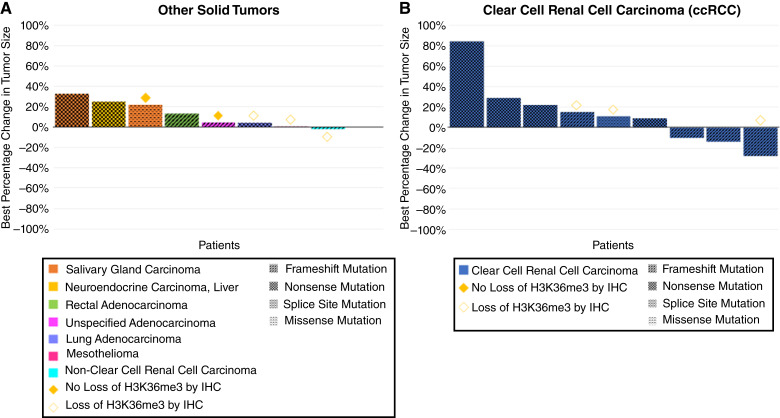
Best percentage change in tumor size in all evaluable patients in cohort A (Other Solid Tumors) and cohort B (ccRCC).

No objective responses were observed in either Cohort A (Other Solid Tumor) or Cohort B (ccRCC) per RECIST 1.1, and thus, study accrual was halted after stage 1 in both cohorts. Minor tumor regressions of any magnitude were observed in 4/18 (22.2%) patients, including one patient with nonclear cell (papillary) RCC, and three patients with ccRCC (Fig. 3).

**Figure 4:**
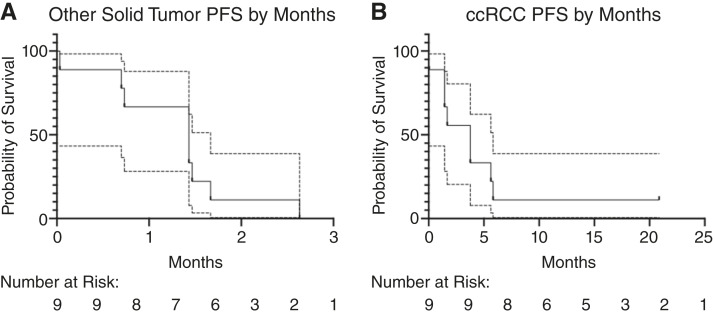
Progression-free survival (PFS) in solid tumor malignancies other than ccRCC (**A**) or in ccRCC (**B**). Dotted lines represent a 95% confidence interval.

In Cohort A (other solid tumors), the investigator-assessed median PFS was 1.43 months (Fig. 4). The median duration of treatment was 1.43 months (range 0.03–2.63) as shown in Fig. 2.

In Cohort B (ccRCC), the investigator-assessed median PFS was 3.77 months (Fig. 4). The median duration of treatment was 3.76 months (range 0.03–20.86) as shown in Fig. 2. SD > 4 months was observed in 3/9 (33.3%) patients with ccRCC, including one patient who remains on treatment after >20 months at the cutoff date.

The reasons for discontinuation of the study drug were because of progressive disease (59%), adverse event (23%), patient withdrawal (12%), or death unrelated to the study drug (6%).

### Treatment safety

Across both cohorts, 3/18 patients (17%) discontinued the study drug because of an adverse event. Of these, one patient (6%) had grade 2 nausea and vomiting that was possibly related to the study drug. Two patients (11%) discontinued for adverse events determined to be unrelated to the study treatment (*n* = 1 venous thromboembolism; *n* = 1 grade 3 paresthesias). Table 2 outlines the most common treatment-emergent adverse events involving ≥25% of patients, which included nausea, anemia, diarrhea, and neutropenia.

**Table 2:** Treatment-emergent adverse events affecting ≥25% of patients

Adverse event	Grade 1 - Number of patients (%)	Grade 2 - Number of patients (%)	Grade 3 - Number of patients (%)	Grade 4 - Number of patients (%)	Grade 5 - Number of patients (%)	Total - Number of patients (%)
Nausea	3 (18%)	7 (41%)	—	—	—	10 (58.8%)
Anemia	3 (18%)	3 (18%)	1 (6%)	—	—	7 (41.2%)
Diarrhea	4 (24%)	2 (12%)	1 (6%)	—	—	7 (41.2%)
Neutropenia	—	1 (6%)	3 (18%)	3 (18%)	—	7 (41.2%)
Fatigue	2 (12%)	4 (24%)	—	—	—	6 (35.3%)
Vomiting	2 (12%)	4 (24%)	—	—	—	6 (35.3%)
Increased creatinine	3 (18%)	1 (6%)	—	1 (6%)	—	5 (29.4%)

### Outcomes by genotypic subtype and loss of H3K36me3 methylation mark

Of 18 patients accrued, only 8/18 (44.4%) had archival tissue submitted to evaluate the loss of H3K36me3 by IHC as an exploratory biomarker correlative analysis (five patients from Cohort A; three patients from Cohort B; Supplementary Figs. S2 and S3). The small number of patients where such tissue was available further limits any signals or conclusions from this exploratory analysis.

In cohort A, 3/5 (60%) evaluable patients demonstrated loss of H3K36me3 by IHC, whereas 2/5 (40%) evaluable patients did not demonstrate this loss. In cohort B, 3/3 (100%) evaluable patients demonstrated loss of H3K36me3 by IHC. Although the sample size was limited, there did not seem to be an association between the loss of trimethylation mark with either tumor regression or duration of treatment on study.

Frameshift, nonsense, missense, and splice site mutations in *SETD2* were identified in nine (50%), seven (39%), one (6%), and one (6%) of the patients, respectively. Although the small sample size precluded statistical evaluation, there seemed to be a higher probability of clinical benefit with respect to the duration of treatment among those patients with frameshift mutations in *SETD2*.

## Discussion

Prior preclinical studies demonstrated a significant synthetic lethal effect with single-agent WEE1 inhibition in *SETD2*-deficient cancer models including ccRCC (16). Although therapeutic development of adavosertib has been in combination with other therapies (DNA damage repair targets such as PARP inhibitors and cytotoxic chemotherapy), we sought to evaluate single-agent activity in *SETD2*-altered solid tumor malignancies with a particular emphasis on ccRCC, given the significant and durable tumor regressions observed in preclinical studies, with the potential to have less adverse events and improved toxicity profiles compared with its use in combination with other cancer-directed therapies. Despite the compelling scientific rationale and preclinical data, we did not observe strong evidence of synthetic lethal effect clinically, as there were no objective responses observed despite pathogenic mutations in the *SETD2* gene. There was, intriguingly, a subset of patients who experienced clinical benefits with durable stable disease on treatment lasting for more than 4 months, including one patient with ongoing stable disease for more than 2 years.

The limited clinical activity observed with single-agent adavosertib in the current study is consistent with the prior results of this agent and other single-agent WEE1 inhibitors in genomically unselected patient populations. A phase Ib study of adavosertib in 80 patients with ovarian cancer, triple-negative breast cancer, or small-cell lung cancer demonstrated ORRs between 0% and 6.3% (23). Another phase Ib study of adavosertib monotherapy in 62 patients with various malignancies noted an ORR of 3.4% (2/58 evaluable patients) with only partial responses observed (24). These are lower response rates compared with another phase II study of adavosertib monotherapy in 34 evaluable patients with uterine serous carcinoma (USC) demonstrated an ORR of 29.4%, with one complete response and nine partial responses, although this is possibly because of the increased replicative stress of USC and subsequent increased susceptibility to WEE1 inhibition (25). In another phase II study of adavosertib monotherapy in patients with *RAS/TP53*-mutant metastatic colorectal cancer demonstrated improved PFS but only one patient with a partial response was noted. In a previously published abstract, azenosertib (formerly known as ZN-c3), another WEE1 inhibitor, was evaluated in a phase I study of 39 patients with advanced or metastatic solid tumors refractory to standard therapy. Of 16 evaluable patients, five had stable disease and two had partial responses (ORR = 12.5%), suggesting clinical activity (26). In another previously published abstract of ZN-c3 in patients with advanced/recurrent USC, the phase I study demonstrated an ORR of 12.5%, with partial responses observed in 3/12 evaluable patients (27).

Overall, the safety and tolerability profile were similar to that reported in various phase I/II studies of adavosertib monotherapy. There were similar frequencies of fatigue, nausea, vomiting, diarrhea, and anemia when compared with other phase II studies of adavosertib monotherapy in genomically unselected and *TP53*-mutant cancers (25, 28, 29).

We chose to utilize an intermittent dosing schedule of adavosertib, based on the safety profile observed in prior studies, with the goal of limiting treatment breaks that were required to lessen the degree of hematologic toxicity with continuous dosing (17). Takebe and colleagues previously showed that once-daily adavosertib maximum tolerated dose (MTD) at 300 mg QDAY exhibited a dose-proportional pharmacokinetic profile similar to the twice-daily MTD (225 mg BID), with plasma concentrations being higher on day 5 compared with day 1 (17). With an approximately 11-hour half-life for this agent, the MTD for once-daily adavosertib yielded comparable plasma exposures to the MTD for the twice-daily regimen (17). From a pharmacodynamic standpoint, an intermittent dosing schedule may potentially limit antitumor activity because of partial recovery of WEE1 enzymatic activity after the dosing interval, with a possible target rebound effect during the dosing break, which has also been observed in other kinase inhibitors as noted by Takebe and colleagues (17). Additional investigation is needed to determine any relationship between target recovery and antitumor activity.

Interestingly, all four patients who had modest tumor regression had pathogenic frameshift mutations in *SETD2*, compared with nonsense, missense, or splice site mutations, along with a trend toward longer treatment duration with frameshift mutations. In the entire study population, one patient with rectal cancer and one patient with ccRCC had a co-mutated *TP53* pathogenic alteration, although none of them had tumor regression. The functional impact of different classes of genomic alterations in the *SETD2* gene warrants further investigation.

This study allowed any patient with a pathogenic *SETD2* alteration, without a variant allele frequency cutoff; thus, we were unable to verify the presence of biallelic loss of *SETD2*. Although only one patient in our study population had a missense mutation, and another one patient with a splice-site mutation, it is unclear the impact on the response that a biallelic loss of function of *SETD2* could have made. In patients with ccRCC demonstrating a sequenced alteration of *SETD2* (i.e., frameshift), it can be presumed that the pathogenic *SETD2* loss is biallelic due to the ubiquitous 3p loss in this tumor type. Monoallelic loss of *SETD2* is not sufficient to disrupt H3K36 methylation, which may account for the preserved trimethylation mark in 2/8 evaluable archival tissue samples confirmed to harbor a *SETD2* mutation. Loss of trimethylation mark of H3K36me3 was observed in the majority of evaluable tumors but did not seem to be associated with clinical outcomes. However, the sample size of evaluable tumors was small to fully appreciate any potential signal or derive any conclusions. The lack of available tissue for the exploratory biomarker is a limitation of this study. In addition, this study did not obtain ontreatment biopsies for documentation of the loss of RRM2, which may have been required for synthetic lethal effects (compared with what was seen in the preclinical models). Further investigation is needed to determine if other genomic or proteomic biomarkers could identify a subset of patients who could derive benefit from WEE1 inhibition given as monotherapy.

With the small size of the study population, we are also not able to fully evaluate and explore the impact of concurrent pathogenic alterations with *SETD2* that may impact the efficacy of adavosertib monotherapy. For example, only 3/18 (16.7%%) of patients had each of the following concurrent pathogenic mutations: *TP53* mutations and *CDKN2A/B* loss. Two patients (11.1%) did have a *KRAS* mutation. Moreover, 1/18 (5.6%) of patients had each of the following concurrent pathogenic mutations: *ATM*, *MYC* amplification. There were no *CCNE1* amplification or *MYT1* mutations. Prior studies have reported possible increased response to adavosertib in *TP53*-mutant or *CCNE1*-amplified solid tumor malignancies, and possible resistance to adavosertib via *MYT1* overexpression (17, 29–31). Adavosertib in combination with carboplatin has demonstrated clinical benefit with an ORR of 41% in patients with *TP53*-mutant, platinum-resistant ovarian cancer (29). Thus, additional identifying potential biomarkers to help better select patients who may respond to WEE1 inhibition, including the possibility of using *PTEN* as a biomarker for efficient WEE1 cancer therapy (32)

Lastly, the sample size of this study was small but lacked racial/ethnic diversity, further limiting the generalizability of these data. This warrants subsequent systems improvement to enhance underrepresented minority patient recruitment into clinical trials.

Overall, therapeutic targets for patients with *SETD2*-mutated cancers are still an unmet clinical need warranting further investigation. There are several ongoing clinical trials of WEE1 inhibitors in combination with other agents including cytotoxic chemotherapy and/or radiation (NCT03028766, NCT03012477, NCT06015659, NCT01164995, NCT02194829, NCT02101775, NCT02906059, NCT02037230, NCT04460937, NCT05815160, NCT03345784, NCT05765812, and NCT02341456). There could be some potential for investigating WEE1 inhibitors in combination with other DNA damage repair targeting agents (e.g., ATR inhibitors and CHK inhibitors). In addition, further investigation of predictive biomarkers is needed for the selection of patients with *SETD2* alterations who may derive a response to WEE1 inhibitors alone or in combination with other cancer-directed therapies.

## Supplementary Material

Supplementary Protocol S1 Supplementary Protocol S1 shows the entire clinical trial protocol to supplement the Materials and Methods section.

Supplementary Figure S2 Supplementary Figure S2 shows an example of no loss of H3K36me3 by IHC (A) and loss of H3K36me3 by IHC (B). Images are at 40x power. Of note, the portal did not allow for uploading of a TIFF file alone as a "supplemental data" file, so the image included in this document is a TIFF file. We can easily provide the TIFF file if needed as well.

Supplementary Figure S3 Supplementary Figure S3 shows all 8 patients with H3K36me3 IHC analysis. (A) Cohort A: Other Solid Tumor. (B) Cohort B: ccRCC. Images are at 40x power. Of note, the portal did not allow for uploading of a TIFF file alone as a "supplemental data" file, so the image included in this document is a TIFF file. We can easily provide the TIFF file if needed as well.

Supplementary Table S4 Supplementary Table S4 is a table showing the representativeness of study participants.
