# Supplementary material for: A Phase II Trial of the WEE1 Inhibitor Adavosertib in SETD2-Altered Advanced Solid Tumor Malignancies (NCI 10170)
Source: Cancer Res Commun. 2024 Jul 23;4(7):1793–801. doi: 10.1158/2767-9764.CRC-24-0213 (PMC11264598; doi:10.1158/2767-9764.CRC-24-0213)
Supplement: Supplementary Protocol S1 — shows the entire clinical trial protocol to supplement the Materials and Methods section. [file crc-24-0213_supplementary_protocol_s1_supps1.pdf]

SUMMARY OF CHANGES – Protocol

For Protocol Revision to: A Phase 2 Study of AZD1775 in SETD2-Deficient Advanced Solid Tumor Malignancies

NCI Protocol #: 10170
Local Protocol #: 18952

NCI Version Date: 06-05-2020
Protocol Date: 06-05-2020

I. Administrative Changes by Principal Investigator:

#	Section	Comments
1.	*Header*	Version date in protocol header updated to 06-05-2020
2.	*Face Page*	Updated: Protocol Type / Version # / Version Date

NCI Protocol #: 10170

Local Protocol #: 18952

ClinicalTrials.gov Identifier: NCT03284385

TITLE: A Phase 2 Study of AZD1775 in *SETD2*-Deficient Advanced Solid Tumor Malignancies

Corresponding Organization: **LAO-CT018** / Yale University Cancer Center LAO

Principal Investigator: Rahul Aggarwal
CA824-UCSF Medical Center-Mission Bay
Mission Hall, 550 16th Street, Sixth Floor Box 3211
San Francisco, CA-94143 USA
(415) 353-9278
Rahul.Aggarwal@ucsf.edu

Participating Organizations

**LAO-11030** / University Health Network Princess Margaret Cancer Center LAO
**LAO-CA043** / City of Hope Comprehensive Cancer Center LAO
**LAO-MA036** / Dana-Farber - Harvard Cancer Center LAO
**LAO-MD017** / JHU Sidney Kimmel Comprehensive Cancer Center LAO
**LAO-MN026** / Mayo Clinic Cancer Center LAO
**LAO-NC010** / Duke University - Duke Cancer Institute LAO
**LAO-NJ066** / Rutgers University - Cancer Institute of New Jersey LAO
**LAO-OH007** / Ohio State University Comprehensive Cancer Center LAO
**LAO-PA015** / University of Pittsburgh Cancer Institute LAO
**LAO-TX035** / University of Texas MD Anderson Cancer Center LAO
**LAO-NCI** / National Cancer Institute LAO
**EDDOP** / Early Drug Development Opportunity Program

Study Coordinator:
Kathleen Comerford
CA824-UCSF Medical Center-Mission Bay
550 16th Street, San Francisco, CA-94158 USA
Kathleen.Comerford@ucsf.edu
(415) 353-9535

NCI-Supplied Agent: AZD1775 NSC # 751084

IND #: 116495

IND Sponsor: DCTD, NCI

Protocol Type / Version # / Version Date: Original/ Version 1.0 / August 8, 2017
Revision / Version 2.0 / September 11, 2017
Revision / Version 3.0 / October 6, 2017
Revision / Version 4.0 / December 17, 2017
Revision / Version 5.0 / January 28, 2018
Revision / Version 6.0 / February 28, 2018
Revision / Version 7.0 / April 18, 2018
Amendment 1 / Version 8.0 / July 12, 2018
Amendment 2 / Version 9.0 / January 20, 2019
Amendment 3 / Version 10.0 / June 26, 2019
Amendment 4 / Version 11.0 / December 11, 2019
Amendment 5 / Version 12.0 / February 07, 2020
Amendment 6 / Version 13.0 / May 26, 2020
Amendment 7 / Version 14.0 / June 05, 2020

SCHEMA

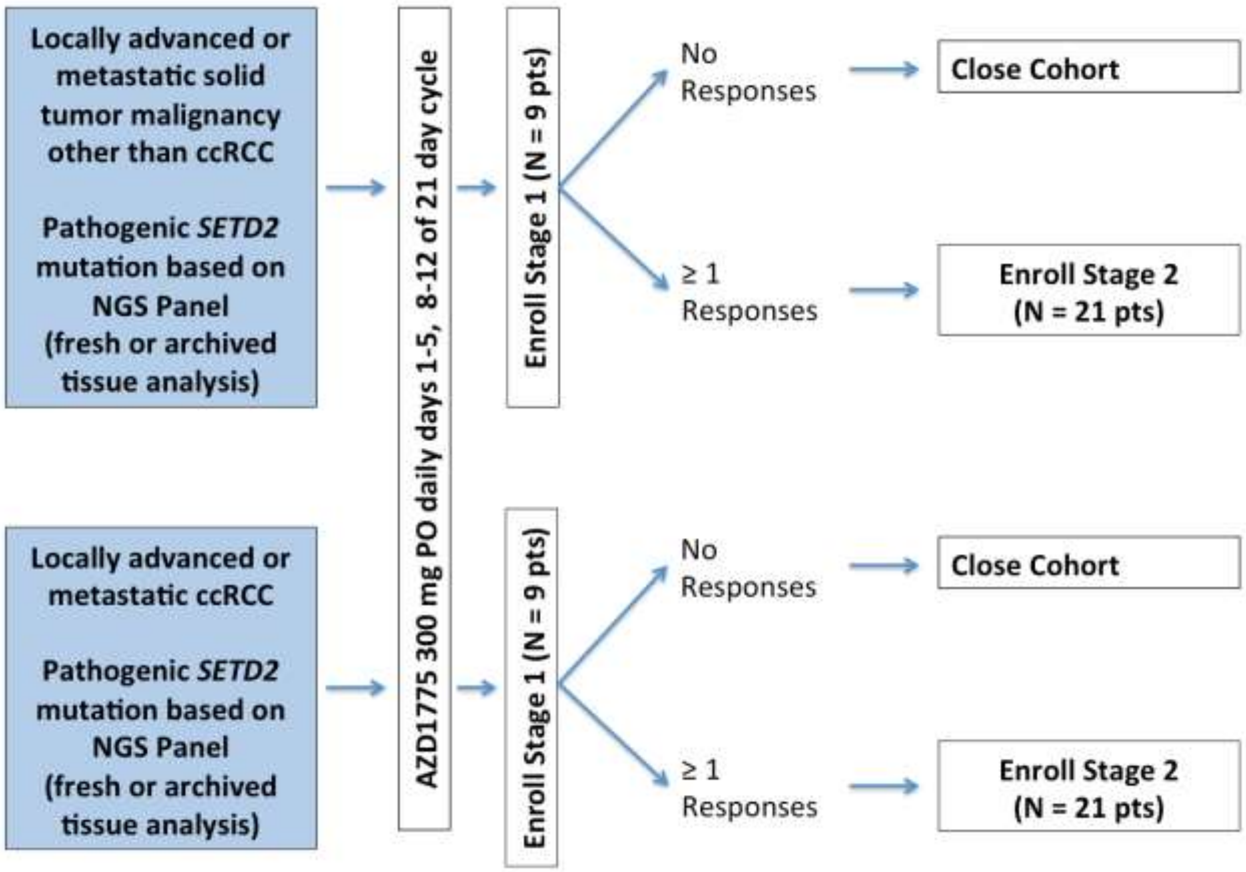
Locally advanced or metastatic solid tumor malignancy other than ccRCC
Pathogenic SETD2 mutation based on NGS Panel (fresh or archived tissue analysis)
Locally advanced or metastatic ccRCC
Pathogenic SETD2 mutation based on NGS Panel (fresh or archived tissue analysis)
AZD1775 300 mg PO daily days 1-5, 8-12 of 21 day cycle
Enroll Stage 1 (N = 9 pts)
No Responses
Close Cohort
≥ 1 Responses
Enroll Stage 2 (N = 21 pts)
Enroll Stage 1 (N = 9 pts)
No Responses
Close Cohort
≥ 1 Responses
Enroll Stage 2 (N = 21 pts)

TABLE OF CONTENTS

SCHEMA 3

1. OBJECTIVES 6
   1.1 Primary Objectives 6
   1.2 Secondary Objectives 6

2. BACKGROUND 6
   2.1 Synthetic Lethal Effect of WEE1 inhibition in SETD2-Deficient Tumors 6
   2.2 AZD1775 14
   2.3 Rationale 20
   2.4 Correlative Studies Background 21

3. PATIENT SELECTION 22
   3.1 Inclusion Criteria 22
   3.2 Exclusion Criteria 24
   3.3 Inclusion of Women and Minorities 26

4. REGISTRATION PROCEDURES 27
   4.1 Investigator and Research Associate Registration with CTEP 27
   4.2 Site Registration 28
   4.3 Patient Registration 30
   4.4 General Guidelines 31

5. TREATMENT PLAN 31
   5.1 AZD1775 Administration 31
   5.2 General Concomitant Medication and Supportive Care Guidelines 33
   5.3 Prohibited concomitant mediations 33
   5.4 Duration of Therapy 35
   5.5 Duration of Follow Up 36

6. DOSING DELAYS/DOSE MODIFICATIONS 36

7. ADVERSE EVENTS: LIST AND REPORTING REQUIREMENTS 40
   7.1 Comprehensive Adverse Events and Potential Risks List (CAEPR) 41
   7.2 Adverse Event Characteristics 46
   7.3 Expedited Adverse Event Reporting 46
   7.4 Routine Adverse Event Reporting 48
   7.5 Pregnancy 48
   7.6 Secondary Malignancy 49

8. PHARMACEUTICAL INFORMATION 49
   8.1 AZD1775 49

9. BIOMARKER, CORRELATIVE, AND SPECIAL STUDIES 52
   9.1 Integral biomarkers 52

9.2 Integrated Correlative Studies ... 53

10. STUDY CALENDAR ... 56

11. MEASUREMENT OF EFFECT ... 57
11.1 Antitumor Effect – Solid Tumors ... 57
11.2 Other Response Parameters ... 63

12. INFORMED CONSENT, STUDY OVERSIGHT AND DATA REPORTING / REGULATORY REQUIREMENTS ... 63
12.1 Informed Consent ... 63
12.2 Study Oversight ... 63
12.3 Data Reporting ... 64
12.4 Collaborative Agreements Language ... 66

13. STATISTICAL CONSIDERATIONS ... 67
13.1 Study Design/Endpoints ... 67
13.2 Sample Size/Accrual Rate ... 68
13.3 Stratification Factors ... 69
13.4 Analysis of Secondary Endpoints ... 69
13.5 Reporting and Exclusions ... 70

REFERENCES ... 71

APPENDIX A PERFORMANCE STATUS CRITERIA ... 73

APPENDIX B PATIENT DRUG INFORMATION HANDOUT AND WALLET CARD ... 74

APPENDIX C STUDY DRUG DIARY ... 76

1. OBJECTIVES

1.1 Primary Objectives

A) To determine the objective response rate by RECIST 1.1 criteria of AZD1775 in advanced solid tumor malignancies other than clear cell renal cell carcinoma with evidence of pathogenic loss of *SETD2* using next-generation sequencing panel.

B) To determine the objective response rate by RECIST 1.1 criteria of AZD1775 in clear cell renal cell carcinoma with evidence of loss of *SETD2* using next-generation sequencing panel.

1.2 Secondary Objectives

1.2.1 To determine the clinical benefit rate and duration of response of AZD1775 in *SETD2*-deficient tumors other than clear cell renal cell carcinoma.

1.2.2 To determine the clinical benefit rate and duration of response of AZD1775 in *SETD2*-deficient clear cell renal cell carcinoma subgroup.

1.2.3 To characterize the safety profile of AZD1775

1.2.4 To determine whether the H3K36me3 mark by immunohistochemical assay is associated with clinical outcomes.

2. BACKGROUND

2.1 Synthetic Lethal Effect of WEE1 inhibition in SETD2-Deficient Tumors

The *SETD2* tumor suppressor gene encodes for a histone H3K36 methyltransferase that has been observed in TCGA and other molecular tumor profiling studies to sustain pathogenic loss-of-function mutations across a broad range of solid tumor malignancies, including in 13% of renal cell carcinoma, 20% of pancreatic neuroendocrine tumors, and 5-10% of other common cancer subtypes including non-small cell lung, colorectal, bladder, uterine and pancreatic adenocarcinoma **(Figure 1).** Frequent biallelic loss is observed via loss of heterozygosity, for example with concomitant 3p chromosome loss observed in the vast majority of renal cell carcinomas. Summed across malignancies, SETD2 mutant-tumors comprise a non-trivial proportion of patients with advanced solid tumor

Tumor Type	*SETD2* Mutation Frequency (%)
Pancreatic NET	21
Renal Cell Carcinoma (clear cell)	13
Lung adenocarcinoma	9
Endometrial	9
Bladder	7
Cholangiocarcinoma	6
Colorectal	5
Melanoma	5
Esophageal	4
Head and Neck Squamous Cell Carcinoma	3
Pancreatic adenocarcinoma	2
Breast cancer	2
Ovarian cancer	2

Source: TCGA/cBioPortal

Figure 1. Frequency of SETD2 mutations by tumor type.

malignancies for which there remain limited therapeutic options.

To our knowledge, there have been no prior clinical studies specifically targeting *SETD2*-mutant tumors. There is a clear and currently unmet medical need to develop novel targeted therapeutics targeting solid tumor malignancies characterized by these pathogenic alterations. Based on the pre-clinical data stemming from the Ashworth laboratory at UCSF, we propose to clinically investigate the WEE1 inhibitor (AZD1775) as a synthetically lethal, clinically available, systemic therapeutic option for solid tumor malignancies driven by these molecular alterations.

<u>WEE1 Inhibition Demonstrates a Synthetic Lethal Effect in SETD2-Deficient Cancers:</u>
SETD2 was first identified as a tumor suppressor in 2010 (Duns et al., 2010) and its proposed functions include DNA repair (Aymard et al., 2014; Carvalho et al., 2014; Pfister et al., 2014), chromatin structure modulation during transcription (Carvalho et al., 2013), and stem cell regulation (Zhang et al., 2014; Zhu et al., 2014). SETD2 mutations and deletions lead to genome-wide loss of histone H3K36 trimethylation (H3K36me3) and are associated with poor prognosis in breast cancer (Al Sarakbi et al., 2009) and renal cancer (Hakimi et al., 2013). Despite its frequent loss and association with poor prognosis, there are currently no therapies in development targeting SETD2-deficient cancers.

The WEE1 kinase inhibits the activities of cyclin-dependent kinases CDK1 and CDK2 through tyrosine 15 phosphorylation (Parker and Piwnica-Worms, 1992; Watanabe et al., 1995). Inhibition of WEE1 promotes unscheduled mitotic entry through CDK1 activation, leading to loss of genome integrity (Tominaga et al., 2006).

With four different approaches (Pfister et al., 2015), it has demonstrated that SETD2-deficient cancer cells are hypersensitive to WEE1 inhibition **(Figure 2)**. First, they found that two naturally occurring SETD2-deficient cell lines (A498 and LB996) were hypersensitive to AZD1775 (A498 half-maximal inhibitory concentration [IC50] = 87 nM, LB996 IC50 = 68 nM versus RCC4 IC50 = 673 nM, U2OS IC50 = 712 nM) ($p < 0.0001$) **(Figure 2A and 2B**). A498, LB996, and RCC4 are renal cell carcinoma cell lines; U2OS is an osteosarcoma cell line that is suitable for genetic manipulation. A498 expresses a near full-length non-functional SETD2 protein, whereas LB996 does not express the SETD2 protein.

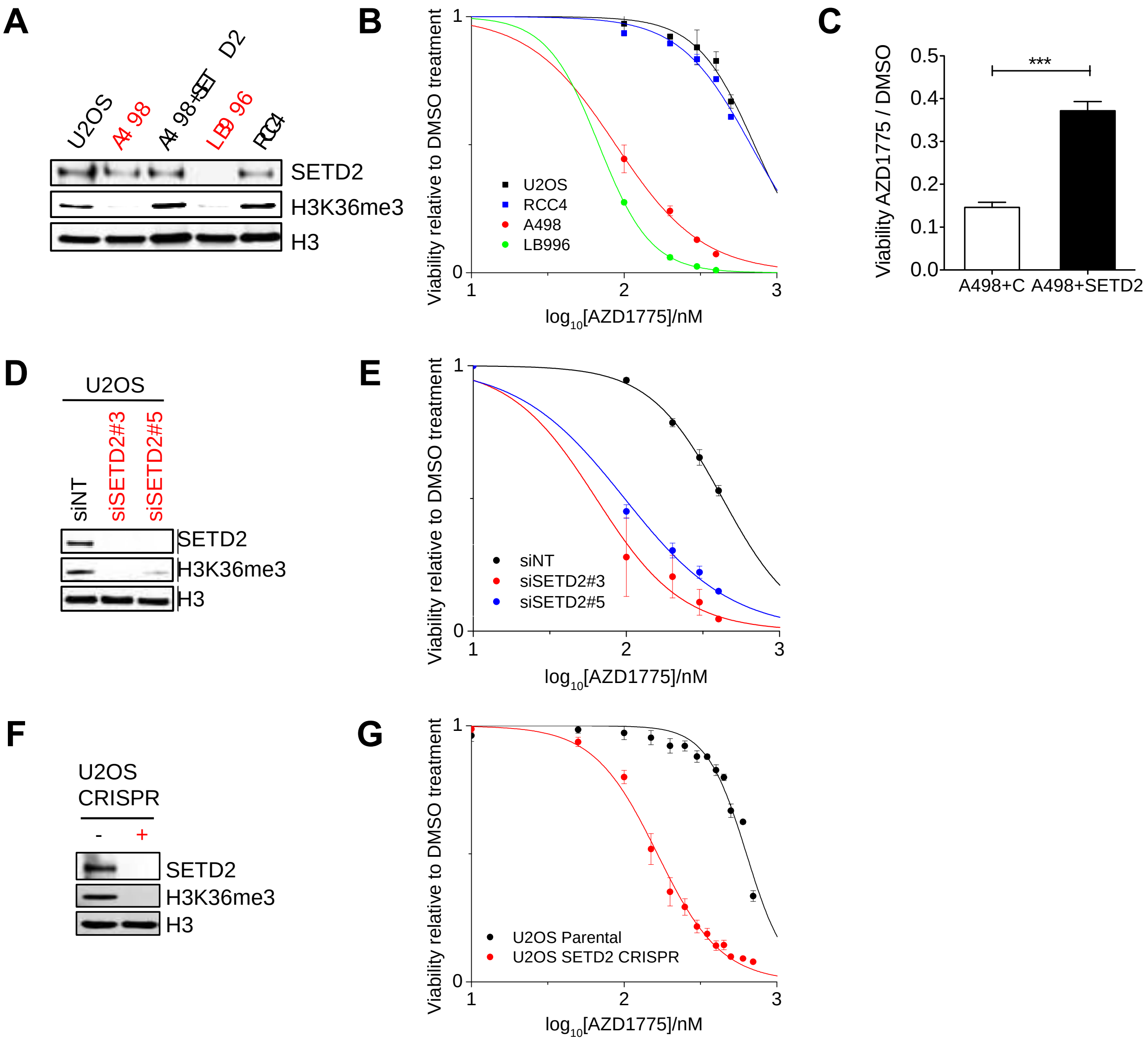

Figure 2. SETD2-deficient cancer cells are hypersensitive to WEE1 inhibition by AZD1775. A498 and LB996 cell lines are SETD2-deficient; RCC4 and U2OS cell lines are SETD2 wild-type. A498, LB996, and RCC4 are renal cell carcinoma cell lines; U2OS is an osteosarcoma cell line that is suitable for genetic manipulation. A498 expresses a near full-length non-functional SETD2 protein, whereas LB996 does not express the SETD2 protein. **(A)** Western blot analysis of SETD2 and H3K36me3 (the histone mark catalyzed by SETD2) levels in A498, LB996, RCC4 and U2OS cells. A498 + SETD2 are A498 cells stably expressing a *SETD2* cDNA. **(B)** Viability curves of SETD2 wild-type (RCC4, U2OS) and SETD2-deficient (A498, LB996) cells after exposure to WEE1 inhibitor AZD1775 (5 days). **(C)** Viability of A498 cells expressing either an empty vector (A498 + C) or *SETD2* cDNA (A498 + SETD2) after exposure to AZD1775 (200 nM) (72 hr). **(D)** Western blot analysis of SETD2 and H3K36me3 in U2OS cells transfected with either control siRNA (siNT) or SETD2 siRNAs (siSETD2#3, siSETD2#5). **(E)** Viability curves of U2OS cells transfected with either control siRNA (siNT) or SETD2 siRNAs (siSETD2#3 and siSETD2#5) (48 hr) and exposed to AZD1775 (5 days). **(F)** Western blot analysis of SETD2 and H3K36me3 levels in U2OS parental cells or U2OS cells with CRISPR knockout of SETD2. **(G)** Viability curves of U2OS parental cells and U2OS SETD2 CRISPR knockout cells after exposure to AZD1775 (5 days). Data are presented as mean ± SEM, n = 3 independent experiments. ***$p < 0.001$, unpaired and two-tailed t test was used.

Second, expressing *SETD2* cDNA in A498 cells restored H3K36me3 levels and reduced sensitivity to AZD1775 **(Figure 2A and 2C)**. Third, SETD2 knockdown with two independent siRNAs sensitized cells to AZD1775 **(Figure 2D and 2E)**. Lastly, they generated a SETD2-knockout cell line using CRISPR technology, where the gRNA-guided DNA break led to a frameshift mutation and a premature stop codon in both *SETD2* alleles, resulting in loss of the SETD2 protein. The SETD2-knockout U2OS cells were hypersensitive to AZD1775 compared to the parental SETD2 wild-type U2OS cells (CRISPR IC50 = 151 nM versus parental IC50 = 615 nM) ($p < 0.0001$) **(Figure 2F and 2G)**.

This effect was not only due to growth inhibition, but also cell killing, as evidenced by a 12-fold difference in clonogenic survival (CRISPR IC_{50} = 10 nM versus parental IC50 = 128 nM), and an up to 8-fold increase in apoptosis. Moreover, siRNA knockdown of WEE1 selectively killed CRISPR SETD2-knockout cells, and combining AZD1775 and WEE1 siRNA showed epistasis, confirming that it is WEE1 inhibition that selectively kills H3K36me3-deficient cells. WEE1 is inhibited by AZD1775 by western blotting with pCDK1 Tyr15 and pan-CDK substrates, and that at the doses used, AZD1775 was not inhibiting MYT1 (a kinase related to WEE1). Together, results from the four different approaches above strongly suggest a synthetic lethal interaction between H3K36me3 loss and WEE1 inhibition.

WEE1 Inhibition Abolishes DNA Replication in SETD2-Deficient Cells:
The mechanism underlying this selective killing of SETD2-deficient cells is related to inhibition of DNA replication during the S-phase of the cell cycle. The WEE1 inhibitor AZD1775 forced 32% of the SETD2-knockout cells to accumulate as non-replicating S-phase cells whereas it had no effect on U2OS parental cells **(Figure 3A)**.

The mechanism by which WEE1 inhibition abolishes DNA replication in SETD2-deficient cells was shown to be the depletion of dNTP (the building blocks of DNA) via depletion of RRM2, a ribonuclease reductase subunit essential for the production of dNTP. Both dNTP and RRM2 protein levels were reduced by both AZD1775 treatment and SETD2 knockdown (siSETD2), and combining them (siSETD2 + AZD1775) further depleted dNTP and RRM2 **(Figure 3B and 3C)**, whereas the expression of the other two subunits of ribonuclease reductase (RRM1 and P53R2) were unaffected **(Figure 3C)**.

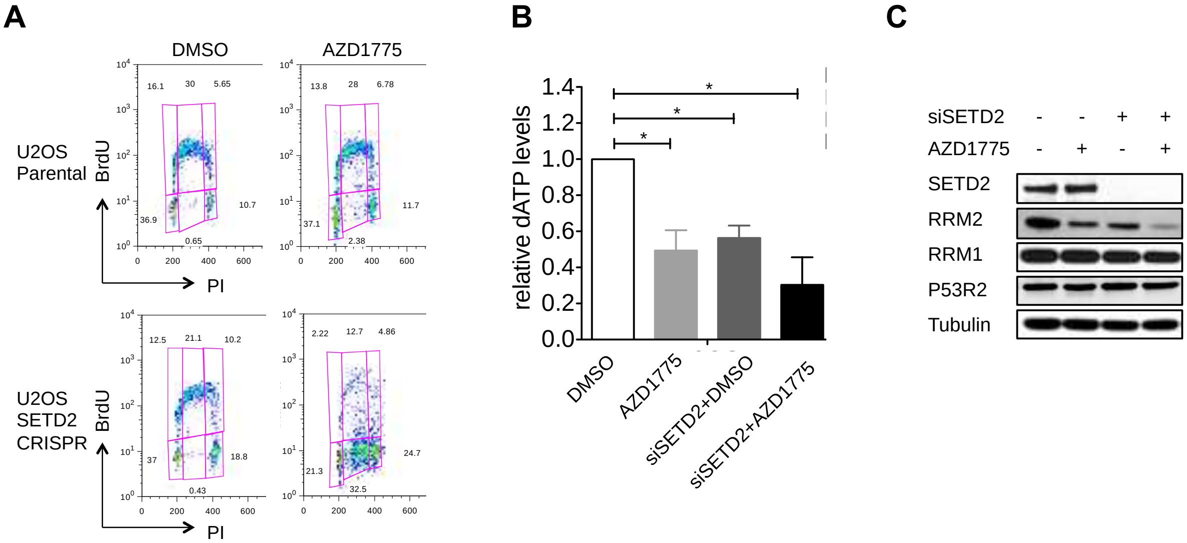

Figure 3. WEE1 inhibitor AZD1175 abolishes DNA replication in SETD2-deficient cells through dNTP starvation by depleting the ribonuclease reductase subunit RRM2. (A) BrdU FACS analysis of the cell cycle distribution of U2OS SETD2 wild-type and U2OS SETD2 CRISPR knockout cells after exposure to DMSO (0.02%) or AZD1775 (200 nM) (48 hr). **(B)** dATP levels in U2OS cells transfected with control siRNA (siNT) or SETD2 siRNA (siSETD2) (48 hr) prior to treatment with either DMSO or AZD1775 (200 nM) (24 hr). Data are normalized to the control (siNT + DMSO). **(C)** Western blot analysis of RRM2, RRM1, and P53R2 protein levels in U2OS cells transfected with control (siNT) or SETD2 siRNA (siSETD2) (48 hr) and exposed to either DMSO or AZD1775 (200 nM) (24 hr).

RRM2 levels are regulated by two mechanisms: (1) SETD2-mediated *RRM2* transcription via binding of H3K36me3 to the promoter of the *RRM2* gene **(Figure 4A)**, and (2) WEE1-mediated protection of RRM2 protein from degradation via inhibition of CDK1/2 **(Figure 4B)**. Therefore, our current model to explain the synthetic lethal effect of WEE1 inhibition in SETD2-deficient tumors converges upon decreased level of the RRM2 subunit of the ribonuclease reductase complex **(Figure 4C)**. This conclusion is bolstered by the findings of decreased RRM2 expression in SETD2-deficient cell lines, and the ability of exogenous expression of RRM2 to rescue the synthetic lethal effect of WEE1 inhibition.

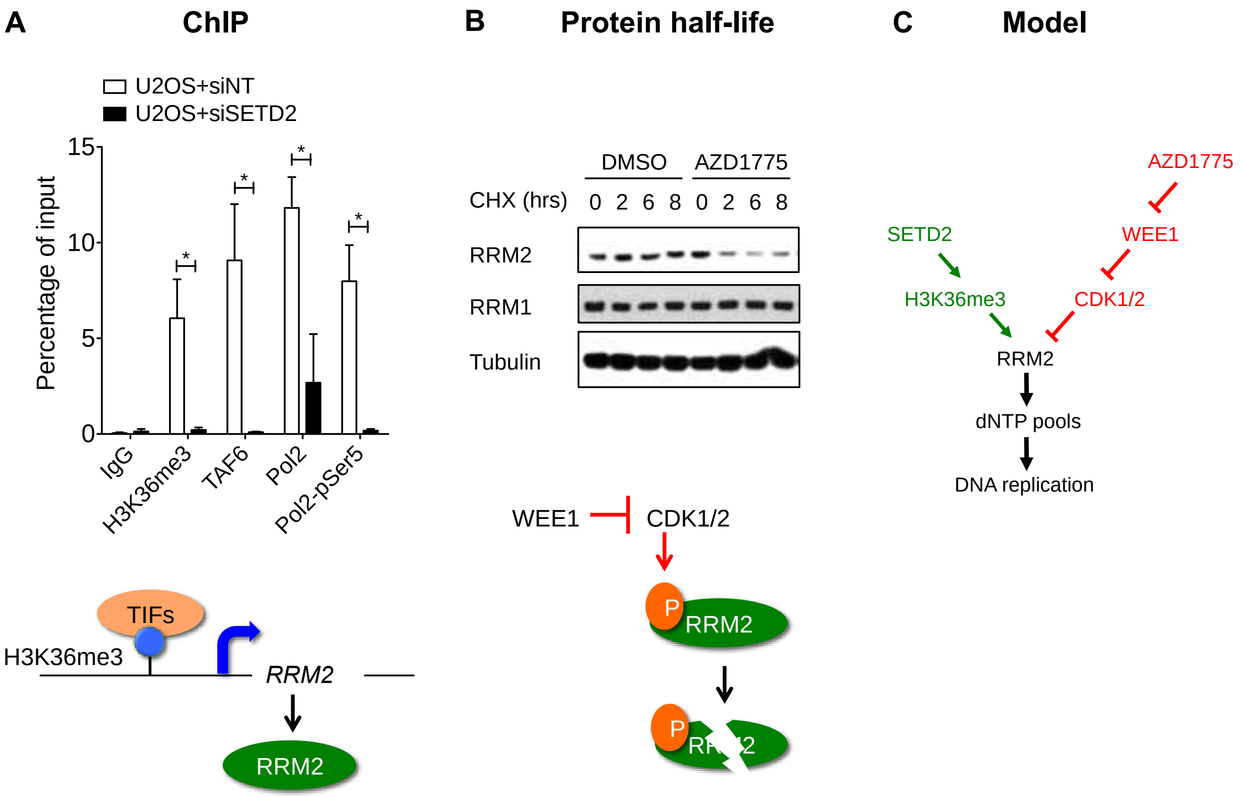

Figure 4. SETD2 deficiency reduces RRM2 transcription while WEE1 inhibition promotes RRM2 degradation. (A) ChIP analysis of the enrichment of H3K36me3, TAF6, RNA-Pol2, and phospho-Pol2 (Ser5) at the *RRM2* promoter in U2OS cells transfected with control siRNA (siNT) or SETD2 siRNA (siSETD2). The qPCR data are presented as percentage of input. **(B)** RRM2 and RRM1 protein half-life analysis. U2OS CRISPR SETD2-knockout cells were treated with cycloheximide (50 µg/ml), which blocks protein synthesis, in the presence of DMSO or AZD1775 (400 nM). Cells were collected at the indicated times, lysed, and immunoblotted as indicated. **(C)** Schematic overview of the synthetic lethal interaction between H3K36me3 loss and WEE1 inhibition. RRM2 is regulated by two pathways. In the first, SETD2 catalyzes histone H3K36me3, which promotes *RRM2* expression. In the second, WEE1 negatively regulates CDK activity and upon WEE1 inhibition, hyperactive CDK promotes RRM2 degradation. Therefore, WEE1 inhibition in H3K36me3-deficient cells leads to critically reduced dNTP pool levels, resulting in replication stress and cell death.

<u>AZD1775 Monotherapy Leads to Significant Tumor Regression in a SETD2-Deficient Xenograft Model</u>

To test the *in vivo* efficacy of AZD1775, tumor xenografts were established in nude mice. Upon AZD1775 treatment (60 mg/kg, bid), all seven tumors generated from SETD2-deficient A498 cells regressed from day 3 onward, resulting in a 5.8-fold reduction in tumor size compared to vehicle-treated control animals (tumor size at day 12 = 50.2 ± 4.7 mm3 versus 291.2 ± 40.0 mm3, p < 0.0001) **(Figures 5A and 5B)**. Consistent with the observation from A498 cells, upon AZD1775 treatment, all five tumors generated from another SETD2-deficient cell line (LB996) regressed from day 3 onward, resulting in a 4.7-fold reduction in tumor size compared to vehicle-treated control animals (tumor size at day 12 = 66.5 ± 15.3 mm3 versus 313.3 ± 40.2 mm3, p < 0.0001) **(Figure 5A).**

In contrast, AZD1775 treatment of tumors generated from SETD2-proficient U2OS cells did not have any significant effect on tumor growth (tumor size at day 12 = 257.5 ± 19.3mm3 versus 292.5 ± 34.3mm3, p = 0.37) **(Figures 5A and 5B).**

Consistent with the *in vitro* data, AZD1775-treated SETD2-deficient tumors showed significantly greater levels of replication stress (measured by gH2AX pan-nuclear staining) **(Figures 5C and 5D),** accompanied by increased apoptosis (measured by cleaved caspase-3) compared with vehicle-treated control **(Figures 5E and 5F).** Importantly, the serum concentration of AZD1775 in these xenograft murine studies was comparable with that achieved with the recommended phase 2 dose (225 mg orally BID) in patients (see below).

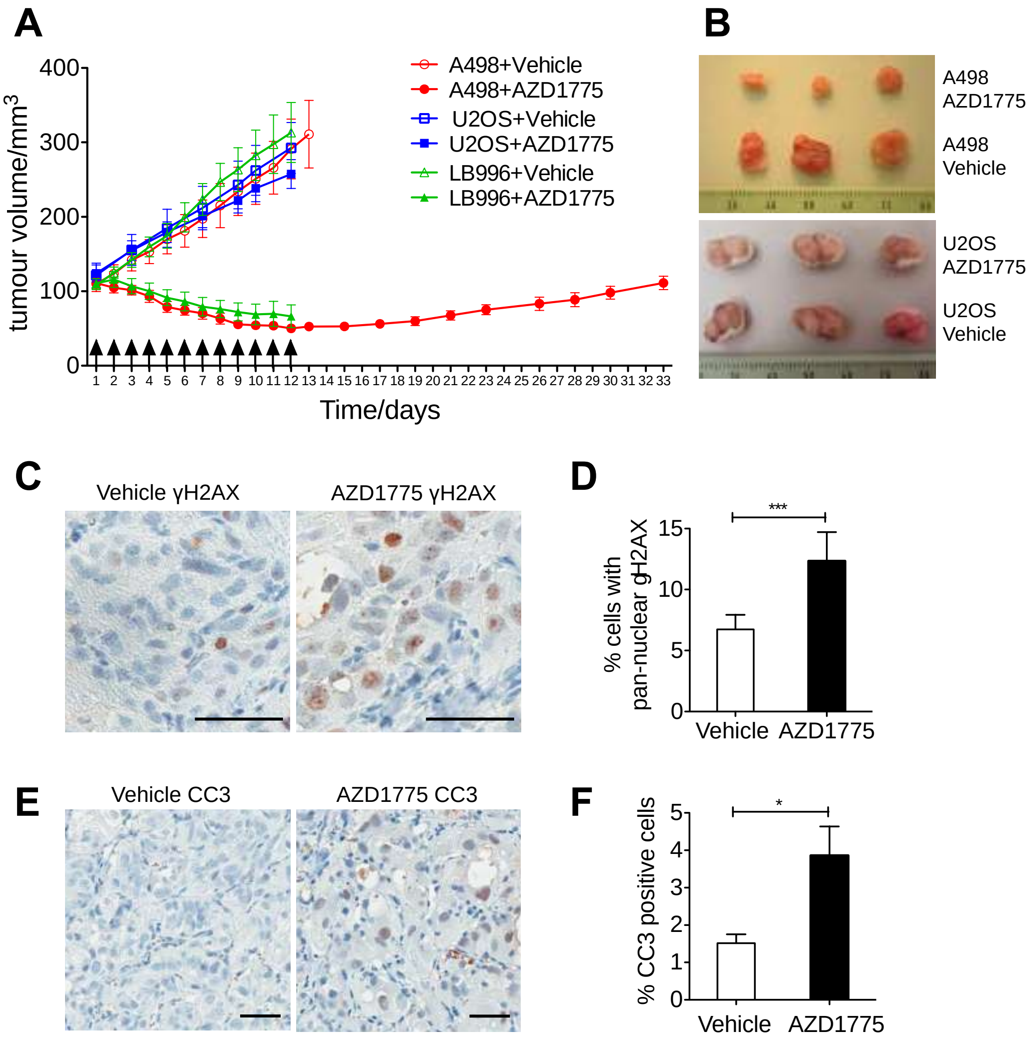

Figure 5. WEE1 inhibitor AZD1775 regressed SETD2-deficient tumor xenografts. (A) Tumor volumes for each treatment group, with arrows indicating the days when the inhibitors were given. A498 and LB996 are SETD2-deficient cell lines while U2OS is SETD2 wild-type. Data are presented as mean ± SEM, n = 7 mice for A498 and U2OS, n = 5 mice for LB996. **(B)** Representative tumors in mice treated with either AZD1775 or vehicle (day 13). **(C–F)** Representative images of tumors generated from A498 cells (C and E) and quantification results (D and F) of immunohistochemistry analysis of pan-nuclear γH2AX (C and D) and cleaved-Caspase-3 (CC3) (E and F) levels in tumors (day 13). Scale bar represents 50 µm. Data are presented as mean ± SEM, n = 3 tumors. ***p < 0.001; *p < 0.05; unpaired and two-tailed t

tests were used.

<u>AZD1775 Pre-Clinical Summary:</u>
AZD1775 is a potent and selective inhibitor of the WEE1 kinase (IC50 = 5.18 nM). In a kinase selectivity screen, among 223 kinases tested, AZD1775 inhibited only 8 kinases (Fgr, Fyn, Lck, Plk3, PTK5, TrkB, and Yes) for more than 80% at 1 μM. Inhibition of CDK1 (CDC2) phosphorylation has been shown to be a dose-dependent on target effect of AZD1775 treatment in cell-based assays, with a half maximal effective concentration (EC50) of 82 nM in a colorectal cell line.

Prior to the xenograft studies shown above **(Figure 5)**, AZD1775 (aka MK1775) has been tested across a variety of tumor types (including lung, glioblastoma, pancreas and ovarian), but very few showed monotherapy activity in unselected genetic background **(Figure 6A-C)** (Bridges et al., 2011; Rajeshkumar et al., 2011; Sarcar et al., 2011), except one study in lung cancer after a 28-day constitutive treatment **(Figure 6D**) (Guertin et al., 2013). The lack of single agent activity in unselected background, as compared with the significant anti-tumor activity in SETD2-deficient tumor types, supports the need for a biomarker-selected clinical study of AZD1775 monotherapy.

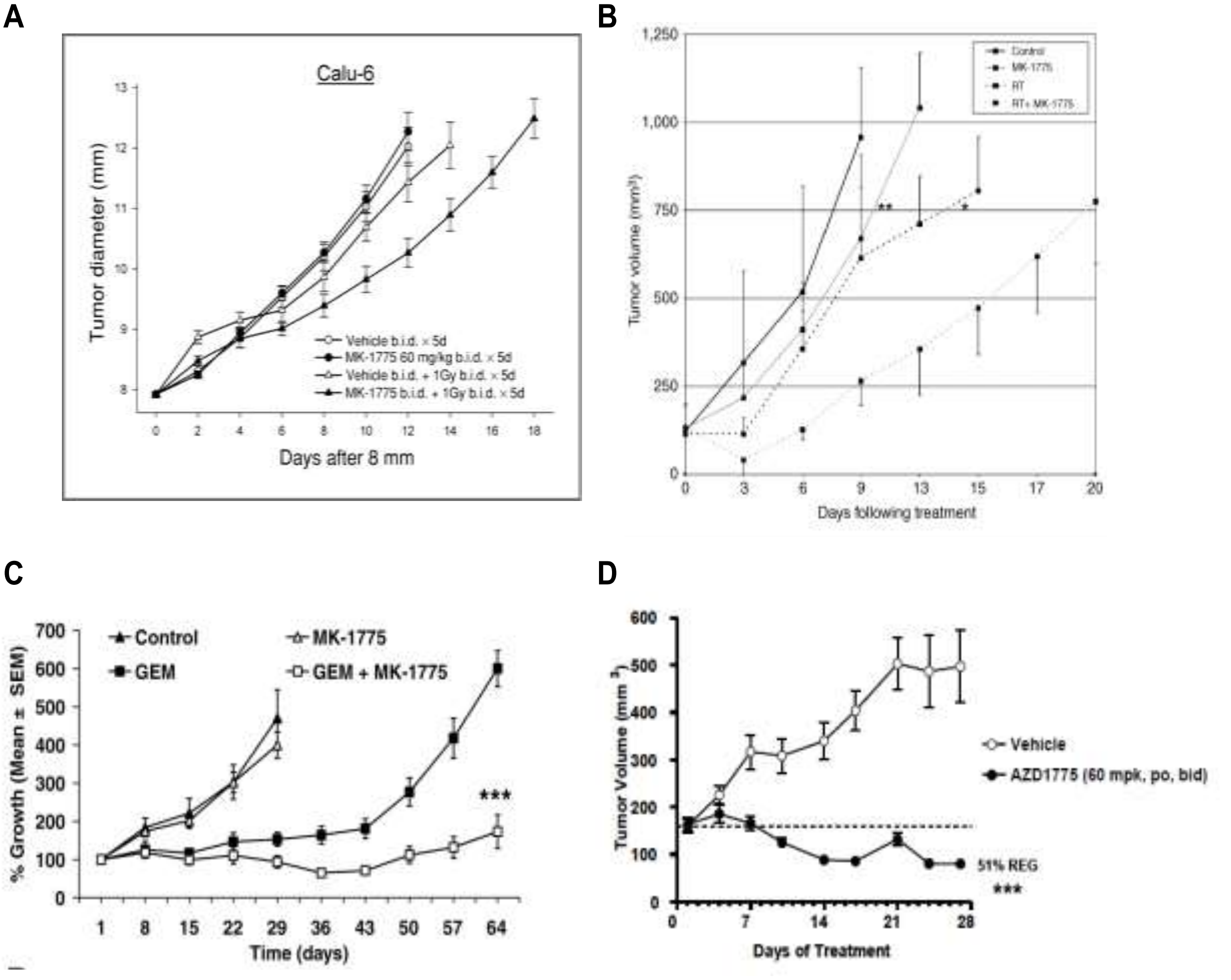

Figure 6. WEE1 inhibitor AZD1775 lack single agent activity in unselected genetic background. (A)

Xenograft tumors made using Calu-6 (human lung cancer cell line) were treated with MK-1775 (AZD1775), 60 mg/kg twice daily (b.i.d.), or radiation, 1 Gy twice daily, or the combination for 5 consecutive days starting when the tumors reached 8 mm in diameter. The error bars are the standard error of the mean tumor diameter for the group and each group contained 8 mice. **(B)** U251 (human glioblastoma cell line) was injected subcutaneously in a mouse flank model. When tumors reached about 150 mm3 in size, mice were randomized into 4 groups: vehicle control, MK-1775 (AZD1775) (60 mg/kg twice daily × 3 days), irradiation (RT) (2 Gy × 3 days), or MK-1775 + irradiation. Each group contained 7 mice. **(C)** PANC374 (patient-derived pancreatic cancer xenograft) was injected subcutaneously in a mouse flank model, when tumors reached a volume of ~200 mm3, mice were randomly assigned to 4 treatment groups: control; MK-1775 (AZD1775) (30 mg/kg. p.o., once daily for 4 weeks; gemcitabine (GEM) (100 mg/kg, i.p., twice weekly on days 1 and 4) for 4 weeks; gemcitabine followed 24 hour later by MK-1775 in the above mentioned dose. **(D)** A427 (lung carcinoma cell line) xenograft–bearing mice were dosed with either vehicle (0.5% methylcellulose) or 60 mg/kg of MK-1775, twice daily for 28 consecutive days. Xenograft tumor volumes were taken twice weekly and plotted (mean volume ± SEM) against days of treatment for vehicle (n = 10) and MK-1775 (n = 10)-treated mice.

2.2 AZD1775

AZD1775 (previously known as MK-1775) is an inhibitor of WEE1, a protein tyrosine kinase. WEE1 phosphorylates and inhibits cyclin-dependent kinases 1 (CDK1) and 2 (CDK2), and is involved in regulation of the intra-S and G2 cell cycle checkpoints. Proper functioning of these checkpoints is essential for DNA metabolism and the DNA damage response (Coleman and Dunphy 1994, Parker and Piwnica-Worms 1992).

CDK1 (also called cell division cycle 2, or CDC2) activity drives a cell from the G2 phase of the cell cycle into mitosis. In response to DNA damage, WEE1 inhibits CDK1 to prevent the cell from dividing until the damaged DNA is repaired (G2 checkpoint arrest). Inhibition of WEE1 is expected to release a tumor cell from DNA damage-induced arrest at the G2/M boundary, so that un-repaired DNA damage may be taken into mitosis (M-phase). Since cancer cells exhibit higher levels of endogenous damage than normal cells, as well as exhibiting loss of one or more DNA damage response (DDR) capabilities, this is expected to preferentially enhance cancer cell death through mitotic catastrophe compared to normal cells.
In vitro experiments demonstrate that AZD1775 has synergistic cytotoxic effects when administered in combination with various DNA damaging agents that have divergent mechanisms of action. Therefore, the clinical development of AZD1775 includes its use as a chemosensitising drug in combination with a cytotoxic agent (or combination of agents) for treatment of advanced solid tumors.

CDK2 activity drives a cell into, and through the DNA synthesis (S-phase) of the cell cycle in which the genome is duplicated in preparation for cell division. An important aspect of CDK2 regulation of replication is the control of replication origin firing. WEE1, under normal circumstances, regulates CDK2 to provide sufficient time to generate the optimum environment for DNA synthesis, such as the generation of a sufficient pool of deoxynucleotide triphosphates (dNTPs), the building blocks of DNA. Inhibition of WEE1 is expected to cause aberrantly high CDK2 activity in S-phase cells that will have multiple consequences for cancer cells that already have G1/S checkpoint aberrations such as p53 mutations or CDKN2A deletions (Sherr 1996). These include de-regulated replication origin firing before sufficient dNTPS are available,

resulting in a higher degree of replication stress (already greater in cancer cells). The resulting increase in levels of replication stress will result in replication fork stalling, the generation of unstable DNA replication structures and ultimately replication fork collapse where endonucleases generate DNA damage. Therefore, it is anticipated that AZD1775 will have independent anti-tumor activity in the absence of added chemotherapy, particularly in cancer cells that already have significantly higher levels of replication stress.

The tumor suppressor protein p53 regulates the G1 checkpoint. As the majority of human cancers harbor abnormalities in this pathway they become more dependent on S- and G2- phase checkpoints (Sherr 1996). Thus, S- and G2-checkpoint abrogation caused by inhibition of WEE1 may selectively sensitize p53-deficient cells to anti-cancer agents (Wang et al 2001) while single-agent activity may be seen in cancers with sufficiently high levels of replication stress and endogenous DNA damage.

In vitro experiments demonstrated that AZD1775 inhibits WEE1 activity and induces DNA damage as well as G2/M checkpoint escape. AZD1775 sensitizes tumor cells to cytotoxic effects of different DNA damaging agents, including chemotherapies. In mouse and rat xenograft models, anti-tumor efficacy of chemotherapy was significantly enhanced by AZD1775. Treatment with AZD1775 in nude mouse xenograft models resulted in significant tumor growth inhibition at tolerated doses, and also enhanced the anti-tumor growth effect of gemcitabine, carboplatin, radiation therapy, and olaparib, a PARP inhibitor that also induces replication associated DNA damage.

Moreover, in preclinical cancer cell models associated with high levels of endogenous replication stress resulting from a combination of G1/S checkpoint deficiencies due to p53 mutations or CDKN2A deletions and the over-expression of oncogenic drivers such as MYC, mutant KRAS or the amplification of Cyclin E, AZD1775 also demonstrated significant single-agent anti-tumor activity.

The early clinical development to date of AZD1775, administered in combination with a "standard of care" cytotoxic agent such as cisplatin, topotecan, carboplatin, or 5-fluouracil (5-FU), focused on the treatment of advanced solid tumors and also the treatment of p53 pathway deficient malignancies.

2.2.1 Clinical experience

AZD1775 has been administered to patients in 12 AstraZeneca-sponsored or Merck-sponsored clinical studies, 6 of which are ongoing. As of 11 November 2016, a total of approximately 551 patients have been exposed to AZD1775 in AstraZeneca-sponsored or Merck-sponsored clinical studies. Of these 551 patients, 103 received AZD1775 monotherapy, 407 patients received AZD1775 in combination with cytotoxic chemotherapy agents and the remaining 41 patients received AZD1775 in combination with targeted therapies MEDI4736 or olaparib. In addition, approximately 350 patients have also received AZD1775 as part of externally-sponsored scientific research. These patients have received single doses per cycle as high as 1300 mg of AZD1775 as monotherapy, 325 mg of AZD1775 in a single-dose in combination with chemotherapy, and 325 mg twice a day (BID) in a multiple-dose regimen in combination with chemotherapy.

The completed or terminated early studies include:

- PN001 (NCT00648648) (except for Part 3): a first-time-in-patients (FTIP), Phase I, dose-escalation study evaluating AZD1775 both as monotherapy and combination therapy with gemcitabine, cisplatin, or carboplatin in adult patients with advanced solid tumors.

- PN004 (NCT01357161): a Phase II study evaluating AZD1775 combined with carboplatin and paclitaxel in patients with platinum-sensitive p53-mutant ovarian cancer

- PN005 (NCT01047007): a Phase I, dose-escalation study evaluating AZD1775 as monotherapy (Part 1), combination therapy with 5-FU (Part 2), and combination therapy with 5 FU plus cisplatin (Part 3) in adult Japanese patients with advanced solid tumors was terminated early due to portfolio prioritization in oncology at Merck after 3 patients had been enrolled in Part 1 and 8 patients had been enrolled in Part 2. Part 3 was not initiated.

- PN008 (NCT01076400): a Phase I/IIa, dose-escalation study evaluating AZD1775 in combination with topotecan plus cisplatin in adult patients with cervical cancer was terminated early due to portfolio prioritization in oncology at Merck after 7 patients had been enrolled in the dose-escalation part of the study. The Phase IIa part was not initiated.

- PN011 (Investigator Sponsored Study): a Phase I study of single-agent AZD1775, in patients with refractory solid tumors, sponsored by the National Cancer Institute (NCI) Cancer Therapy Evaluation Program in collaboration with AstraZeneca and Merck. This study reported AZD1775 monotherapy activity in patients carrying BRCA mutations for the first time.

- D6011C00001 (NCT02087176; SCRI LUN 262): a lead-in Phase II multicenter, randomized, double-blind study comparing AZD1775 plus docetaxel with placebo plus docetaxel in previously treated patients with non-small-cell lung cancer (NSCLC)

- D6011C00002 (NCT02087241; SCRI LUN 261): a Phase II study of AZD1775 plus pemetrexed and carboplatin followed by a randomized comparison of pemetrexed and carboplatin with or without AZD1775 in patients with previously untreated stage IV non-squamous NSCLC

Ongoing:

- D6010C00004 (NCT02272790; SCRI GYN 49): a multicenter Phase II study of AZD1775 plus either paclitaxel, gemcitabine, carboplatin, or pegylated liposomal doxorubicin in patients with platinum-resistant epithelial ovarian, fallopian tube, or primary peritoneal cancer

- D6010C00005 (NCT02511795; SCRI REFMAL 384): a Phase I study evaluating AZD1775 in combination with olaparib in refractory solid tumors.

- D6011C00003 (NCT02341456): a Phase Ib dose-finding study evaluating AZD1775 as

monotherapy and in combination with carboplatin and paclitaxel in adult Asian patients with advanced solid tumors

- D6015C00001 (NCT02482311; SCRI REFMAL 383): a Phase I, dose escalation, safety and pharmacokinetic study of AZD1775 monotherapy (Schedule 1) in patients with advanced or metastatic solid tumors

- D6015C00002 (NCT02617277; SCRI REFMAL 412): a Phase I study assessing the safety, tolerability, and pharmacokinetics of AZD1775 in combination with MEDI4736 in patients with advanced solid tumors

- D6015C00003 (NCT02610075; SCRI REFMAL 398): a Phase Ib study to determine the maximum-tolerated dose (MTD) of AZD1775 monotherapy (Schedule 2) in patients with locally advanced or metastatic solid tumors.

In Study PN001, of 176 evaluable patients who received AZD1775 (either single or multiple doses) as monotherapy or in combination with gemcitabine, cisplatin, or carboplatin, a partial response (PR) (confirmed and unconfirmed) was observed in 17 (9.7%) patients, and stable disease (SD) was observed in 94 (53.4%) patients (AZD1775 Investigator's Brochure[IB]). In Study PN001, 9 patients received AZD1775 monotherapy. Single ascending doses of AZD1775 up to 1300 mg were well tolerated; the maximum tolerated dose (MTD) was not established.

In Study PN004, all patients were treated at the 225 mg AZD1775 BID 2.5-day dose level in combination with paclitaxel and carboplatin. Of the 14 evaluable patients by RECIST v1.1 in Part 1, there were 11 PRs (6 confirmed and 5 unconfirmed), and 3 SDs; 7 patients were evaluable by CA-125 with 3 CRs and 4 PRs. Final data for Part 2 is not available as of the cut-off date for this protocol.

In Study PN005, patients in Part 1 received single-cycle BID dosing of AZD1775 for 5 days at 1 of 2 dose levels as monotherapy. A cohort of 3 patients was enrolled at the starting dose level of AZD1775 65 mg BID and no serious adverse events (SAEs) were experienced. No complete responses (CRs) or PRs were observed in either of Studies PN005 or PN008 at the time that they were terminated.

In Study PN011, patients received single-agent AZD1775 PO BID over 2.5 days per week for 2 weeks in 3-week treatment cycles. Twenty-five patients were enrolled to determine the MTD using a 3+3 design. The MTD was established at 225 mg by mouth (PO) BID for 5 doses on Weeks 1 and 2 of a 3-week schedule. Six patients with BRCA-mutated solid tumors were enrolled at the MTD. Partial responses were confirmed in two of the patients carrying BRCA mutations (ovarian cancer patient and head/neck cancer patient). Paired tumor biopsies were obtained from 5 patients treated at the MTD at baseline and after the 5th AZD1775 dose to determine the levels of pY15-Cdk and γH2AX. The biopsies showed a decrease in pY15-Cdk levels (2/5 paired biopsies). The same biopsies were analyzed for increases in γH2AX, an indicator of DNA damage. Three of the 5 biopsy pairs showed an increase in γH2AX levels. DNA damage response was observed in this study through provided paired tumor biopsies (Do et al 2015).

In Study D6011C00001, 32 patients with NSCLC were treated with 225 mg AZD1775 BID over 2.5 days in combination with docetaxel (75 mg/m2 IV) administered on Day 1 followed by pegfilgrastim on Day 4 of each 21-day cycle. The 3 patients (9.4%) that achieved PR by RECIST v1.1 had TP53 mutations. Twenty-one patients (65.6%) had SD and 10 (47.6%) of these patients had TP53 mutations. The planned Interim Analysis of 32 patients in the single cohort lead-in (Part A) suggested that toxicities associated with AZD1775 given in combination with docetaxel were greater in frequency and severity than with docetaxel alone. Additionally, the analysis revealed that it was very unlikely the target response rate would be reached in this study and a decision was made to terminate enrolment.

In Study D6011C00002, 14 patients with NSCLC were treated with 225 mg AZD1775 BID over 2.5 days in combination with pemetrexed 500 mg/m2 IV and carboplatin AUC 6 IV, both administered on Day 1 of each 21-day cycle. Enrolment was stopped because of the introduction of new therapies for the treatment of first-line NSCLC, such as immunotherapy, which resulted in challenges in patient recruitment. In addition, the planned Interim Analysis of Study D6011C00001 revealed that it was very unlikely that the target response rate would be reached, and increased gastrointestinal and hematologic toxicities associated with AZD1775 were observed.

2.2.2 Pharmacokinetics

The pharmacokinetic (PK) data of AZD1775 following a single oral administration (Study PN001) showed a moderate rate of absorption with a time of maximum concentration (Tmax) occurring at 3 to 4 hours 2 and 4 hrs on day 1 and 3 respectively (IB v 15 p. 119). Post-peak plasma concentrations declined essentially in a mono-exponential manner with a terminal elimination half-life (t1/2) between 9.02 to 12.3 hours 13.2 hours at the 225 mg PMF dose (IB v 15 p. 119). Exposure as measured by maximum plasma drug concentration observed (Cmax) and area under the curve $AUC_{(0-\infty)}$ increased in a dose-proportional manner over the dose range of 325 to 1300 mg. Following single (100 to 325 mg) and multiple dose administrations of AZD1775 (25 to 325 mg BID and 100 to 200 mg once daily [QD]) with carboplatin, cisplatin, and gemcitabine, plasma exposure of AZD1775 was consistent with predictions based on the single-dose regimen. Preliminary investigation of drug-drug interactions (DDIs) in Study PN001 suggest a ~40%increase in the exposure of AZD1775 in the presence of aprepitant (moderate CYP3A4 inhibitor) on both Day 1 and Day 3 of dosing, but no effect with the concomitant administration of steroids (moderate CYP3A4 inducers). Preliminary studies also suggested that the Pre-marketed Oral Formulation of AZD1775 was similar to that of the Fit-For-Purpose formulation.

Based on the preliminary comparison of the results of AZD1775 PK parameters at the 225 mg dose, PK estimates in Asian patients were higher than in Western patients. After single dose administration on Cycle 0 Day 1 (monotherapy), Cmax and AUC at the 225 mg dose were 45% and 35% higher, respectively, in the Asian population as compared to the Western population (Study PN011). At steady state (Cycle 3 Day 1), a similar trend of higher exposure in Asian patients was observed. Additional analysis/investigation will be conducted based on the emerging data to understand the exposure differences between the populations.

2.2.3 Safety

Based on the safety data from the completed AZD1775 clinical studies and preliminary data from

ongoing studies adverse drug reactions to AZD1775 monotherapy include: anemia, neutropenia, thrombocytopenia, QTc prolongation, gastrointestinal events such as dyspepsia, diarrhea, nausea and vomiting (with or without dehydration or serum electrolyte decreases), as well as decreased appetite.

2.2.4 Rationale for the Recommended Phase 2 Dose of AZD1775

Results from the recently reported Phase 1 study of AZD1775 monotherapy in BRCA-deficient solid tumor malignancies evaluating two dosing schedules (BID dosing x 5 doses days 1-3 of each week x 2 weeks; **once** daily dosing days 1-5 each week x 2 weeks, of a 21 day treatment cycle) were used to determine the recommended phase 2 dose and schedule of AZD1775 to be utilized in the current study. In this recently reported phase 1 study, with the **once** daily dosing schedule, doses were escalated from 200 to 400 mg daily. There were two dose-limiting toxicities observed at the 400 mg daily dosing level (both grade 4 pancytopenia), thereby establishing 300 mg daily as the maximally tolerated dose with the **once** daily dosing schedule. With the BID dosing schedule, 225 mg BID x 5 doses days 1-3 for 2 weeks was the previously established recommended phase 2 dose. The frequency of adverse events, in particular related to myelosuppression and fatigue, were similar between the **QD** daily and BID dosing schedules. Anti-tumor activity was observed in the daily dosing schedule with two partial responses and several additional patients with prolonged stable disease. The **once** daily dosing schedule of AZD1775 300 mg on days 1-5, 8-12 of an every 21 day cycle was chosen as the recommended phase 2 dose over BID dosing schedule, given the easier schedule to follow from perspective of patient adherence.

Daily dosing at 300 mg once daily achieves serum concentrations that exceed the target serum concentrations necessary to achieve a synthetic lethal interaction in SETD2-deficient pre-clinical models. The IC_{50} for synthetic lethal effect of AZD1775 in SETD2-deficient renal cell carcinoma cell lines (A498 and LB996) was 87 nM and 68 nM, respectively. As shown in the PK plot below, a single daily dose of escalating from 100 to 325 mg daily of AZD1775 (in combination with chemotherapy which does not affect concentration levels) achieves serum concentrations exceeeding the IC_{50} for the majority of the 24-hour dosing period.

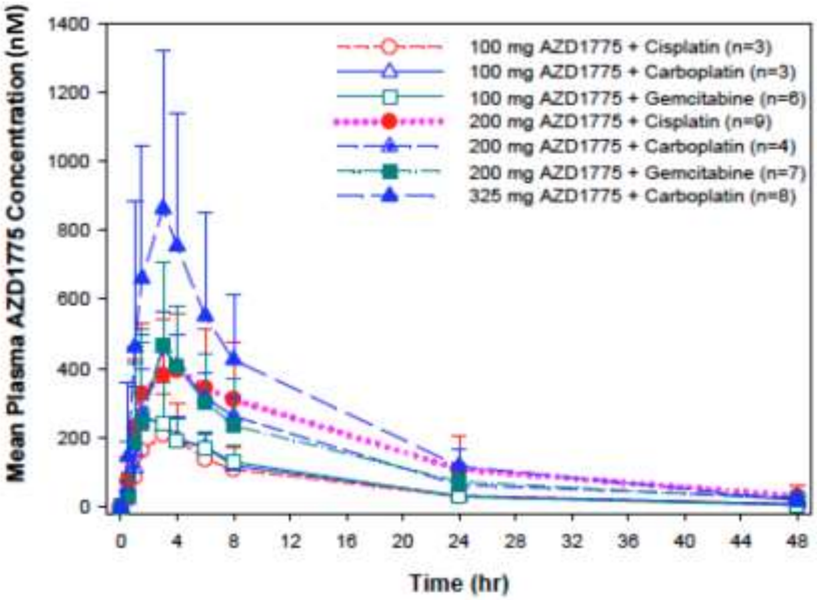

2.3 Rationale

Rationale for Study Design

The rationale to include any solid tumor with pathogenic loss-of-function SETD2 mutation, rather than restrict to particular tumor type(s), is as follows: (1) pre-clinical evidence of activity across multiple tumor types including small cell lung and renal cell carcinoma, (2) presence of inactivating SETD2 mutations at an appreciable frequency across multiple tumor types (Figure 1), and (3) the mechanistic underpinnings of the synthetic lethal interaction are independent of histologic subtype of tumor.

Clear cell renal cell carcinoma and other solid tumor types will be evaluated as two independent cohorts to allow for the adequate assessment of efficacy in each treatment group. The prevalence of *SETD2* mutations in clear cell RCC approaches 10-15%, and almost always accompanied by concomitant loss of chromosome 3p resulting in bi-allelic loss of function.

Though the clinical development of AZD1775 is predominantly in combination with DNA damaging agents, including cytotoxic chemotherapy and PARP inhibitors, we propose to evaluate the single agent activity given the significant and durable tumor regressions observed with monotherapy in SETD2-deficient xenograft models, as well as the potential for significant and overlapping toxicity with combination studies involving AZD1775 that may limit the ability to maintain dose intensity in patients with responding tumors.

Feasibility of Accrual

As shown in Figure 1, loss-of-function SETD2 mutations occur at appreciable frequencies across multiple advanced solid tumor types, including renal cell carcinoma, pancreatic NET, pancreatic and other GI adenocarcinomas. When summed together, SETD2-deficient tumors comprise a appreciable subset of advanced solid tumor malignancies for which targeted treatment options are limited. Furthermore, SETD2 is commonly part of CLIA-certified sequencing panels including

the UCSF500 gene panel, Foundation One, and other commercial assays. SETD2 mutations are not currently part of the NCI-MATCH study and therefore will not compete with accrual to this study.

We have conducted a pilot study analyzing the frequency of renal cell carcinoma patients seen at UCSF who have undergone Foundation One testing. Over a three month period, 25 renal cell carcinoma patients have been identified who have Foundation One test results available. Of these 25 patients, 5 (20%) have had pathogenic SETD2 mutations. Over 90% possessed concomitant loss of chromosome 3p, representative of biallelic loss of SETD2. This is expected to translate into ~ 1.5 renal cell carcinoma patients/month with SETD2 mutations detected at UCSF.

With respect to frequency of SETD2 alterations in other tumor types, we have detected SETD2 mutations in 18 cases tested with UCSF500 NGS panel over an approximately 1.5 year time period. Tumor types included neuroendocrine tumors (N = 4), lung cancer (N = 2), among other tumor types. Among the broader list of ET-CTN sites, Memorial Sloan Kettering has recently presented an analysis of 39 patients with neuroendocrine tumors screened using their MSK-IMPACT CLIA-certified panel (Raj et al. J Clin Oncol 34, 2016 (suppl 4S; abstr 246)) demonstrating that 7 patients (18%) had pathogenic SETD2 mutations.

2.4 Correlative Studies Background

We propose to evaluate the loss of H3K36me3 mark as an integrated biomarker to evaluate its predictive utility of response to WEE1 inhibition. Based on our pre-clinical data presented above, we hypothesize that loss of expression will be positively associated with likelihood of tumor response and clinical benefit. If the current study supports this hypothesis, this would form the strong rationale to support CLIA validation of the immunohistochemial assay and prospective evaluation as an integral biomarker used for treatment selection in subsequent clinical studies, and ultimately, may help to select patients for this therapy if FDA approved on the basis of subsequent clinical studies.

The rationale for evaluating loss of H3K36me3 mark, as opposed to solely relying on detection of somatic loss-of-function SETD2 mutations, as a predictive biomarker for WEE1 inhibition, stems from the fact that there are multiple converging mutations, in addition to SETD2, which may ultimately lead to loss of H3K36me3 mark and subsequent synthetic lethality with WEE1 inhibition. This includes overexpression of KDM4A and KDM4B, as well as H3.3 mutations (G34R/V, K36M), which occur mutually exclusively to SETD2 mutations and are highly prevalent in certain tumor types **(Figure 7A)**. For example, KDM4A overexpression occurs in 12% of bladder and ovarian cancers, and H3.3K36M mutations occur in 90% of chondroblastomas.

Moreover, the authors have shown that, cancer cells with KDM4A overexpression, as well as H3.3K36M mutation are hypersensitive to WEE1 inhibition by AZD1775 **(Figure 7B)**. Therefore, directly assessing for loss of H3K36me3 mark, rather than selecting for specific mutations in a subset of genes, may ultimately capture a broader array of tumors that would have a synthetic lethal effect with WEE1 inhibition.

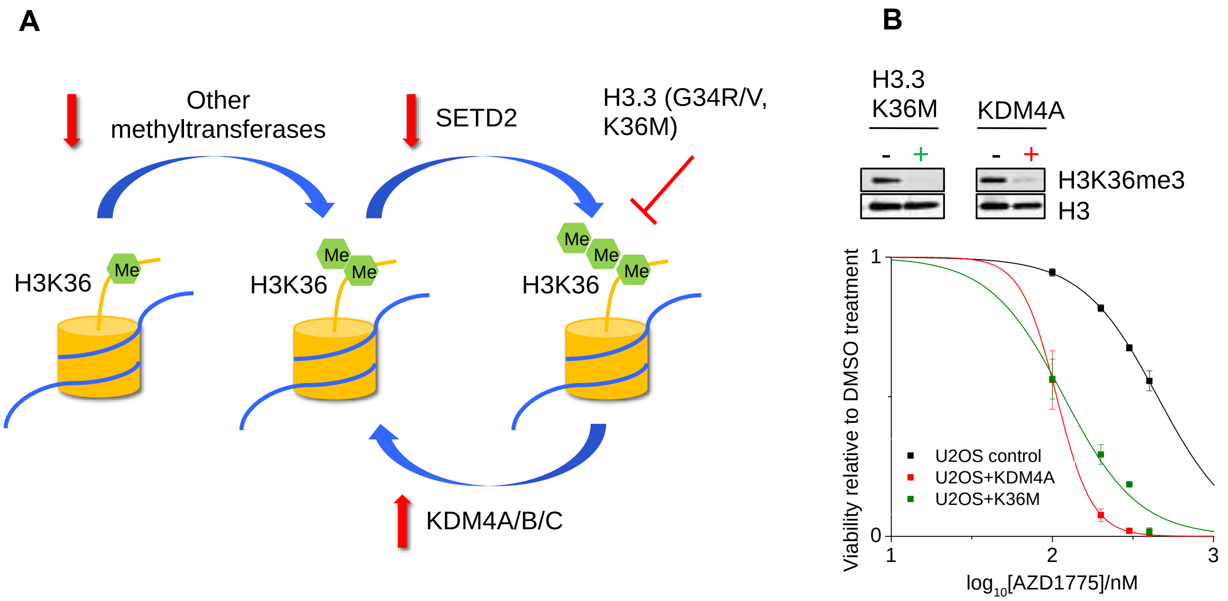

Figure 7. H3K36me3-deficient cancers are hypersensitive to WEE1 inhibition. (A) Multiple mutations can cause loss of H3K36me3: loss of SETD2 (the sole methyltransferase for H3K36me3), overexpression of the oncogene KDM4A (which demethylates H3K36me3), or mutation of histone H3.3 (G34V/R or K36M). **(B)** Western blot analysis of H3K36me3 levels in SETD2 wild-type U2OS cells stably expressing H3.3K36M or KDM4A. Viability curves of U2OS cells expressing either an empty vector, H3.3K36M, or KDM4A after exposure to AZD1775 (5 days).

3. PATIENT SELECTION

3.1 Inclusion Criteria

3.1.1 Cohort A: Histologically confirmed locally advanced or metastatic solid tumor malignancy other than clear cell renal cell carcinoma with progression on at least one prior systemic therapy and presence of pathogenic *SETD2* mutation detected in tumor tissue detected using a CLIA-certified next generation sequencing panel (e.g. UCSF500, FoundationOne).

3.1.2 Cohort B: Patients with histologically confirmed locally advanced or metastatic clear cell renal cell caricnoma (with clear cell component on pathology), who have been treated with at least one prior systemic therapy for locally advanced or metastatic disease, including either tyrosine kinase inhibitor and/or immune checkpoint inhibitor, with evidence of pathogenic *SETD2* mutation on CLIA-certified next generation sequencing panel.

All NGS sequencing reports will be reviewed by the UCSF Molecular Tumor Board to verify pathogenicity of *SETD2* mutation.

UCSF Molecular Tumor Board contact information:

Gregor Krings MD
Molecular Pathologist, UCSF Molecular Tumor Board
San Francisco, CA 94143
Gregor.Krings@ucsf.edu

Each NGS report will be redacted for PHI prior to submission from investigational site to UCSF MTB.

3.1.3 Measurable disease by RECIST 1.1 criteria.

3.1.4 Age ≥18 years. Because no dosing or adverse event data are currently available on the use of AZD1775 in patients <18 years of age, children are excluded from this study, but will be eligible for future pediatric trials.

3.1.5 ECOG performance status ≤ 1 (Karnofsky ≥ 70%, see Appendix A).

3.1.6 Patients must have normal organ and marrow function as defined below:

- Absolute neutrophil count ≥1,500/mcL
- Platelets ≥100,000/mcL
- Hemoglobin ≥ 9 g/dL
- Total bilirubin within normal institutional limits (WLN) or ≤ 1.5 x upper limit of normal (ULN) in patients with liver metastases; or total bilirubin ≤ 3 x ULN with direct bilirubin WLN in patients with well documented Gilbert's syndrome
- AST(SGOT)/ALT(SGPT) ≤ 3.0 × institutional upper limit of normal (≤ 5 x ULN if known liver metastases)
- Serum creatinine ≤ 1.5 x UNL,
  OR
- Creatinine clearance ≥ 45 ml/min (24 hour urine creatinine clearance or calculated by Cockroft-Gualt equation)

3.1.7 Any prior radiation must have been completed at least 7 days prior to the start of study drugs, and patients must have recovered from any acute adverse effects prior to the start of study treatment.

3.1.8 Female patients who are not of child-bearing potential and fertile females of childbearing potential who agree to use adequate contraceptive measures from 2 weeks prior to the study and until 1 month after study treatment discontinuation, who are not breastfeeding, and who have a negative serum or urine pregnancy test within 3 days prior to the start of study treatment.

3.1.9 Male patients willing to abstain or use barrier contraception (i.e. condoms) for the duration of the study and for 3 months after treatment stops.

3.1.10 Willingness and ability to comply with study and follow-up procedures.

3.1.11 Has read and understands the informed consent form and has given written informed consent prior to any study procedures.

3.2 Exclusion Criteria

3.2.1 Use of anti-cancer treatment drug ≤ 21 days or 5 half-lives (whichever is shorter) prior to the first dose of AZD1775. For drugs for which 5 half-lives is ≤ 21 days, a minimum of 10 days between termination of the prior treatment and administration of AZD1775 treatment is required.

3.2.2 Previous radiation therapy completed ≤7 days prior to the start of study drugs.

3.2.3 Major surgical procedures ≤28 days of beginning study treatment, or minor surgical procedures ≤7 days. No waiting period required following port-a-cath or other central venous access placement.

3.2.4 Grade >1 toxicity from prior therapy (except alopecia or anorexia).

3.2.5 Patient has an inability to swallow oral medications. Note: Patient may not have a percutaneous endoscopic gastrostomy (PEG) tube or be receiving total parenteral nutrition (TPN).

3.2.6 No other anticancer-therapy (chemotherapy, immunotherapy, hormonal anti-cancer therapy, radiotherapy [except for palliative local radiotherapy]), biological therapy or other novel agent is to be permitted while the patient is receiving study medication. Patients on LHRH analogue treatment for more than 6 months are allowed entry into the study and may continue at the discretion of the Investigator.

3.2.7 Known malignant central nervous system (CNS) disease other than neurologically stable, treated brain metastases – defined as metastasis having no evidence of progression or hemorrhage for at least 2 weeks after treatment. Must be off any systemic corticosteroids for the treatment of brain metastases for at least 14 days prior to enrolment.

Patients with known active brain metastases should be excluded from this clinical trial because of their poor prognosis and because they often develop progressive neurologic dysfunction that would confound the evaluation of neurologic and other adverse events.

3.2.8 Any known hypersensitivity or contraindication to the components of the study drug AZD1775.

3.2.9 Patient has had prescription or non-prescription drugs or other products known to be sensitive to CYP3A4 substrates or CYP3A4 substrates with a narrow therapeutic index, or to be moderate to strong inhibitors/inducers of CYP3A4 which cannot be discontinued 2 weeks prior to Day 1 of dosing and withheld throughout the study until 2 weeks after the last dose of study drug. Co-administration of aprepitant or fosaprepitant during this study is prohibited. The use of sensitive substrates of CYP3A4, such as atorvastatin, simvastatin and lovastatin, is also prohibited in this study. (see Appendix B).

Transporter studies (in vitro) have shown that AZD1775 is an inhibitor of breast cancer resistance protein (BCRP). Please refer to Appendix B for use with BCRP substrates.

Herbal preparations are not allowed throughout the study. These herbal medications include but are not limited to: St. John's wort, kava, ephedra (ma hung), gingko biloba, dehydroepiandrosterone (DHEA), yohimbe, saw palmetto and ginseng. Patients should stop using these herbal medications 7 days prior to first dose of study treatment.

3.2.10 Any of the following cardiac diseases currently or within the last 6 months as defined by New York Heart Association (NYHA) ≥ Class 2.

- Unstable angina pectoris
- Congestive heart failure
- Acute myocardial infarction
- Conduction abnormality not controlled with pacemaker or medication

Significant ventricular or supraventricular arrhythmias (patients with chronic rate-controlled atrial fibrillation in the absence of other cardiac abnormalities are eligible)

- AZD1775 should not be given to patients who have a history of Torsades de pointes unless all risk factors that contributed to Torsades have been corrected. AZD1775 has not been studied in patients with ventricular arrhythmias or recent myocardial infarction.

3.2.11 Mean resting QTc interval using the Fridericia formula (QTcF) > 450 ms (ie, grade 1 or higher) for males and > 470 ms for females on ECG prior to initiation of study treatment obtained from 3 electrocardiograms (ECGs) obtained 2-5 minutes apart at study entry, or history of congential long QT syndrome.

If baseline QTc on screening ECG is > 450 ms for males or > 470 ms for females:

Check potassium and magnesium serum levels
Correct any identified hypokalemia and/or hypomagnesemia and repeat ECG to confirm QTcF interval

For patients with baseline heart rate (HR) < 60 bpm or > 100 bpm, manual measurement of QT interval by cardiologist is required, with Fridericia correction applied to that manual measurement to determine the QTc for eligibility consideration.

Note: For patients with HR 60-100 bpm, manual measurement of QTc interval is NOT required.

3.2.12 Uncontrolled intercurrent illness including, but not limited to, ongoing or active infection, or psychiatric illness/social situations that would limit compliance with study requirements.

3.2.13 Pregnant or breastfeeding women.

Pregnant women are excluded from this study because AZD1775 is WEE1 inhibitor with the potential for teratogenic or abortifacient effects. Because there is an unknown but potential risk for adverse events in nursing infants secondary to treatment of the mother with AZD1775, breastfeeding should be discontinued if the mother is treated with AZD1775.

3.2.14 Prior treatment with WEE1 inhibitor.

3.3 Inclusion of Women and Minorities

NIH policy requires that women and members of minority groups and their subpopulations be included in all NIH-supported biomedical and behavioral research projects involving NIH-defined clinical research unless a clear and compelling rationale and justification establishes to the satisfaction of the funding Institute & Center (IC) Director that inclusion is inappropriate with respect to the health of the subjects or the purpose of the research. Exclusion under other circumstances must be designated by the Director, NIH, upon the recommendation of an IC Director based on a compelling rationale and justification. Cost is not an acceptable reason for

exclusion except when the study would duplicate data from other sources. Women of childbearing potential should not be routinely excluded from participation in clinical research. Please see http://grants.nih.gov/grants/funding/phs398/phs398.pdf.

Planned Enrollment Table

**Racial Categories**	**Ethnic Categories**				**Total**
	**Not Hispanic or Latino**		**Hispanic or Latino**		
	**Female**	**Male**	**Female**	**Male**	
American Indian/ Alaska Native	0	0	0	0	**0**
Asian	3	2	0	0	**5**
Native Hawaiian or Other Pacific Islander	0	1	0	0	**1**
Black or African American	2	2	0	0	**4**
White	7	7	2	2	**18**
More Than One Race	1	1	0	0	**2**
**Total**	**13**	**13**	**2**	**2**	**30**

4. REGISTRATION PROCEDURES

4.1 Investigator and Research Associate Registration with CTEP

Food and Drug Administration (FDA) regulations and National Cancer Institute (NCI) policy require all individuals contributing to NCI-sponsored trials to register and to renew their registration annually. To register, all individuals must obtain a Cancer Therapy Evaluation Program (CTEP) Identity and Access Management (IAM) account (https://ctepcore.nci.nih.gov/iam). In addition, persons with a registration type of Investigator (IVR), Non-Physician Investigator (NPIVR), or Associate Plus (AP) (i.e., clinical site staff requiring write access to OPEN or RAVE or acting as a primary site contact) must complete their annual registration using CTEP's web-based Registration and Credential Repository (RCR) (https://ctepcore.nci.nih.gov/rcr). Documentation requirements per registration type are outlined in the table below.

**Documentation Required**	**IVR**	**NPIVR**	**AP**	**A**
FDA Form 1572	✔	✔		
Financial Disclosure Form	✔	✔	✔	
NCI Biosketch (education, training, employment, license, and certification)	✔	✔	✔	
HSP/GCP training	✔	✔	✔	
Agent Shipment Form (if applicable)	✔			
CV (optional)	✔	✔	✔	

An active CTEP-IAM user account and appropriate RCR registration is required to access all CTEP and CTSU (Cancer Trials Support Unit) websites and applications. In addition, IVRs and NPIVRs must list all clinical practice sites and IRBs covering their practice sites on the FDA Form 1572 in RCR to allow the following:

- Added to a site roster
- Assigned the treating, credit, consenting, or drug shipment (IVR only) tasks in OPEN
- Act as the site-protocol PI on the IRB approval
- Assigned the Clinical Investigator (CI) role on the Delegation of Tasks Log (DTL).

Additional information can be found on the CTEP website at < https://ctep.cancer.gov/investigatorResources/default.htm >. For questions, please contact the RCR ***Help Desk*** by email at < RCRHelpDesk@nih.gov >.

4.2 Site Registration

This study is supported by the NCI Cancer Trials Support Unit (CTSU).

Each investigator or group of investigators at a clinical site must obtain IRB approval for this protocol and submit IRB approval and supporting documentation to the CTSU Regulatory Office before they can be approved to enroll patients. Assignment of site registration status in the CTSU Regulatory Support System (RSS) uses extensive data to make a determination of whether a site has fulfilled all regulatory criteria including but not limited to the following:

- An active Federal Wide Assurance (FWA) number
- An active roster affiliation with the Lead Network or a participating organization
- A valid IRB approval
- Compliance with all protocol specific requirements

In addition, the site-protocol Principal Investigator (PI) must meet the following criteria:

- Active registration status
- The IRB number of the site IRB of record listed on their Form FDA 1572
- An active status on a participating roster at the registering site

Sites participating on the NCI CIRB initiative that are approved by the CIRB for this study are not required to submit IRB approval documentation to the CTSU Regulatory Office. For sites using the CIRB, IRB approval information is received from the CIRB and applied to the RSS in an automated process. Signatory Institutions must submit a Study Specific Worksheet for Local Context (SSW) to the CIRB via IRBManager to indicate their intent to open the study locally. The CIRB's approval of the SSW is then communicated to the CTSU Regulatory Office. In order for the SSW approval to be processed, the Signatory Institution must inform the CTSU which CIRB-approved institutions aligned with the Signatory Institution are participating in the study.

4.2.1 Downloading Regulatory Documents

Site registration forms may be downloaded from the 10170 protocol page located on the CTSU Web site. Permission to view and download this protocol is restricted and is based on person and site roster data housed in the CTSU RSS. To participate, Investigators and Associates must be associated with the Corresponding or Participating protocol organization in the RSS.

- Go to https://www.ctsu.org and log in using your CTEP-IAM username and password.
- Click on the Protocols tab in the upper left of your screen.
- Either enter the protocol # in the search field at the top of the protocol tree, or
- Click on the By Lead Organization folder to expand, then select LAO-CT018, and protocol #10170.
- Click on LPO Documents, select the Site Registration documents link, and download and complete the forms provided. (Note: For sites under the CIRB initiative, IRB data will load to RSS as described above.)

4.2.2 Requirements For 10170 Site Registration:

- IRB approval (For sites not participating via the NCI CIRB; local IRB documentation, an IRB-signed CTSU IRB Certification Form, Protocol of Human Subjects Assurance Identification/IRB Certification/Declaration of Exemption Form, or combination is accepted)

4.2.3 Submitting Regulatory Documents

Submit required forms and documents to the CTSU Regulatory Office, where they will be entered and tracked in the CTSU RSS.

Regulatory Submission Portal: www.ctsu.org (members' area) → Regulatory Tab

→Regulatory Submission
When applicable, original documents should be mailed to:
CTSU Regulatory Office
1818 Market Street, Suite 3000
Philadelphia, PA 19103

Institutions with patients waiting that are unable to use the Portal should alert the CTSU Regulatory Office immediately at 1-866-651-2878 in order to receive further instruction and support.

4.2.4 Checking **Site** Registration Status

You can verify your site registration status on the members' section of the CTSU website.

- Go to https://www.ctsu.org and log in to the members' area using your CTEP-IAM username and password
- Click on the Regulatory tab at the top of your screen
- Click on the Site Registration tab
- Enter your 5-character CTEP Institution Code and click on Go

Note: The status given only reflects compliance with IRB documentation and institutional compliance with protocol-specific requirements as outlined by the Lead Network. It does not reflect compliance with protocol requirements for individuals participating on the protocol or the enrolling investigator's status with the NCI or their affiliated networks.

4.3 Patient Registration

4.3.1 OPEN / IWRS

Patient enrollment will be facilitated using the Oncology Patient Enrollment Network (OPEN). OPEN is a web-based registration system available to users on a 24/7 basis. It is integrated with the CTSU Enterprise System for regulatory and roster data interchange and with the Theradex Interactive Web Response System (IWRS) for retrieval of patient registration/randomization assignment. Patient enrollment data entered by Registrars in OPEN / IWRS will automatically transfer to the NCI's clinical data management system, Medidata Rave.

The OPEN system will provide the site with a printable confirmation of registration and treatment information. Please print this confirmation for your records.

4.3.2 OPEN/IWRS User Requirements

OPEN/IWRS users must meet the following requirements:

- Have a valid CTEP-IAM account (*i.e.*, CTEP username and password).
- To enroll patients or request slot reservations: Be on an ETCTN Corresponding or Participating Organization roster with the role of Registrar. Registrars must hold a minimum of an AP registration type. If a DTL is required for the study, the registrar(s) must also be assigned the OPEN Registrar task on the DTL.
- To approve slot reservations or access cohort management: Be identified to Theradex as the "Client Admin" for the study.
- Have regulatory approval for the conduct of the study at their site.

Prior to accessing OPEN/IWRS, site staff should verify the following:

- All eligibility criteria have been met within the protocol stated timeframes.
- If applicable, all patients have signed an appropriate consent form and HIPAA authorization form.

4.3.3 OPEN/IWRS Questions?

Further instructional information on OPEN is provided on the OPEN tab of the CTSU website at https://www.ctsu.org or at https://open.ctsu.org. For any additional questions contact the CTSU Help Desk at 1-888-823-5923 or ctsucontact@westat.com.

4.4 General Guidelines

Following registration, patients should begin protocol treatment within 7 days. Issues that would cause treatment delays should be discussed with the Principal Investigator. If a patient does not receive protocol therapy following registration, the patient's registration on the study may be canceled. The Study Coordinator should be notified of cancellations as soon as possible.

Except in very unusual circumstances, each participating institution will order DCTD-supplied agents directly from CTEP. Agents may be ordered by a participating site only after the initial IRB approval for the site has been forwarded by the Coordinating Center to the CTEP PIO (PIO@ctep.nci.nih.gov) except for Group studies.

5. TREATMENT PLAN

5.1 AZD1775 Administration

Treatment will be administered on an outpatient basis. Reported adverse events and potential risks are described in Section 7. Appropriate dose modifications are described in Section 6. No investigational or commercial agents or therapies other than those described below may be administered with the intent to treat the patient's malignancy.

- AZD1775 should be taken orally once daily on days 1-5, 8-12 of an every 21 day cycle, at a starting dose of 300 mg with 8 ounces of water approximately 2 hours before or 2 hours

after food.

- If a patient misses the daily dose according to the schedule, the dose should be taken as soon as possible, but not more than 12 hours after the missed dose was scheduled. If greater than 12 hours, the missed dose should be skipped and the patient should take the next dose when scheduled.

- If vomiting occurs after a patient takes the AZD1775 dose, the patient should be instructed not to retake the dose, but to wait until the next scheduled dose of AZD1775. If no dose is scheduled for the following day, the dose will not be 'made up'. If vomiting persists, the patient should contact the Investigator.

- Women of childbearing potential (WoCBP) may be included only if acceptable contraception is in place for two weeks before study entry, for the duration of the treatment with the study drug and for 1 month after the last dose of AZD1775.

- WoCBP defined as: Women between menarche and menopause who have not been permanently or surgically sterilized and are capable of procreation.

- All WoCBP must have a negative pregnancy test within 3 days prior to study entry and prior to starting each treatment cycle.

- Male patients who are involved in the study must agree to avoid procreative and unprotected sex and must not donate sperm during the study and for 3 months after the last dose of AZD1775. Where the female partner is pregnant or not using effective birth control, men should be advised to abstain while in the study and for 3 months after the last dose of AZD1775.

- Female partners, who are of child-bearing potential, of men participating in clinical studies of AZD1775 will also be required to use effective contraceptive measures while their partner is on study drug and for 3 months thereafter.

- Male patients will be advised to arrange for the freezing of sperm samples prior to the start of the study should they wish to father children while on AZD1775 or during the 3 months after stopping AZD1775.

The patient will be requested to maintain a medication diary of each dose of medication. The medication diary will be returned to clinic staff at the end of each course (see Appendix C).

AZD1775 provided for this study will be used only as directed in the study protocol.

The study personnel will account for all study drugs dispensed to and returned from the patient.

Patients must return all unused medication and empty containers to the Investigator.

The study personnel at the investigational site will account for all drugs dispensed and returned and for appropriate destruction. Certificates of delivery, destruction and return must be signed.

5.2 General Concomitant Medication and Supportive Care Guidelines

All concomitant medications received within 14 days before the first dose of study medication and 30 days after the last dose of study medication should be recorded. Concomitant medications must be recorded in the appropriate sections of the CRF.

Mandatory prophylactic anti-emetics will be required for all patients. Treatment with the anti-emetics aprepitant [Emend] and fosaprepitant are excluded because of known drug-drug interactions.

In addition, loperamide (Imodium) is required at the first onset of diarrhea according to ASCO guidelines. Oral loperamide (Imodium) 2 mg should be administered every 2 hours until diarrhea-free for at least 12 hours (not to exceed 16 mg per day).

Medications may be administered for maintenance of existing conditions prior to study enrollment or for a new condition that develops while on study, including but not limited to the following:

- Bisphosphonates and receptor activator of nuclear factor kappa-B ligand (RANKL) inhibitors (e.g. denosumab).
- Patients requiring therapeutic warfarin or coumarin-derivative anticoagulants will be monitored with international normalized ratio (INR) and prothrombin time (PT) as clinically indicated.
- Low molecular weight heparin (LMWH), rivaroxaban, or equivalent anticoagulant therapy is permitted where clinically indicated.

Patients may receive treatment with megestrol acetate when prescribed for appetite stimulation.

Medication other than that described above, which is considered necessary for the patient's safety and wellbeing, may be given at the discretion of the Investigator and recorded in the appropriate sections of the CRF.

Patients may receive palliative radiotherapy during the trial only for local pain control, and only if in the opinion of the treating Investigator the patient does not have disease progression. The radiation field cannot encompass the sole target lesion. Radiation to the sole target lesion is considered disease progression and the patient should be removed from study treatment.

5.3 Prohibited concomitant mediations

Because there is a potential for interaction of AZD1775 with other concomitantly administered drugs, the case report form must capture the concurrent use of all other drugs, over-the-counter medications, or alternative therapies. The Principal Investigator should be alerted if the patient is taking any agent known to affect or with the potential for drug interactions. The study team

should check a frequently-updated medical reference for a list of drugs to avoid or minimize use of. Appendix C (Patient Drug Information Handout and Wallet Card) should be provided to patients if available.

The following treatments and the medications listed in Appendix B are prohibited or used with caution while in this study. Any further questions regarding concomitant treatments should be referred to the Principal Investigator:

- No formal clinical drug interaction studies have been performed with AZD1775. An exploratory assessment of the effect of aprepitant on AZD1775 exposure in oncology patients suggests that there is a drug interaction between AZD1775 and aprepitant, as exposure to AZD1775 increased by ~40% when aprepitant was co-administered with AZD1775. The observed increase in AZD1775 exposure is likely the result of CYP3A4 inhibition by aprepitant. This increase in exposure is statistically significant. At the selected MTDs, this increase may also be of clinical importance. Therefore, concomitant treatment with aprepitant and fosaprepitant is not allowable per protocol until further evaluation.

- Potent or moderate inhibitors or inducers of CYP3A4, sensitive CYP3A4 substrates, and CYP3A4 substrates with a narrow therapeutic window should be avoided until additional data on drug-drug interactions (DDI) becomes available. The use of sensitive substrates of CYP3A4, such as atorvastatin, simvastatin and lovastatin, is prohibited in this study. As grapefruit and Seville oranges are known to contain moderate inhibitors of CYP3A4, these fruits or their products (including marmalade, juice, etc.) should be avoided while taking AZD1775.

- In vitro data suggests that AZD1775 may also be a weak reversible inhibitor of CYP2C19. Caution should be exercised with concomitant administration of AZD1775 and agents that are sensitive substrates of CYP2C19, or substrates of this enzyme with narrow therapeutic range; refer to Appendix B for a list of sensitive substrates of CYP2C19, or substrates of this enzyme with narrow therapeutic range.

- AZD1775 has been shown to be a weak inducer of CYP1A2 in vitro with a maximum measured response between donors of 39.9% to 93.1% (at 10 µM) and 18.6% to 32.5% (at 5 µM) of the positive control omeprazole (50 µM). Given the nature of the AZD1775 dosing schedule, however, the risk of induction in the clinic is considered low. No specific precautions are recommended at this time, except to be initially vigilant when using substrates of CYP1A2 with a narrow therapeutic range.

- In vitro studies have shown that AZD1775 may be a substrate and inhibitor for human P-glycoprotein (P-gp). Caution should be exercised when agents that are inhibitors or substrates of P-gp are administered concomitantly with AZD1775 (see Appendix B).

- *In vitro* transporter studies have shown AZD1775 to be an inhibitor of BCRP (IC50 5.1 µM). This finding is particularly relevant for drugs administered orally where exposure is normally limited by BCRP-mediated efflux, in particular some statins, such as

rosuvastatin. Other drugs where the disposition is mediated via BCRP should be administered with caution, dose modification considered or substituted by an alternative drug.

- Metformin should be used with caution. AZD1775 has been shown to be an inhibitor of MATE1 and MATE2K transporters. A drug interaction with substrates of either transporter cannot be ruled out, the most important substrate known to date being metformin. Herbal preparations/medications are not allowed throughout the study. These herbal medications include, but are not limited to: St. John's wort, kava, ephedra (ma huang), gingko biloba, dehydroepiandrosterone (DHEA), yohimbe, saw palmetto, and ginseng. Patients should stop using these herbal medications 7 days prior to first dose of AZD1775.

5.4 Duration of Therapy

In the absence of treatment delays due to adverse event(s), treatment may continue until one of the following criteria applies:

- Disease progression by RECIST 1.1 criteria
- Intercurrent illness that prevents further administration of treatment
- Unacceptable adverse event(s)
- Patient decides to withdraw from the study
- General or specific changes in the patient's condition render the patient unacceptable for further treatment in the judgment of the investigator
- Clinical progression
- Patient non-compliance
- Pregnancy
    - All women of child bearing potential should be instructed to contact the investigator immediately if they suspect they might be pregnant (e.g., missed or late menstrual period) at any time during study participation.
    - The investigator must immediately notify CTEP in the event of a confirmed pregnancy in a patient participating in the study.
- Termination of the study by sponsor
- The drug manufacturer can no longer provide the study agent

The reason(s) for protocol therapy discontinuation, the reason(s) for study removal, and the corresponding dates must be documented in the Case Report Form (CRF).

5.5 Duration of Follow Up

Patients will be followed for 30 days after removal from study or until death, whichever occurs first. Patients removed from study for unacceptable adverse event(s) will be followed until resolution or stabilization of the adverse event.

6. DOSING DELAYS/DOSE MODIFICATIONS

Dose Level	AZD1775 Dose (21 Day Cycle)
Starting Dose	300 mg daily days 1-5, 8-12
-1	250 mg daily days 1-5, 8-12
-2	200 mg daily days 1-5, 8-12

Toxicity will be assessed utilizing the NCI CTCAE v5.0 (http://evs.nci.nih.gov/ftp1/CTCAE/CTCAE), unless otherwise specified.

Dose adjustments will be based on the organ system exhibiting the greatest degree of toxicity. Dose reductions or holds and initiation of supportive care are allowed as clinically indicated by the treating physician. A maximum of 2 dose reductions for AZD1775 will be allowed. Patients requiring >2 dose reductions will be discontinued from the study drug.

Any patient requiring a toxicity-related dose delay of more than 21 days from the intended day of the next scheduled dose must be discontinued from the study unless there is approval from the Medical Monitor for the patient to continue.

6.1 Dose modifications due to hematologic toxicity

Complete blood counts (CBC) will be obtained for all patients at the beginning of each treatment cycle (Day 1). If hematologic toxicity occurs (see **Table 1** and **Table 2**), treatment should be held and ANC and platelets should be monitored weekly (or more often as clinically indicated) until recovery.

Table 1 Day 1 Hematologic Dose Modifications

**Treatment Day Blood Counts and Toxicity**			
**ANC**		**Platelets**	**Action**
≥1000/µL	And	≥75,000/µL	No dose modification or interruption

Table 1 Day 1 Hematologic Dose Modifications

**Treatment Day Blood Counts and Toxicity**			
<1000/µL	Or	<75,000/µL	Delay by 1 week intervals until recovery

If hematologic toxicity parameters do not recover within 21 days, the patient should be removed from the study treatment.

Table 2 Neutropenia, Infection, Febrile Neutropenia Dose Modifications and Management

**Any Day**	
**Grade 3 neutropenic fever** (ANC <1000/µL + Temperature ≥101°F [38.5°C]) or neutropenic infection<br><br>**Documented infection with Grade 3 neutropenia** (ANC <1000/µL)<br><br>**Grade 4 neutropenia** (ANC <500/µL >7 days<br><br>**Grade 4 thrombocytopenia** (platelet count <25,000/µL >7 days)	**Hold dose until recovery.**<br>**Then, upon resuming dosing, reduce AZD1775 to the next lower dose level[a].**
**Grade 4 febrile neutropenia or Grade 4 infection with neutropenia** (both defined as septic shock)<br><br>**Thrombocytopenic haemorrhage** (gross occult bleeding) associated with a platelet count <50,000/µL	**Discontinue treatment and follow for disease progression.**

[a] No more than two dose reductions will be allowed for any patient. Patients requiring additional dose modifications due to toxicity will discontinue study treatment.

6.2 Non-hematologic toxicity management guidelines

Substantial acute toxicities should be managed as medically indicated and with temporary suspension of investigational product, as appropriate. Dose reductions or holds and initiation of supportive care are allowed as clinically indicated by the treatment physician.

Dose reductions of AZD1775 should be considered only if toxicity is considered to be related to AZD1775. Dose re-escalation is not permitted. In general, if a patient experiences a G1/G2 non-hematological toxicity, no dose modification is required (except QTc prolongation, see Table 5). If a patient experiences a G3 or G4 toxicity which is not attributable to the disease or disease

related processes under investigation, dosing will be interrupted and/or the dose reduced and supportive therapy administered as required.

Any patient who develops a Grade 3 or 4 non-hematologic toxicity that does not resolve to ≤ Grade 1 within 21 days should be removed from the study treatment unless approved by the Medical Monitor.

6.2.1 Management of QT/QTc Prolongation

Prolonged QTc interval toxicity should be managed according to Table 3 below.

6.2.1.1 QT/QTc Evaluation

Any time QTc is evaluated:

- If QTcF is > 450 ms for males (ie, grade 1 or higher) or > 470 ms for females:
  - Check potassium and magnesium serum levels
  - Correct any identified hypokalemia and/or hypomagnesemia and repeat ECG to confirm QTc;
- When HR is between 60-100 bpm, manual measurement of QT interval is NOT required;
- When HR < 60 or >100 bpm, manual measurement of QT interval by cardiologist is required, with Fridericia correction applied to that manual measurement to determine the QTc;
- Any questions or concerns about ECG readings will be reviewed with a cardiologist.

6.2.1.2 Concomitant Medications Known to Prolong QTc

To the extent possible, concurrent use of AZD1775 with drugs known to cause clinically significant QT prolongation should be avoided. Such drugs may be identified at the Credible Meds website (http://crediblemeds.org/login). The Credible Meds website requires free user registration to view the list of clinically relevant QT prolonging drugs, eg, those known to carry a risk of causing Torsades de Pointes. QT prolonging drugs to be avoided during this trial are shown in the Credible Meds list of "drugs with known TdP risk."

When concurrent use of AZD1775 with any drug on the Credible Meds list of "drugs with known TdP risk" cannot be avoided, review QTc prior to concurrent use. If pre-concurrent use QTc is:

- Grade 0 (< 450 ms for males or < 470 ms for females), follow-up QTc evaluation should be done at the next scheduled visit.
- Grade 1 (450-480 ms for males or 470-480 ms for females), follow-up QTc evaluation should be done within 8 days after concurrent use starts.
- Grade 2 (481-500 ms for males and females), withhold AZD1775 until follow-up ECG at next possible opportunity shows QTc ≤ grade 1 (≤ 480 ms); evaluate QTc within 8 days after reintroduction of AZD1775.

6.2.1.3 Management of New-Onset Dysrhythmia/Near-Syncope/Syncope

Any new onset of dysrhythmia on ECG will be reviewed and managed with input from

cardiology.

For any episode of syncope (grade 3) or near-syncope (pre-syncope grade 2) or QTc grade 3 or 4 (> 500 ms on 2 ECGs), see Table 3.

Table 3. AZD1775 dose modifications for QTcF interval prolongation, pre-syncope, and syncope:

**QTcF Prolongation**	
Grade 2 (481 – 500 ms for males and females)	Continue treatment; no change in dose.
Grade 3 (> 500 ms for males and females)	• Hold study drug.<br>• Check and immediately administer potassium to achieve levels ≥ 4 mEq and magnesium to levels ≥ 2 mEq; consider chronic oral supplementation of potassium and/or magnesium.<br>• Review with Principal Investigator prior to patient's next scheduled treatment, considering the following options:<br>o Hold study drug until QTc ≤ 480 msec<br>o When recovered to ≤ 480 msec, restart AZD1775 cautiously, with additional QTc monitoring within 3-8 days from reintroduction.<br>• If QTc prolongation is thought to be related to study drug, reduce by one dose level when resuming treatment.<br>• For recurrent grade 3 QTc prolongation thought to be related to study drug and despite dose reduction, discontinue protocol therapy.
Grade 4	Discontinue protocol therapy
**Pre-Syncope (Grade 2)**<br>**1.1.1.1 Syncope (Grade 3)**	• Hold study drug<br>• Obtain ECG for cardiology review/consultation. If ECG shows new dysrhythmia or QTc ≥ grade 2:<br>o Consider hospitalization for monitoring with cardiology consultation<br>o Follow instructions above for grade 3 QTc interval<br>• For recurrent syncope or near-syncope thought

	to be related to study drug, discontinue protocol therapy

6.3 Diarrhea

Due to frequent reports of diarrhea with AZD1775 administration, vigorous anti-diarrheal treatment loperamide (Imodium) is required at the **first** onset of diarrhea according to American Society of Clinical Oncology (ASCO) guidelines. Oral loperamide (Imodium) 4 mg should be administered every 2 hours until diarrhea-free for at least 12 hours. The first dose of loperamide could be lowered to 2 mg if the diarrhea is recurrent and if, in the opinion of the treating physician, the diarrhea is not severe.

Patients should be instructed to notify the Investigator or research staff of the occurrence of bloody or black stools, symptoms of dehydration, fever, inability to take liquids by mouth, and inability to control diarrhea within 24 hours of using loperamide or other prescribed antidiarrheal medications.

If diarrhea is severe (i.e., requiring intravenous [IV] rehydration) and/or associated with fever or severe neutropenia (Grade 3 or 4), broad-spectrum antibiotics must be prescribed. Patients with severe diarrhea or any diarrhea associated with severe nausea or vomiting should be hospitalized for IV hydration and correction of electrolyte imbalances.

6.4 Nausea and vomiting (mandatory antiemetic prophylaxis)

All patients must receive a 5-HT3 antagonist, ondansetron (Zofran) 8 mg PO or granisetron (Kytril) 1 mg PO prior to each dose of AZD1775. Additional doses of 5-HT3 antagonist may be used if needed. In addition, dexamethasone 4 mg PO will be given with each AZD1775 dose as a minimum on the first day of dosing AZD1775 of every 3-5 days dosing period, unless contraindicated or not well-tolerated. Dexamethasone may be continued on further days of dosing, potentially at a lower dose. Dexamethasone or the 5-HT3 antagonist may be given by IV.

Promethazine (Phenergan), prochlorperazine (Compazine), olanzapine, and benzodiazepine may still be used as additional adjunctive treatments during AZD1775 therapy.

Please note: aprepitant [Emend] and fosaprepitant are not permitted due to known DDIs.

Patients should be strongly encouraged to maintain liberal oral fluid intake. Suitable alternative medications may be used, with adequate justification, in those studies where the use of any of the above medications might interfere with other study procedures or are deemed insufficient.

7. ADVERSE EVENTS: LIST AND REPORTING REQUIREMENTS

Adverse event (AE) monitoring and reporting is a routine part of every clinical trial. The following list of AEs (Section 7.1) and the characteristics of an observed AE (Sections 7.2 and 7.3) will determine whether the event requires expedited reporting via the CTEP Adverse Event Reporting System (CTEP-AERS) **in addition** to routine reporting.

7.1 Comprehensive Adverse Events and Potential Risks List (CAEPR)

Comprehensive Adverse Events and Potential Risks list (CAEPR)
for
AZD1775 (adavosertib, NSC 751084)

The Comprehensive Adverse Events and Potential Risks list (CAEPR) provides a single list of reported and/or potential adverse events (AE) associated with an agent using a uniform presentation of events by body system. In addition to the comprehensive list, a subset, the Specific Protocol Exceptions to Expedited Reporting (SPEER), appears in a separate column and is identified with bold and italicized text. This subset of AEs (SPEER) is a list of events that are protocol specific exceptions to expedited reporting to NCI (except as noted below). Refer to the 'CTEP, NCI Guidelines: Adverse Event Reporting Requirements' http://ctep.cancer.gov/protocolDevelopment/electronic_applications/docs/aeguidelines.pdf for further clarification. *Frequency is provided based on 323 patients.* Below is the CAEPR for AZD1775 (adavosertib).

> **NOTE**: Report AEs on the SPEER **ONLY IF** they exceed the grade noted in parentheses next to the AE in the SPEER. If this CAEPR is part of a combination protocol using multiple investigational agents and has an AE listed on different SPEERs, use the lower of the grades to determine if expedited reporting is required.

Version 2.7, April 27, 2020[1]

**Adverse Events with Possible Relationship to AZD1775 (adavosertib) (CTCAE 5.0 Term) [n= 323]**			**Specific Protocol Exceptions to Expedited Reporting**
**Likely (>20%)**	**Less Likely (<=20%)**	**Rare but Serious (<3%)**	
BLOOD AND LYMPHATIC SYSTEM DISORDERS			
	Anemia		***Anemia (Gr 3)***
		Febrile neutropenia	
CARDIAC DISORDERS			
		Atrial fibrillation	
		Supraventricular tachycardia	
GASTROINTESTINAL DISORDERS			
	Abdominal pain		***Abdominal pain (Gr 2)***
	Constipation		***Constipation (Gr 2)***
Diarrhea			***Diarrhea (Gr 3)***
	Dyspepsia		
		Gastrointestinal hemorrhage[2]	

**Adverse Events with Possible Relationship to AZD1775 (adavosertib) (CTCAE 5.0 Term) [n= 323]**			**Specific Protocol Exceptions to Expedited Reporting**
**Likely (>20%)**	**Less Likely (<=20%)**	**Rare but Serious (<3%)**	
	Mucositis oral		***Mucositis oral (Gr 2)***
Nausea			***Nausea (Gr 3)***
Vomiting			***Vomiting (Gr 3)***
GENERAL DISORDERS AND ADMINISTRATION SITE CONDITIONS			
	Edema limbs		***Edema limbs (Gr 2)***
Fatigue			***Fatigue (Gr 3)***
	Fever		***Fever (Gr 2)***
HEPATOBILIARY DISORDERS			
		Hepatobiliary disorders - Other (hepatitis)	
INFECTIONS AND INFESTATIONS			
	Infection[3]		***Infection[3] (Gr 3)***
INVESTIGATIONS			
	Alanine aminotransferase increased		***Alanine aminotransferase increased (Gr 3)***
		Electrocardiogram QT corrected interval prolonged	
	Lymphocyte count decreased		
	Neutrophil count decreased		***Neutrophil count decreased (Gr 4)***
	Platelet count decreased		***Platelet count decreased (Gr 4)***
	Weight loss		
	White blood cell decreased		***White blood cell decreased (Gr 4)***
METABOLISM AND NUTRITION DISORDERS			
	Anorexia		***Anorexia (Gr 2)***
	Dehydration		
	Hypokalemia		***Hypokalemia (Gr 2)***
	Hypomagnesemia		***Hypomagnesemia (Gr 2)***
MUSCULOSKELETAL AND CONNECTIVE TISSUE DISORDERS			
	Back pain		***Back pain (Gr 2)***
	Muscle cramp		
	Myalgia		***Myalgia (Gr 2)***
NERVOUS SYSTEM DISORDERS			

Adverse Events with Possible Relationship to AZD1775 (adavosertib) (CTCAE 5.0 Term) [n= 323]			Specific Protocol Exceptions to Expedited Reporting
**Likely (>20%)**	**Less Likely (<=20%)**	**Rare but Serious (<3%)**	
`	Dizziness		***Dizziness (Gr 2)***
	Headache		***Headache (Gr 2)***
		Intracranial hemorrhage	
PSYCHIATRIC DISORDERS			
	Insomnia		
RESPIRATORY, THORACIC AND MEDIASTINAL DISORDERS			
	Cough		***Cough (Gr 2)***
	Dyspnea		***Dyspnea (Gr 2)***
		Hypoxia	
SKIN AND SUBCUTANEOUS TISSUE DISORDERS			
	Rash[4]		***Rash[4] (Gr 2)***
VASCULAR DISORDERS			
		Phlebitis	

[1]This table will be updated as the toxicity profile of the agent is revised. Updates will be distributed to all Principal Investigators at the time of revision. The current version can be obtained by contacting PIO@CTEP.NCI.NIH.GOV. Your name, the name of the investigator, the protocol and the agent should be included in the e-mail.

[2]Gastrointestinal hemorrhage includes Anal hemorrhage, Cecal hemorrhage, Colonic hemorrhage, Duodenal hemorrhage, Esophageal hemorrhage, Esophageal varices hemorrhage, Gastric hemorrhage, Hemorrhoidal hemorrhage, Ileal hemorrhage, Intra-abdominal hemorrhage, Jejunal hemorrhage, Lower gastrointestinal hemorrhage, Oral hemorrhage, Pancreatic hemorrhage, Rectal hemorrhage, Retroperitoneal hemorrhage, and Upper gastrointestinal hemorrhage under the GASTROINTESTINAL DISORDERS SOC.

[3]Infection includes all 75 sites of infection under the INFECTIONS AND INFESTATIONS SOC.

[4]Rash may include rash, erythema, eczema, and rash maculo-papular.

[5]Peripheral neuropathy includes both peripheral motor neuropathy and peripheral sensory neuropathy.

[6]Acute kidney injury includes renal impairment and acute renal insufficiency.

Adverse events reported on AZD1775 (adavosertib) trials, but for which there is insufficient evidence to suggest that there was a reasonable possibility that AZD1775 (adavosertib) caused the adverse event:

BLOOD AND LYMPHATIC SYSTEM DISORDERS - Blood and lymphatic system disorders - Other (pancytopenia); Blood and lymphatic system disorders - Other (thrombocytosis); Blood and lymphatic system disorders - Other (right leg deep vein thrombosis); Leukocytosis
Cardiac disorders - Cardiac disorders - Other (cardiomegaly); Chest pain - cardiac; Myocardial infarction; Palpitations; Sinus bradycardia; Sinus tachycardia
EAR AND LABYRINTH DISORDERS - Ear pain; Hearing impaired; Tinnitus
EYE DISORDERS - Blurred vision; Cataract; Eye disorders - Other (eye swelling); Eye pain; Keratitis; Photophobia; Scleral disorder; Vision decreased; Watering eyes
GASTROINTESTINAL DISORDERS - Abdominal distension; Anal pain; Ascites; Belching; Bloating; Cheilitis; Colitis; Colonic obstruction; Dry mouth; Duodenal ulcer; Dysphagia; Enterocolitis; Flatulence; Gastric ulcer; Gastritis; Hemorrhoids; Oral pain; Rectal pain; Small intestinal obstruction
GENERAL DISORDERS AND ADMINISTRATION SITE CONDITIONS - Chills; Death NOS; Edema trunk; Flu like symptoms; Gait disturbance; General disorders and administration site conditions - Other (catheter site pain); Infusion site extravasation; Malaise; Non-cardiac chest pain; Pain
IMMUNE SYSTEM DISORDERS - Allergic reaction; Anaphylaxis; Cytokine release syndrome
INJURY, POISONING AND PROCEDURAL COMPLICATIONS - Fall; Injury, poisoning and procedural complications - Other (excoriation); Injury, poisoning and procedural complications - Other (ligament sprain)
INVESTIGATIONS - Alkaline phosphatase increased; Aspartate aminotransferase increased; Blood bilirubin increased; Creatinine increased; GGT increased; Investigations - Other (blood urea increased); Lymphocyte count increased
METABOLISM AND NUTRITION DISORDERS - Alkalosis; Hypercalcemia; Hyperglycemia; Hyperkalemia; Hyperuricemia; Hypoalbuminemia; Hypocalcemia; Hyponatremia; Hypophosphatemia; Tumor lysis syndrome
MUSCULOSKELETAL AND CONNECTIVE TISSUE DISORDERS - Arthralgia; Arthritis; Bone pain; Flank pain; Generalized muscle weakness; Muscle weakness lower limb; Musculoskeletal and connective tissue disorder - Other (groin pain); Neck pain; Pain in extremity
NEOPLASMS BENIGN, MALIGNANT AND UNSPECIFIED (INCL CYSTS AND POLYPS) - Neoplasms benign, malignant and unspecified (incl cysts and polyps) - Other (carcinoid tumor); Tumor pain
NERVOUS SYSTEM DISORDERS - Central nervous system necrosis; Cognitive disturbance; Depressed level of consciousness; Dysesthesia; Dysgeusia; Encephalopathy; Lethargy; Nervous system disorders - Other (hemiparesis); Paresthesia; Peripheral neuropathy[5]; Presyncope; Somnolence; Syncope
PSYCHIATRIC DISORDERS - Agitation; Anxiety; Confusion; Depression
RENAL AND URINARY DISORDERS - Acute kidney injury[6]; Hematuria; Urinary frequency; Urinary incontinence; Urinary retention; Urinary tract pain
REPRODUCTIVE SYSTEM AND BREAST DISORDERS - Genital edema; Reproductive system and breast disorders - Other (female genital tract fistula)

RESPIRATORY, THORACIC AND MEDIASTINAL DISORDERS - Allergic rhinitis; Apnea; Bronchopulmonary hemorrhage; Epistaxis; Hiccups; Nasal congestion; Pleural effusion; Pneumonitis; Pulmonary hypertension; Respiratory, thoracic and mediastinal disorders - Other (diaphragmalgia); Voice alteration; Wheezing
SKIN AND SUBCUTANEOUS TISSUE DISORDERS - Alopecia; Bullous dermatitis; Dry skin; Hyperhidrosis; Pain of skin; Palmar-plantar erythrodysesthesia syndrome; Pruritus; Purpura; Rash acneiform; Skin ulceration; Urticaria
VASCULAR DISORDERS - Flushing; Hematoma; Hot flashes; Hypertension; Hypotension; Thromboembolic event

Note: AZD1775 (adavosertib) in combination with other agents could cause an exacerbation of any adverse event currently known to be caused by the other agent, or the combination may result in events never previously associated with either agent.

7.2 Adverse Event Characteristics

- **CTCAE term (AE description) and grade:** The descriptions and grading scales found in the revised NCI Common Terminology Criteria for Adverse Events (CTCAE) version 5.0 will be utilized for AE reporting. All appropriate treatment areas should have access to a copy of the CTCAE version 5.0. A copy of the CTCAE version 5.0 can be downloaded from the CTEP web site http://ctep.cancer.gov/protocolDevelopment/electronic_applications/ctc.htm.

- **For expedited reporting purposes only:**
  - AEs for the agent that are ***bold and italicized*** in the CAEPR (*i.e.*, those listed in the SPEER column, Section 7.1) should be reported through CTEP-AERS only if the grade is above the grade provided in the SPEER.

- **Attribution** of the AE:
  - Definite – The AE *is clearly related* to the study treatment.
  - Probable – The AE *is likely related* to the study treatment.
  - Possible – The AE *may be related* to the study treatment.
  - Unlikely – The AE *is doubtfully related* to the study treatment.
  - Unrelated – The AE *is clearly NOT related* to the study treatment.

7.3 Expedited Adverse Event Reporting

7.3.1 Expedited AE reporting for this study must use CTEP-AERS (CTEP Adverse Event Reporting System), accessed via the CTEP Web site (https://eapps-ctep.nci.nih.gov/ctepaers). The reporting procedures to be followed are presented in the "NCI Guidelines for Investigators: Adverse Event Reporting Requirements for DCTD (CTEP and CIP) and DCP INDs and IDEs" which can be downloaded from the CTEP Web site (http://ctep.cancer.gov/protocolDevelopment/electronic_applications/adverse_events.htm). These requirements are briefly outlined in the tables below (Section 7.3.3).

In the rare occurrence when Internet connectivity is lost, a 24-hour notification is to be made to CTEP by telephone at 301-897-7497. Once Internet connectivity is restored, the 24-hour notification phoned in must be entered electronically into CTEP-AERS by the original submitter at the site.

7.3.2 Distribution of Adverse Event Reports

CTEP-AERS is programmed for automatic electronic distribution of reports to the following individuals: Principal Investigator and Adverse Event Coordinator(s) (if applicable) of the Corresponding Organization or Lead Organization, the local treating

physician, and the Reporter and Submitter. CTEP-AERS provides a copy feature for other e-mail recipients.

7.3.3 Expedited Reporting Guidelines

Use the NCI protocol number and the protocol-specific patient ID assigned during trial registration on all reports.

Note: A death on study requires both routine and expedited reporting, regardless of causality. Attribution to treatment or other cause must be provided.

Death due to progressive disease should be reported as **Grade 5 "Neoplasms benign, malignant and unspecified (incl cysts and polyps) - Other (Progressive Disease)"** under the system organ class (SOC) of the same name. Evidence that the death was a manifestation of underlying disease (*e.g.*, radiological changes suggesting tumor growth or progression: clinical deterioration associated with a disease process) should be submitted.

Phase 1 and Early Phase 2 Studies: Expedited Reporting Requirements for Adverse Events that Occur on Studies under an IND/IDE within 30 Days of the Last Administration of the Investigational Agent/Intervention [1, 2]

FDA REPORTING REQUIREMENTS FOR SERIOUS ADVERSE EVENTS (21 CFR Part 312)
NOTE: Investigators **MUST** immediately report to the sponsor (NCI) **ANY** Serious Adverse Events, whether or not they are considered related to the investigational agent(s)/intervention (21 CFR 312.64)
An adverse event is considered serious if it results in **ANY** of the following outcomes:

1) Death
2) A life-threatening adverse event
3) An adverse event that results in inpatient hospitalization or prolongation of existing hospitalization for ≥ 24 hours
4) A persistent or significant incapacity or substantial disruption of the ability to conduct normal life functions
5) A congenital anomaly/birth defect.
6) Important Medical Events (IME) that may not result in death, be life threatening, or require hospitalization may be considered serious when, based upon medical judgment, they may jeopardize the patient or subject and may require medical or surgical intervention to prevent one of the outcomes listed in this definition. (FDA, 21 CFR 312.32; ICH E2A and ICH E6).

ALL SERIOUS adverse events that meet the above criteria MUST be immediately reported to the NCI via electronic submission within the timeframes detailed in the table below.

Hospitalization	Grade 1 and Grade 2 Timeframes	Grade 3-5 Timeframes
Resulting in Hospitalization ≥ 24 hrs	10 Calendar Days	24-Hour 5 Calendar Days
Not resulting in Hospitalization ≥ 24 hrs	Not required	

NOTE: Protocol specific exceptions to expedited reporting of serious adverse events are found in the Specific Protocol Exceptions to Expedited Reporting (SPEER) portion of the CAEPR.

Expedited AE reporting timelines are defined as:

- "24-Hour; 5 Calendar Days" - The AE must initially be submitted electronically within 24 hours of learning of the AE, followed by a complete expedited report within 5 calendar days of the initial 24-hour report.
- "10 Calendar Days" - A complete expedited report on the AE must be submitted electronically within 10 calendar days of learning of the AE.

[1]Serious adverse events that occur more than 30 days after the last administration of investigational agent/intervention and have an attribution of possible, probable, or definite require reporting as follows:
Expedited 24-hour notification followed by complete report within 5 calendar days for:

- All Grade 3, 4, and Grade 5 AEs

Expedited 10 calendar day reports for:

- Grade 2 AEs resulting in hospitalization or prolongation of hospitalization

[2]For studies using PET or SPECT IND agents, the AE reporting period is limited to 10 radioactive half-lives, rounded UP to the nearest whole day, after the agent/intervention was last administered. Footnote "1" above applies after this reporting period.

Effective Date: May 5, 2011

7.3.4. Events of Special Interest

Specific adverse events, or groups of adverse events, will be followed as part of standard safety monitoring activities by the Sponsor. These events will be reported to the Sponsor via CTEPAERS within 24 hours of awareness following the procedure described above for SAEs and will require enhanced data collection. All Events of Special Interest will be submitted within 24 hours of awareness even if they do not meet serious criteria.

7.3.4.1 Cardiac Events

- Grade 2 or greater QT prolongation
- Any treatment-emergent cardiac AEs of Grade 2 or higher.

7.4 Routine Adverse Event Reporting

All Adverse Events **must** be reported in routine study data submissions. **AEs reported expeditiously through CTEP-AERS must also be reported in routine study data submissions.**

Adverse event data collection and reporting, which are required as part of every clinical trial, are done to ensure the safety of patients enrolled in the studies as well as those who will enroll in future studies using similar agents. AEs are reported in a routine manner at scheduled times during the trial using Medidata Rave. For this trial the Adverse Event CRF is used for routine AE reporting in Rave.

7.5 Pregnancy

Although not an adverse event in and of itself, pregnancy as well as its outcome must be

documented via **CTEP-AERS**. In addition, the ***Pregnancy Information Form*** included within the NCI Guidelines for Adverse Event Reporting Requirements must be completed and submitted to CTEP. Any pregnancy occurring in a patient or patient's partner from the time of consent to 90 days after the last dose of study drug must be reported and then followed for outcome. Newborn infants should be followed until 30 days old. Please see the "NCI Guidelines for Investigators: Adverse Event Reporting Requirements for DCTD (CTEP and CIP) and DCP INDs and IDEs" (at http://ctep.cancer.gov/protocolDevelopment/adverse_effects.htm) for more details on how to report pregnancy and its outcome to CTEP.

7.6 Secondary Malignancy

A *secondary malignancy* is a cancer caused by treatment for a previous malignancy (*e.g.*, treatment with investigational agent/intervention, radiation or chemotherapy). A secondary malignancy is not considered a metastasis of the initial neoplasm.

CTEP requires all secondary malignancies that occur following treatment with an agent under an NCI IND/IDE be reported expeditiously via CTEP-AERS. Three options are available to describe the event:

- Leukemia secondary to oncology chemotherapy (*e.g.*, acute myelocytic leukemia [AML])
- Myelodysplastic syndrome (MDS)
- Treatment-related secondary malignancy

Any malignancy possibly related to cancer treatment (including AML/MDS) should also be reported via the routine reporting mechanisms outlined in each protocol.

A second malignancy is one unrelated to the treatment of a prior malignancy (and is **NOT** a metastasis from the initial malignancy). Second malignancies require **ONLY** routine AE reporting unless otherwise specified.

8. PHARMACEUTICAL INFORMATION

A list of the adverse events and potential risks associated with the investigational administered in this study can be found in Section 7.1.

8.1 AZD1775

Availability

AZD1775 is an investigational agent supplied to investigators by the Division of Cancer Treatment and Diagnosis (DCTD), NCI.

Chemical Name: 2-allyl-1-[6-(1-hydroxy-1-methyl-ethyl)-2-pyridyl]-6-[4-(4-methylpiperazin-1-yl)anilino]pyrazolo[3,4-d]pyrimidin-3-one hemihydrate

Other Names: MK-1775, Adavosertib

Classification: inhibitor of WEE1 kinase

CAS: 1277170-60-1

Molecular Formula: $C_{27}H_{32}N_8O_2 \cdot 0.5H_2O$ **M.W.:** 500.6

Mode of Action: AZD1775 inhibits WEE1 which phosphorylates and inhibits cyclin-dependent kinases 1 (CDK1) and 2 (CDK2), and is involved in regulation of the intra-S and G2 cell cycle checkpoints. In in vitro and in vivo preclinical models, AZD1775 selectively enhanced chemotherapy induced death of cells deficient in p53 signaling.

Description: AZD1775 is a crystalline, hemihydrate form of the drug substance.

How Supplied: AZD1775 (MK-1775) is supplied by AstraZeneca and distributed by the Pharmaceutical Management Branch, CTEP/DCTD/NCI as capsules available in 25 mg (yellow color, size 2 gelatin capsule) and 100 mg (orange color, size 2 gelatin capsule) strengths. The dry-filled capsules consist of a roller-compacted granule of drug substance, lactose monohydrate, microcrystalline cellulose, croscarmellose sodium, and magnesium stearate. Each high density polyethylene (HDPE) bottle contains 20 capsules.

Storage: Store at 2 to 30˚C (36 to 86˚F). Do not freeze.
If a storage temperature excursion is identified, promptly return AZD1775 to between 2-30°C and quarantine the supplies. Provide a detailed report of the excursion (including documentation of temperature monitoring and duration of the excursion) to PMBAfterHours@mail.nih.gov for determination of suitability.

Stability: Shelf-life stability studies of AZD1775 capsules are ongoing.

Route of Administration: Oral administration. Take AZD1775 two hours before a meal or two hours after a meal. Capsules should not be opened.

Potential Drug Interactions: AZD1775 is primarily metabolized by CYP3A4 and is a weak, time-dependent inhibitor of CYP3A4. Avoid concomitant CYP3A4 moderate or strong inhibitors/inducers, and sensitive substrates with a narrow therapeutic index. AZD1775 is also a weak inhibitor of CYP2C19. Caution should be exercised with concomitant administration of sensitive substrates or substrates with a narrow therapeutic index.

In vitro transporter studies have shown that AZD1775 was an inhibitor of OATP1B1, OATP1B3, MATE1, MATE2K, P-glycoprotein (P-gp) and breast cancer resistance protein (BCRP), and a substrate for P-gp and BCRP. The PK parameters of AZD1775 could be altered if AZD1775 is coadministered with P-gp and BCRP inhibitors/inducers, and there is potential for drug-drug interactions when coadministered with OATP1B1, OATP1B3, MATE1, MATE2K, P-gp and BCRP substrates. This finding is particularly relevant for drugs administered orally where exposure is normally limited by BCRP-mediated efflux, in particular some statins. Modelling has

predicted a substantial increase in the exposure of atorvastatin when coadministered with AZD1775 and the use of atorvastatin is therefore prohibited.

8.1.1 Agent Ordering and Agent Accountability

8.1.1.1 NCI-supplied agents may be requested by eligible participating Investigators (or their authorized designee) at each participating institution. The CTEP-assigned protocol number must be used for ordering all CTEP-supplied investigational agents. The eligible participating investigators at each participating institution must be registered with CTEP, DCTD through an annual submission of FDA Form 1572 (Statement of Investigator), NCI Biosketch, Agent Shipment Form, and Financial Disclosure Form (FDF). If there are several participating investigators at one institution, CTEP-supplied investigational agents for the study should be ordered under the name of one lead participating investigator at that institution.

Active CTEP-registered investigators and investigator-designated shipping designees and ordering designees can submit agent requests through the PMB Online Agent Order Processing (OAOP) application. Access to OAOP requires the establishment of a CTEP Identity and Access Management (IAM) account and the maintenance of an "active" account status and a "current" password. For questions about drug orders, transfers, returns, or accountability, call or email PMB any time. Refer to the PMB's website for specific policies and guidelines related to agent management.

In general, sites may order initial agent supplies when a subject is being screened for enrollment onto the study.

8.1.1.2 Agent Inventory Records – The investigator, or a responsible party designated by the investigator, must maintain a careful record of the receipt, dispensing and final disposition of all agents received from the PMB using the appropriate NCI Investigational Agent (Drug) Accountability Record (DARF) available on the CTEP forms page. Store and maintain separate NCI Investigational Agent Accountability Records for each agent, strength, formulation and ordering investigator on this protocol.

8.1.2 Investigator Brochure Availability

The current versions of the IBs for the agents will be accessible to site investigators and research staff through the PMB OAOP application. Access to OAOP requires the establishment of a CTEP Identity and Access Management (IAM) account and the maintenance of an "active" account status, a "current" password, and active person registration status. Questions about IB access may be directed to the PMB IB Coordinator via email.

8.1.3 Useful Links and Contacts

- CTEP Forms, Templates, Documents: http://ctep.cancer.gov/forms/
- NCI CTEP Investigator Registration: RCRHelpDesk@nih.gov
- PMB policies and guidelines:

http://ctep.cancer.gov/branches/pmb/agent_management.htm
- PMB Online Agent Order Processing (OAOP) application: https://ctepcore.nci.nih.gov/OAOP/
- CTEP Identity and Access Management (IAM) account: https://ctepcore.nci.nih.gov/iam/index.jsp
- CTEP Associate Registration and IAM account help: ctepreghelp@ctep.nci.nih.gov
- IB Coordinator: IBCoordinator@mail.nih.gov
- PMB email: PMBAfterHours@mail.nih.gov
- PMB phone and hours of service: (240) 276-6575 Monday through Friday between 8:30 am and 4:30 pm (ET)

9. BIOMARKER, CORRELATIVE, AND SPECIAL STUDIES

9.1 Integral biomarkers

Assessment of *SETD2* Mutation Status Using CLIA-Certified Next Generation Sequencing (NGS) Panel

Tumors with pathogenic loss of *SETD2* have been shown to have synthetic lethality with WEE1 inhibitor AZD1775 in pre-clinical studies (Pfister et al. 2015). Therefore, the presence of pathogenic loss of *SETD2* using a CLIA-certified NGS panel is an inclusion criterion for all patients enrolled on study.

The specific CLIA-certified NGS assay to be utilized for Screening is not pre-specified, and may be any CLIA-certified panel that includes the *SETD2* gene as part of the panel (e.g. UCSF500, FoundationOne).

All NGS reports must be sent to the UCSF Molecular Tumor Board for virtual review and confirmation of pathogenic loss of *SETD2*.

UCSF Molecular Tumor Board contact information:

Gregor Krings MD
Molecular Pathologist, UCSF Molecular Tumor Board
San Francisco, CA 94143
Gregor.Krings@ucsf.edu

Each NGS report will be redacted for PHI prior to submission from investigational site to UCSF MTB. In cases where pathogenic loss of *SETD2* cannot be verified, repeat tumor sequencing will be required to assess eligibility status. The expected turn-around time for UCSF Molecular Tumor Board assessment is approximately 2 weeks.

9.2 Integrated Correlative Studies

9.2.1 Association between H3K36me3 Mark with Clinical Outcomes

We propose to evaluate the loss of H3K36me3 mark as an integrated biomarker to evaluate its predictive utility of response to WEE1 inhibition. Based on our pre-clinical data presented above, we hypothesize that loss of this mark will be positively associated with likelihood of tumor response and clinical benefit. If the current study supports this hypothesis, this would form the strong rationale to support CLIA validation of the immunohistochemial assay and prospective evaluation as an integral biomarker used for treatment selection in subsequent clinical studies, and ultimately, may help to select patients for this therapy if FDA approved on the basis of subsequent clinical studies.

The rationale for evaluating loss of H3K36me3 mark, as opposed to solely relying on detection of somatic loss-of-function SETD2 mutations, as a predictive biomarker for WEE1 inhibition, stems from the fact that there are multiple converging mutations, in addition to SETD2, which may ultimately lead to loss of the H3K36me3 mark and subsequent synthetic lethality with WEE1 inhibition. This includes overexpression of KDM4A and KDM4B, as well as H3.3 mutations (G34R/V, K36M), which occur mutually exclusively to SETD2 mutations and are highly prevalent in certain tumor types. For example, KDM4A overexpression occurs in 12% of bladder and ovarian cancers, and H3.3K36M mutations occur in 90% of chondroblastomas.

Moreover, the authors have shown that, cancer cells with KDM4A overexpression, as well as H3.3K36M mutation are hypersensitive to WEE1 inhibition by AZD1775. Therefore, directly assessing for loss of the H3K36me3 mark, rather than selecting for specific mutations in a subset of genes, may ultimately capture a broader array of tumors that would have a synthetic lethal effect with WEE1 inhibition.

Formalin-fixed paraffin embedded tissue will be obtained from an archival tumor specimen during the Screening process for patients enrolled on study, when available. FFPE tissue will be evaluated by immunohistochemical analysis of the H3K36me3 mark in the UCSF Cancer Center Tissue Core. The laboratory will utilize a previously validated antibody with a high degree of specificity for H3K36me3 expression (https://www.diagenode.com/files/products/antibodies/Datasheet_H3K36me3_MAb-183-050.pdf). The expression pattern will be graded by pathologists blinded to clinical treatment outcomes as 0, 1+, 2+, or 3+ based on the rules provided below. Representative images of each category of H3K36me3 staining in lung and kidney cancer samples are also provided below:

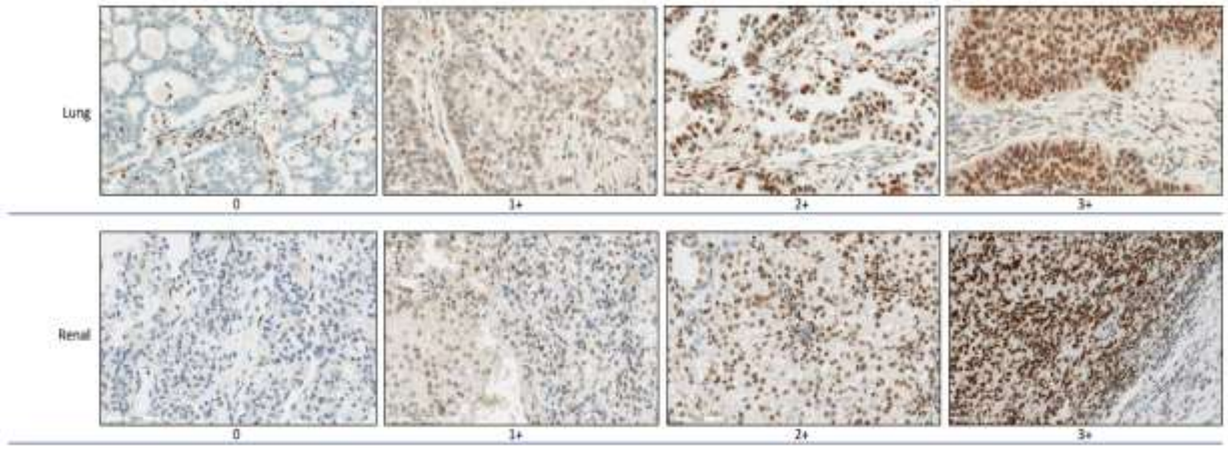

Scoring rules as follows:
0 = no tumor cell nuclear staining
1+ = tumor nuclear staining < stromal staining
2+ = tumor nuclear staining ~ stromal staining
3+ = tumor nuclear staining > stromal staining

Sample score is assigned based on the highest staining score (0-3) observed in 10% or more of tumor cells in section. Scoring will be independently performed by two pathologists blinded to the clinical outcomes on treatment. In cases of discrepancy, the mean of the two individual scores is assigned. Samples without stromal cells present, or those with < 25% tumor content in the section, will be considered inevaluable. Likewise, samples with very high levels of non-specific background staining will be considered inevaluable.

With a total sample size of 30 patients it is estimated that 20 patients (67%) will have evaluable archival tumor for this correlative assay. A sample size of 20 archival tumor specimens will provide sufficient number to demonstrate feasibility of H3K36me3 immunohistochemical staining from FFPE tissue, and allow preliminary assessment of the association between IHC staining with clinical outcomes (see Section 13.4 for Statistical Methods)

9.2.1.1 Collection of Specimen(s)

Archival FFPE tumor block and/or unstained FFPE slides (minimum 5 slides) will be collected during Screening for all patients with available archival tumor tissue.

9.2.1.2 Handling of Specimens(s)

Samples should be kept at room temperature. Subject study ID number should be affixed to specimen label prior to shipment.

9.2.1.3 Shipping of Specimen(s)

FFPE tumor specimens should be shipped to the following location:

UCSF Helen Diller Family Comprehensive Cancer Center Tissue Core
Attention: Scott Vandenberg
2340 Sutter Street, S-231
Box 0875
San Francisco, CA 94115

9.2.1.4 Site(s) Performing Correlative Study

UCSF Helen Diller Family Comprehensive Cancer Center Tissue Core

10. STUDY CALENDAR

Baseline evaluations are to be conducted within 1 week prior to start of protocol therapy. Scans and x-rays must be done ≤4 weeks prior to the start of therapy. In the event that the patient's condition is deteriorating, laboratory evaluations should be repeated within 48 hours prior to initiation of the next cycle of therapy.

	Pre-Study	C1 D1	C1 D8 (+/- 2 days)	C1 D15 (+/- 2 days)	C2 D1 (+/- 3 days)	C2 D8 (+/- 3 days)	C≥3 D1 (+/- 3 days)	Off Study[d]
Dispense AZD1775 / Drug Diary Assessment		A			A		A	
Informed consent	X							
Demographics	X							
Medical history	X							
Evaluation of NGS Sequencing Report (UCSF Molecular Tumor Board)	X							
Concurrent meds	X	X---X						
Physical exam	X	X	X	X	X		X	X
Vital signs	X	X	X	X	X	X	X	X
Height	X							
Weight	X	X	X	X	X	X	X	X
Performance status	X	X	X	X	X	X	X	X
CBC w/diff, plts	X	X	X	X	X	X	X	X
Serum chemistry[a]	X	X	X	X	X	X	X	X
EKG	X[b]	X[b]			X[b]		X[b]	X[b]
Adverse event evaluation		X--X						X
Tumor measurements	X	Tumor measurements are repeated every 9 weeks (+/- 7 days). Documentation (radiologic) must be provided for patients removed from study for progressive disease.						X
Radiologic evaluation	X	Radiologic measurements should be performed every 9 weeks (+/- 7 days).						X
B-HCG	X[c]	X[c]			X[c]		X[c]	
Archival Tumor Collection and Shipping for H3K36me3 IHC testing	X							

A: AZD1775: Dose as assigned; 300 mg daily days 1-5, 8-12 of every 21 day cycle
a: Albumin, alkaline phosphatase, total bilirubin, bicarbonate, BUN, calcium, chloride, creatinine, LDH, potassium, total protein, SGOT [AST], SGPT [ALT], sodium.
b: ECG must be performed in triplicate for each time point.
c: Serum or urine pregnancy test (women of childbearing potential). C1D1 test may be omitted if HCG test performed during Screening is within 72 hours of C1D1.
d: Off-study evaluation.

11. MEASUREMENT OF EFFECT

11.1 Antitumor Effect – Solid Tumors

For the purposes of this study, patients should be re-evaluated for response every 9 weeks (+/- 7 days). In addition to a baseline scan, confirmatory scans should also be obtained at least 4 weeks following initial documentation of objective response.

Response and progression will be evaluated in this study using the new international criteria proposed by the revised Response Evaluation Criteria in Solid Tumors (RECIST) guideline (version 1.1) (Eisenhauer et al. 2009). Changes in the largest diameter (unidimensional measurement) of the tumor lesions and the shortest diameter in the case of malignant lymph nodes are used in the RECIST criteria.

11.1.1 Definitions

Evaluable for toxicity. All patients will be evaluable for toxicity from the time of their first treatment with AZD1775.

Evaluable for objective response. Only those patients who have received at least one cycle of therapy, and have had their disease re-evaluated will be considered evaluable for response. These patients will have their response classified according to the definitions stated below. (Note: Patients who exhibit objective disease progression prior to the end of cycle 1 will also be considered evaluable.)

Evaluable Non-Target Disease Response. Patients who have lesions present at baseline that are evaluable but do not meet the definitions of measurable disease, have received at least one cycle of therapy, and have had their disease re-evaluated will be considered evaluable for non-target disease. The response assessment is based on the presence, absence, or unequivocal progression of the lesions.

11.1.2 Disease Parameters

Measurable disease. Measurable lesions are defined as those that can be accurately measured in at least one dimension (longest diameter to be recorded) as ≥20 mm (≥2 cm) by chest x-ray or as ≥10 mm (≥1 cm) with CT scan, MRI, or calipers by clinical exam. All tumor measurements must be recorded in millimeters (or decimal fractions of centimeters).

Note: Tumor lesions that are situated in a previously irradiated area are only considered measurable if there has been documented progression of the lesion following radiation treatment.

Malignant lymph nodes. To be considered pathologically enlarged and measurable, a lymph node must be ≥15 mm (≥1.5 cm) in short axis when assessed by CT scan (CT scan slice thickness recommended to be no greater than 5 mm [0.5 cm]). At baseline and in

follow-up, only the short axis will be measured and followed.

Non-measurable disease. All other lesions (or sites of disease), including small lesions (longest diameter <10 mm [<1 cm] or pathological lymph nodes with ≥10 to <15 mm [≥1 to <1.5 cm] short axis), are considered non-measurable disease. Bone lesions, leptomeningeal disease, ascites, pleural/pericardial effusions, lymphangitis cutis/pulmonitis, inflammatory breast disease, and abdominal masses (not followed by CT or MRI), are considered as non-measurable.

Note: Cystic lesions that meet the criteria for radiographically defined simple cysts should not be considered as malignant lesions (neither measurable nor non-measurable) since they are, by definition, simple cysts.

'Cystic lesions' thought to represent cystic metastases can be considered as measurable lesions, if they meet the definition of measurability described above. However, if non-cystic lesions are present in the same patient, these are preferred for selection as target lesions.

Target lesions. All measurable lesions up to a maximum of 2 lesions per organ and 5 lesions in total, representative of all involved organs, should be identified as **target lesions** and recorded and measured at baseline. Target lesions should be selected on the basis of their size (lesions with the longest diameter), be representative of all involved organs, but in addition should be those that lend themselves to reproducible repeated measurements. It may be the case that, on occasion, the largest lesion does not lend itself to reproducible measurement in which circumstance the next largest lesion which can be measured reproducibly should be selected. A sum of the diameters (longest for non-nodal lesions, short axis for nodal lesions) for all target lesions will be calculated and reported as the baseline sum diameters. If lymph nodes are to be included in the sum, then only the short axis is added into the sum. The baseline sum diameters will be used as reference to further characterize any objective tumor regression in the measurable dimension of the disease.

Non-target lesions. All other lesions (or sites of disease) including any measurable lesions over and above the 5 target lesions should be identified as **non-target lesions** and should also be recorded at baseline. Measurements of these lesions are not required, but the presence, absence, or in rare cases unequivocal progression of each should be noted throughout follow-up.

11.1.3 Methods for Evaluation of Measurable Disease

All measurements should be taken and recorded in metric notation using a ruler or calipers. All baseline evaluations should be performed as closely as possible to the beginning of treatment and never more than 4 weeks before the beginning of the treatment.

The same method of assessment and the same technique should be used to characterize

each identified and reported lesion at baseline and during follow-up. Imaging-based evaluation is preferred to evaluation by clinical examination unless the lesion(s) being followed cannot be imaged but are assessable by clinical exam.

Clinical lesions Clinical lesions will only be considered measurable when they are superficial (*e.g.*, skin nodules and palpable lymph nodes) and ≥10 mm (≥1 cm) diameter as assessed using calipers (*e.g.*, skin nodules). In the case of skin lesions, documentation by color photography, including a ruler to estimate the size of the lesion, is recommended.

Chest x-ray Lesions on chest x-ray are acceptable as measurable lesions when they are clearly defined and surrounded by aerated lung. However, CT is preferable.

Conventional CT and MRI This guideline has defined measurability of lesions on CT scan based on the assumption that CT slice thickness is 5 mm (0.5 cm) or less. If CT scans have slice thickness greater than 5 mm (0.5 cm), the minimum size for a measurable lesion should be twice the slice thickness. MRI is also acceptable in certain situations (*e.g.* for body scans).

Use of MRI remains a complex issue. MRI has excellent contrast, spatial, and temporal resolution; however, there are many image acquisition variables involved in MRI, which greatly impact image quality, lesion conspicuity, and measurement. Furthermore, the availability of MRI is variable globally. As with CT, if an MRI is performed, the technical specifications of the scanning sequences used should be optimized for the evaluation of the type and site of disease. Furthermore, as with CT, the modality used at follow-up should be the same as was used at baseline and the lesions should be measured/assessed on the same pulse sequence. It is beyond the scope of the RECIST guidelines to prescribe specific MRI pulse sequence parameters for all scanners, body parts, and diseases. Ideally, the same type of scanner should be used and the image acquisition protocol should be followed as closely as possible to prior scans. Body scans should be performed with breath-hold scanning techniques, if possible.

PET-CT At present, the low dose or attenuation correction CT portion of a combined PET-CT is not always of optimal diagnostic CT quality for use with RECIST measurements. However, if the site can document that the CT performed as part of a PET-CT is of identical diagnostic quality to a diagnostic CT (with IV and oral contrast), then the CT portion of the PET-CT can be used for RECIST measurements and can be used interchangeably with conventional CT in accurately measuring cancer lesions over time. Note, however, that the PET portion of the CT introduces additional data which may bias an investigator if it is not routinely or serially performed.

Ultrasound Ultrasound is not useful in assessment of lesion size and should not be used as a method of measurement. Ultrasound examinations cannot be reproduced in their entirety for independent review at a later date and, because they are operator dependent, it cannot be guaranteed that the same technique and measurements will be taken from one assessment to the next. If new lesions are identified by ultrasound in the course of the study, confirmation by CT or MRI is advised. If there is concern about radiation exposure

at CT, MRI may be used instead of CT in selected instances.

<u>Endoscopy, Laparoscopy</u> The utilization of these techniques for objective tumor evaluation is not advised. However, such techniques may be useful to confirm complete pathological response when biopsies are obtained or to determine relapse in trials where recurrence following complete response (CR) or surgical resection is an endpoint.

<u>Tumor markers</u> Tumor markers alone cannot be used to assess response. If markers are initially above the upper normal limit, they must normalize for a patient to be considered in complete clinical response. Specific guidelines for both CA-125 response (in recurrent ovarian cancer) and PSA response (in recurrent prostate cancer) have been published [*JNCI* 96:487-488, 2004; *J Clin Oncol* 17, 3461-3467, 1999; *J Clin Oncol* 26:1148-1159, 2008]. In addition, the Gynecologic Cancer Intergroup has developed CA-125 progression criteria which are to be integrated with objective tumor assessment for use in first-line trials in ovarian cancer [*JNCI* 92:1534-1535, 2000].

<u>Cytology, Histology</u> These techniques can be used to differentiate between partial responses (PR) and complete responses (CR) in rare cases (*e.g.*, residual lesions in tumor types, such as germ cell tumors, where known residual benign tumors can remain).

The cytological confirmation of the neoplastic origin of any effusion that appears or worsens during treatment when the measurable tumor has met criteria for response or stable disease is mandatory to differentiate between response or stable disease (an effusion may be a side effect of the treatment) and progressive disease.

<u>FDG-PET</u> While FDG-PET response assessments need additional study, it is sometimes reasonable to incorporate the use of FDG-PET scanning to complement CT scanning in assessment of progression (particularly possible 'new' disease). New lesions on the basis of FDG-PET imaging can be identified according to the following algorithm:

a. Negative FDG-PET at baseline, with a positive FDG-PET at follow-up is a sign of PD based on a new lesion.
b. No FDG-PET at baseline and a positive FDG-PET at follow-up: If the positive FDG-PET at follow-up corresponds to a new site of disease confirmed by CT, this is PD. If the positive FDG-PET at follow-up is not confirmed as a new site of disease on CT, additional follow-up CT scans are needed to determine if there is truly progression occurring at that site (if so, the date of PD will be the date of the initial abnormal FDG-PET scan). If the positive FDG-PET at follow-up corresponds to a pre-existing site of disease on CT that is not progressing on the basis of the anatomic images, this is not PD.
c. FDG-PET may be used to upgrade a response to a CR in a manner similar to a biopsy in cases where a residual radiographic abnormality is thought to represent fibrosis or scarring. The use of FDG-PET in this circumstance should be prospectively described in the protocol and supported by disease-specific medical literature for the indication. However, it must be acknowledged that both approaches may lead to false positive CR due to limitations of FDG-PET and biopsy resolution/sensitivity.

Note: A 'positive' FDG-PET scan lesion means one which is FDG avid with an uptake greater than twice that of the surrounding tissue on the attenuation corrected image.

11.1.4 Response Criteria

11.1.4.1 Evaluation of Target Lesions

Complete Response (CR): Disappearance of all target lesions. Any pathological lymph nodes (whether target or non-target) must have reduction in short axis to <10 mm (<1 cm).

Partial Response (PR): At least a 30% decrease in the sum of the diameters of target lesions, taking as reference the baseline sum diameters.

Progressive Disease (PD): At least a 20% increase in the sum of the diameters of target lesions, taking as reference the smallest sum on study (this includes the baseline sum if that is the smallest on study). In addition to the relative increase of 20%, the sum must also demonstrate an absolute increase of at least 5 mm (0.5 cm). (Note: the appearance of one or more new lesions is also considered progressions).

Stable Disease (SD): Neither sufficient shrinkage to qualify for PR nor sufficient increase to qualify for PD, taking as reference the smallest sum diameters while on study.

11.1.4.2 Evaluation of Non-Target Lesions

Complete Response (CR): Disappearance of all non-target lesions and normalization of tumor marker level. All lymph nodes must be non-pathological in size (<10 mm [<1 cm] short axis).

Note: If tumor markers are initially above the upper normal limit, they must normalize for a patient to be considered in complete clinical response.

Non-CR/Non-PD: Persistence of one or more non-target lesion(s) and/or maintenance of tumor marker level above the normal limits.

Progressive Disease (PD): Appearance of one or more new lesions and/or *unequivocal progression* of existing non-target lesions. *Unequivocal progression* should not normally trump target lesion status. It must be representative of overall disease status change, not a single lesion increase.

Although a clear progression of "non-target" lesions only is exceptional, the opinion of the treating physician should prevail in such circumstances, and the progression status should be confirmed at a later time by the review panel (or Principal Investigator).

11.1.4.3 Evaluation of Best Overall Response

The best overall response is the best response recorded from the start of the treatment until disease progression/recurrence (taking as reference for progressive disease the smallest measurements recorded since the treatment started). The patient's best response assignment will depend on the achievement of both measurement and confirmation criteria.

For Patients with Measurable Disease (*i.e.*, Target Disease)

Target Lesions	Non-Target Lesions	New Lesions	Overall Response	Best Overall Response when Confirmation is Required*
CR	CR	No	CR	≥4 wks. Confirmation**
CR	Non-CR/Non-PD	No	PR	≥4 wks. Confirmation**
CR	Not evaluated	No	PR	
PR	Non-CR/Non-PD/not evaluated	No	PR	
SD	Non-CR/Non-PD/not evaluated	No	SD	Documented at least once ≥4 wks. from baseline**
PD	Any	Yes or No	PD	no prior SD, PR or CR
Any	PD***	Yes or No	PD	
Any	Any	Yes	PD	

* See RECIST 1.1 manuscript for further details on what is evidence of a new lesion.
** For this trial confirmation of response is recommended but not required.
*** In exceptional circumstances, unequivocal progression in non-target lesions may be accepted as disease progression.

Note: Patients with a global deterioration of health status requiring discontinuation of treatment without objective evidence of disease progression at that time should be reported as "*symptomatic deterioration.*" Every effort should be made to document the objective progression even after discontinuation of treatment.

11.1.5 Duration of Response

Duration of overall response: The duration of overall response is measured from the time measurement criteria are met for CR or PR (whichever is first recorded) until the first date that recurrent or progressive disease is objectively documented (taking as reference for progressive disease the smallest measurements recorded since the treatment started).

The duration of overall CR is measured from the time measurement criteria are first met for CR until the first date that progressive disease is objectively documented.

Duration of stable disease: Stable disease is measured from the start of the treatment until the criteria for progression are met, taking as reference the smallest measurements recorded since the treatment started, including the baseline measurements.

11.1.6 Response Review

All objective responses will be reviewed by expert radiologist independent of study at the time of study completion.

11.2 Other Response Parameters

The clinical benefit rate will be defined as the proportion of patients with ≥ stable disease as best response lasting for > 6 months duration.

12. INFORMED CONSENT, STUDY OVERSIGHT AND DATA REPORTING / REGULATORY REQUIREMENTS

Adverse event lists, guidelines, and instructions for AE reporting can be found in Section 7.0 (Adverse Events: List and Reporting Requirements).

12.1 Informed Consent

12.1.1 Waiver of Consent for Recruitment Purposes
This study is requesting a waiver of consent to access health records and NGS panel databases (e.g. UCSF 500, Foundation One, Oncopanel, Oncomine, Tempus xT) during the recruitment process to identify eligible patients. It is not practicable to obtain informed consent from patients for access to this information until they have been identified. A waiver for screening of health records and NGS panel databases poses no more than minimal risk and will not adversely affect the rights and welfare of the patients. The study team will not directly contact identified patients who are not under the care of a study investigator; the study team will contact the provider(s) of these identified eligible patient(s) to inform them of the study and ask the providers to consider discussing the study with the identified patient(s).

12.1.2 Informed Consent for Study Participation
All study participants will be provided a consent form describing the study with sufficient information for each participant to make an informed decision regarding their participation in the trial. Participants must sign the IRB -approved informed consent form prior to participation in any study specific procedure.

12.2 Study Oversight

This protocol is monitored at several levels, as described in this section. The Protocol Principal Investigator is responsible for monitoring the conduct and progress of the clinical trial, including the ongoing review of accrual, patient-specific clinical and laboratory data, and routine and

serious adverse events; reporting of expedited adverse events; and accumulation of reported adverse events from other trials testing the same drug(s). The Protocol Principal Investigator and statistician have access to the data at all times through the CTMS web-based reporting portal.

The Protocol Principal Investigator will have, at a minimum, quarterly conference calls with the Study Investigators and the CTEP Medical Officer(s) to review accrual, progress, and pharmacovigilance. Decisions to proceed to the second stage of a Phase 2 trial will require sign-off by the Protocol Principal Investigator and the Protocol Statistician.

All Study Investigators at participating sites who register/enroll patients on a given protocol are responsible for timely submission of data via Medidata Rave and timely reporting of adverse events for that particular study. This includes timely review of data collected on the electronic CRFs submitted via Medidata Rave.

All studies are also reviewed in accordance with the enrolling institution's data safety monitoring plan.

12.3 Data Reporting

Data collection for this study will be done exclusively through Medidata Rave. Access to the trial in Rave is granted through the iMedidata application to all persons with the appropriate roles assigned in the Regulatory Support System (RSS). To access Rave via iMedidata, the site user must have an active CTEP IAM account (check at < https://ctepcore.nci.nih.gov/iam/index.jsp>) and the appropriate Rave role (Rave CRA, Read-Only, CRA (Lab Admin, SLA or Site Investigator) on either the LPO or participating organization roster at the enrolling site. To the hold Rave CRA role or CRA Lab Admin role, the user must hold a minimum of an AP registration type. To hold the Rave Site Investigator role, the individual must be registered as an NPIVR or IVR. Associates can hold read-only roles in Rave. If the study has a DTL, individuals requiring write access to Rave must also be assigned the appropriate Rave tasks on the DTL.

Upon initial site registration approval for the study in RSS, all persons with Rave roles assigned on the appropriate roster will be sent a study invitation e-mail from iMedidata. To accept the invitation, site users must log into the Select Login (https://login.imedidata.com/selectlogin) using their CTEP-IAM user name and password, and click on the "accept" link in the upper right-corner of the iMedidata page. Please note, site users will not be able to access the study in Rave until all required Medidata and study specific trainings are completed. Trainings will be in the form of electronic learnings (eLearnings), and can be accessed by clicking on the link in the upper right pane of the iMedidata screen.

Users that have not previously activated their iMedidata/Rave account at the time of initial site registration approval for the study in RSS will also receive a separate invitation from iMedidata to activate their account. Account activation instructions are located on the CTSU website, Rave tab under the Rave resource materials (Medidata Account Activation and Study Invitation Acceptance). Additional information on iMedidata/Rave is available on the CTSU members' website under the Rave tab or by contacting the CTSU Help Desk at 1-888-823-5923 or by e-mail at ctsucontact@westat.com.

12.3.1 Method

This study will be monitored by the Clinical Trials Monitoring Service (CTMS). Data will be submitted to CTMS at least once every two weeks via Medidata Rave (or other modality if approved by CTEP). Information on CTMS reporting is available at: http://www.theradex.com/clinicalTechnologies/?National-Cancer-Institute-NCI-11. On-site audits will be conducted on an 18-36 month basis as part of routine cancer center site visits. More frequent audits may be conducted if warranted by accrual or due to concerns regarding data quality or timely submission. For CTMS monitored studies, after users have activated their accounts, please contact the Theradex Help Desk at (609) 799-7580 or by email at CTMSSupport@theradex.com for additional support with Rave and completion of CRFs.

12.3.2 Responsibility for Data Submission

For ETCTN trials, it is the responsibility of the PI(s) at the site to ensure that all investigators at the ETCTN Sites understand the procedures for data submission for each ETCTN protocol and that protocol specified data are submitted accurately and in a timely manner to the CTMS via the electronic data capture system, Medidata Rave.

Data are to be submitted via Medidata Rave to CTMS on a real-time basis, but no less than once every 2 weeks. The timeliness of data submissions and timeliness in resolving data queries will be tracked by CTMS. Metrics for timeliness will be followed and assessed on a quarterly basis. For the purpose of Institutional Performance Monitoring, data will be considered delinquent if it is greater than 4 weeks past due.

Data from Medidata Rave and CTEP-AERS is reviewed by the CTMS on an ongoing basis as data is received. Queries will be issued by CTMS directly within Rave. The queries will appear on the Task Summary Tab within Rave for the CRA at the ETCTN to resolve. Monthly web-based reports are posted for review by the Drug Monitors in the IDB, CTEP. Onsite audits will be conducted by the CTMS to ensure compliance with regulatory requirements, GCP, and NCI policies and procedures with the overarching goal of ensuring the integrity of data generated from NCI-sponsored clinical trials, as described in the ETCTN Program Guidelines, which may be found on the CTEP (http://ctep.cancer.gov/protocolDevelopment/electronic_applications/adverse_events.htm) and CTSU websites.

An End of Study CRF is to be completed by the PI, and is to include a summary of study endpoints not otherwise captured in the database, such as (for phase 1 trials) the recommended phase 2 dose (RP2D) and a description of any dose-limiting toxicities (DLTs). CTMS will utilize a core set of eCRFs that are Cancer Data Standards Registry and Repository (caDSR) compliant (http://cbiit.nci.nih.gov/ncip/biomedical-informatics-resources/interoperability-and-semantics/metadata-and-models). Customized eCRFs will be included when appropriate to meet unique study requirements. The PI is encouraged to review the eCRFs, working closely with CTMS to ensure prospectively that all required items are appropriately captured in the eCRFs prior to study activation. CTMS will prepare the eCRFs with built-in edit checks to the extent

possible to promote data integrity.

CDUS data submissions for ETCTN trials activated after March 1, 2014, will be carried out by the CTMS contractor, Theradex. CDUS submissions are performed by Theradex on a monthly basis. The trial's lead institution is responsible for timely submission to CTMS via Rave, as above.

Further information on data submission procedures can be found in the ETCTN Program Guidelines (http://ctep.cancer.gov/protocolDevelopment/electronic_applications/adverse_events.htm).

12.4 Collaborative Agreements Language

The agent(s) supplied by CTEP, DCTD, NCI used in this protocol is/are provided to the NCI under a Collaborative Agreement (CRADA, CTA, CSA) between the Pharmaceutical Company(ies) (hereinafter referred to as "Collaborator(s)") and the NCI Division of Cancer Treatment and Diagnosis. Therefore, the following obligations/guidelines, in addition to the provisions in the "Intellectual Property Option to Collaborator" (http://ctep.cancer.gov/industryCollaborations2/intellectual_property.htm) contained within the terms of award, apply to the use of the Agent(s) in this study:

1. Agent(s) may not be used for any purpose outside the scope of this protocol, nor can Agent(s) be transferred or licensed to any party not participating in the clinical study. Collaborator(s) data for Agent(s) are confidential and proprietary to Collaborator(s) and shall be maintained as such by the investigators. The protocol documents for studies utilizing Agents contain confidential information and should not be shared or distributed without the permission of the NCI. If a copy of this protocol is requested by a patient or patient's family member participating on the study, the individual should sign a confidentiality agreement. A suitable model agreement can be downloaded from: http://ctep.cancer.gov.

2. For a clinical protocol where there is an investigational Agent used in combination with (an)other Agent(s), each the subject of different Collaborative Agreements, the access to and use of data by each Collaborator shall be as follows (data pertaining to such combination use shall hereinafter be referred to as "Multi-Party Data"):

   a. NCI will provide all Collaborators with prior written notice regarding the existence and nature of any agreements governing their collaboration with NCI, the design of the proposed combination protocol, and the existence of any obligations that would tend to restrict NCI's participation in the proposed combination protocol.

   b. Each Collaborator shall agree to permit use of the Multi-Party Data from the clinical trial by any other Collaborator solely to the extent necessary to allow said other Collaborator to develop, obtain regulatory approval or commercialize its own Agent.

   c. Any Collaborator having the right to use the Multi-Party Data from these trials must agree

in writing prior to the commencement of the trials that it will use the Multi-Party Data solely for development, regulatory approval, and commercialization of its own Agent.

3. Clinical Trial Data and Results and Raw Data developed under a Collaborative Agreement will be made available to Collaborator(s), the NCI, and the FDA, as appropriate and unless additional disclosure is required by law or court order as described in the IP Option to Collaborator (http://ctep.cancer.gov/industryCollaborations2/intellectual_property.htm). Additionally, all Clinical Data and Results and Raw Data will be collected, used and disclosed consistent with all applicable federal statutes and regulations for the protection of human subjects, including, if applicable, the *Standards for Privacy of Individually Identifiable Health Information* set forth in 45 C.F.R. Part 164.

4. When a Collaborator wishes to initiate a data request, the request should first be sent to the NCI, who will then notify the appropriate investigators (Group Chair for Cooperative Group studies, or PI for other studies) of Collaborator's wish to contact them.

5. Any data provided to Collaborator(s) for Phase 3 studies must be in accordance with the guidelines and policies of the responsible Data Monitoring Committee (DMC), if there is a DMC for this clinical trial.

6. Any manuscripts reporting the results of this clinical trial must be provided to CTEP by the Group office for Cooperative Group studies or by the principal investigator for non-Cooperative Group studies for immediate delivery to Collaborator(s) for advisory review and comment prior to submission for publication. Collaborator(s) will have 30 days from the date of receipt for review. Collaborator shall have the right to request that publication be delayed for up to an additional 30 days in order to ensure that Collaborator's confidential and proprietary data, in addition to Collaborator(s)'s intellectual property rights, are protected. Copies of abstracts must be provided to CTEP for forwarding to Collaborator(s) for courtesy review as soon as possible and preferably at least three (3) days prior to submission, but in any case, prior to presentation at the meeting or publication in the proceedings. Press releases and other media presentations must also be forwarded to CTEP prior to release. Copies of any manuscript, abstract and/or press release/ media presentation should be sent to:

Email: ncicteppubs@mail.nih.gov

The Regulatory Affairs Branch will then distribute them to Collaborator(s). No publication, manuscript or other form of public disclosure shall contain any of Collaborator's confidential/ proprietary information.

13. STATISTICAL CONSIDERATIONS

13.1 Study Design/Endpoints

The trial design is a Phase 2, single arm, Simon two-stage study evaluating two cohorts of patients, both with tumors with evidence of mutations in the *SETD2* gene: cohort A) advanced

solid tumor malignancies other than clear cell renal cell carcinoma, and B) clear cell renal cell carcinoma. The primary endpoint of the study for each patient cohort is the objective response rate by RECIST 1.1 criteria. Our null hypothesis is 5%. The alternative hypothesis is an objective response rate of 25%, based on the strong pre-clinical evidence of synthetic lethality of WEE1 inhibition in selected population of *SETD2*-deficient tumors, as well as the observed response rate with other approved therapies in treatment-refractory RCC (e.g. cabozantinib, nivolumab). An observed response rate of this magnitude, particularly if the responses are durable in nature, would provide a strong justification for a follow-on larger single arm or randomized study with registrational intent in *SETD2*-deficient RCC and potentially other tumor types.

Accrual will follow a Simon two-stage optimal design. 9 patients will be accrued in the first stage of the study in each of the two cohorts outlined above. In either cohort, if 1 or more confirmed objective responses are observed, an additional 21 patients will be accrued during Stage 2 in that particular patient cohort. If more than 4 confirmed objective responses are observed in total, the null hypothesis will be rejected for that patient cohort. The proposed sample size and accrual design achieves 90% power to detect a difference in objective response of 20% (25% vs. 5%) with a one-sided type I error rate of 5%.

Accrual will pause after 9 patients are enrolled in each cohort, until their responses have been determined by each local institution and overall response rate has been assessed by the Principal Investigator.

13.2 Sample Size/Accrual Rate

The planned sample size is approximately 18-60 patients. The anticipated accrual rate, based upon prior enrollment to studies involving renal cell, pancreatic NET tumors, as well as the expected frequency of SETD2 mutations in these two tumor types, is approximately 1.67 patients/month across all investigational sites.

PLANNED ENROLLMENT REPORT

Racial Categories	Ethnic Categories				Total
	Not Hispanic or Latino		Hispanic or Latino		
	Female	Male	Female	Male	
American Indian/ Alaska Native	0	0	0	0	**0**
Asian	3	2	0	0	**5**
Native Hawaiian or Other Pacific Islander	0	1	0	0	**1**
Black or African American	2	2	0	0	**4**

Racial Categories	Ethnic Categories				Total
	Not Hispanic or Latino		Hispanic or Latino		
	Female	Male	Female	Male	
White	7	7	2	2	18
More Than One Race	1	1	0	0	2
Total	13	13	2	2	30

PHS 398 / PHS 2590 (Rev. 08/12 Approved Through 8/31/2015) OMB No. 0925-0001/0002

13.3 Stratification Factors

There are no planned stratification factors.

13.4 Analysis of Secondary Endpoints

Clinical benefit is defined as patients who experience either objective response or stable disease for ≥ 6 months from start of study treatment. The clinical benefit rate across the entire study cohort will be descriptively reported along with 95% confidence interval.

The median duration of response will be descriptively reported using Kaplan-Meier product limit method along with 95% confidence interval.

The incidence and severity of adverse events, as graded by Common Toxicity Criteria Version 5.0, will be descriptively reported for the safety-evaluable population, defined as all patients who receive at least one dose of AZD1775.

For analysis of H3K36me3 mark by IHC with clinical outcomes, it is anticipated that approximately 67% of patients will have evaluable archival tumor tissue for this analysis. The study cohort will be dichotomized into those with low/absent H3K36me3 mark (0/1+) versus those with moderate/high nuclear mark (2+/3+). The objective response rate between cohorts will be compared using the chi-squared test. The median progression-free survival for each cohort will be determined using the Kaplan-Meier product limit method and compared between cohorts using the log-rank test.

Based on preliminary studies evaluating the IHC assay in tissue microarrays, it is anticipated that at least 80% of evaluable patients enrolled on the study will have low/absent H3K36me3 mark as only those with evidence of pathogenic loss of *SETD2* detected in the tumor using next generation sequencing panel will be enrolled. Therefore, the sample size will be limited to definitively evaluate for differences in outcomes based on H3K36me3 mark. Nevertheless, trends indicating enrichment of response and prolonged PFS in the low/absent mark subgroup, along with

confirmation of strong correlation between NGS sequencing results and absent H3K36me3 mark, would provide a strong justification to further develop this IHC test as a predictive biomarker of WEE1 inhibition.

13.5 Reporting and Exclusions

13.5.1 Evaluation of Toxicity

All patients will be evaluable for toxicity from the time of their first treatment with AZD1775.

13.5.2 Evaluation of Response

All patients included in the study must be assessed for response to treatment, even if there are major protocol treatment deviations or if they are ineligible. Each patient will be assigned one of the following categories: 1) complete response, 2) partial response, 3) stable disease, 4) progressive disease, 5) early death from malignant disease, 6) early death from toxicity, 7) early death because of other cause, or 8) unknown (not assessable, insufficient data).

All of the patients who met the eligibility criteria and receive at least one dose of AZD1775 should be included in the main analysis of the response rate. Patients in response categories 4-8 should be considered to have a treatment failure (disease progression). Thus, an incorrect treatment schedule or drug administration does not result in exclusion from the analysis of the response rate. Precise definitions for categories 4-8 will be protocol specific.

All conclusions should be based on all eligible patients. Subanalyses may then be performed on the basis of a subset of patients, excluding those for whom major protocol deviations have been identified (*e.g.*, early death due to other reasons, early discontinuation of treatment, major protocol violations, etc.). However, these subanalyses may not serve as the basis for drawing conclusions concerning treatment efficacy, and the reasons for excluding patients from the analysis should be clearly reported. The 95% confidence intervals should also be provided.

13.6 Interim Safety Analysis

An interim safety analysis will be conducted after the first 9 patients are enrolled in each cohort. If more than 33% of patients are required per protocol to discontinue treatment due to treatment-related adverse events during the first two cycles of study treatment, study accrual will be temporarily halted and alternative dosing schedules/starting doses may be considered. Accrual may continue during the analysis of interim safety.

REFERENCES

Al Sarakbi, W., Sasi, W., Jiang, W. G., Roberts, T., Newbold, R. F., and Mokbel, K. (2009). The mRNA expression of SETD2 in human breast cancer: correlation with clinico-pathological parameters. BMC Cancer *9*, 290.

Aymard, F., Bugler, B., Schmidt, C. K., Guillou, E., Caron, P., Briois, S., Iacovoni, J. S., Daburon, V., Miller, K. M., Jackson, S. P., and Legube, G. (2014). Transcriptionally active chromatin recruits homologous recombination at DNA double-strand breaks. Nat Struct Mol Biol *21*, 366-374.

Bridges, K. A., Hirai, H., Buser, C. A., Brooks, C., Liu, H., Buchholz, T. A., Molkentine, J. M., Mason, K. A., and Meyn, R. E. (2011). MK-1775, a novel Wee1 kinase inhibitor, radiosensitizes p53-defective human tumor cells. Clin Cancer Res *17*, 5638-5648.

Carvalho, S., Raposo, A. C., Martins, F. B., Grosso, A. R., Sridhara, S. C., Rino, J., Carmo-Fonseca, M., and de Almeida, S. F. (2013). Histone methyltransferase SETD2 coordinates FACT recruitment with nucleosome dynamics during transcription. Nucleic Acids Res *41*, 2881-2893.

Carvalho, S., Vítor, A. C., Sridhara, S. C., Martins, F. B., Raposo, A. C., Desterro, J. M., Ferreira, J., and de Almeida, S. F. (2014). SETD2 is required for DNA double-strand break repair and activation of the p53-mediated checkpoint. Elife *3*, e02482.

Coleman, T.R., Dunphy, W.G. (1994). Cdc2 regulatory factors. Current Opinion in Cell Biology *6*, 877-82.

Do, K., Wilsker, D., Ji, J., Zlott, J., Freshwater, T., Kinders, R.J., Collins, J., Chen, A.P., Doroshow, J.H., Kummar, S. (2015). Phase 1 study of single-agent AZD1775 (MK-1775), a Wee1 kinase inhibitor, in patients with refractory solid tumors. Journal of Clinical Oncology *33*, 3409-15.

Eisenhauer, E., Therasse, P., Bogaerts, J., Schwartz, L.H., Sargent, D., Ford, R., Dancey, J., Arbuck, S., Gwyther, S., Mooney, M. and Rubinstein, L. (2009). New response evaluation criteria in solid tumours: revised RECIST guideline (version 1.1). European journal of cancer, *45*(2), 228-247.

Guertin, A. D., Li, J., Liu, Y., Hurd, M. S., Schuller, A. G., Long, B., Hirsch, H. A., Feldman, I., Benita, Y., Toniatti, C., *et al.* (2013). Preclinical evaluation of the WEE1 inhibitor MK-1775 as single-agent anticancer therapy. Mol Cancer Ther *12*, 1442-1452.

Hakimi, A. A., Ostrovnaya, I., Reva, B., Schultz, N., Chen, Y. B., Gonen, M., Liu, H., Takeda, S., Voss, M. H., Tickoo, S. K., *et al.* (2013). Adverse outcomes in clear cell renal cell carcinoma with mutations of 3p21 epigenetic regulators BAP1 and SETD2: a report by MSKCC and the KIRC TCGA research network. Clin Cancer Res *19*, 3259-3267.

Parker, L. L., and Piwnica-Worms, H. (1992). Inactivation of the p34cdc2-cyclin B complex by the human WEE1 tyrosine kinase. Science *257*, 1955-1957.

Pfister, S. X., Ahrabi, S., Zalmas, L. P., Sarkar, S., Aymard, F., Bachrati, C. Z., Helleday, T., Legube, G., La Thangue, N. B., Porter, A. C., and Humphrey, T. C. (2014). SETD2-dependent histone H3K36 trimethylation is required for homologous recombination repair and genome stability. Cell Rep *7*, 2006-2018.

Pfister, S. X., Markkanen, E., Jiang, Y., Sarkar, S., Woodcock, M., Orlando, G., Mavrommati, I., Pai, C. C., Zalmas, L. P., Drobnitzky, N., *et al.* (2015). Inhibiting WEE1 Selectively Kills Histone H3K36me3-Deficient Cancers by dNTP Starvation. Cancer Cell *28*, 557-568.

Rajeshkumar, N. V., De Oliveira, E., Ottenhof, N., Watters, J., Brooks, D., Demuth, T., Shumway, S. D., Mizuarai, S., Hirai, H., Maitra, A., and Hidalgo, M. (2011). MK-1775, a potent Wee1 inhibitor, synergizes with gemcitabine to achieve tumor regressions, selectively in p53-deficient pancreatic cancer xenografts. Clin Cancer Res *17*, 2799-2806.

Sarcar, B., Kahali, S., Prabhu, A. H., Shumway, S. D., Xu, Y., Demuth, T., and Chinnaiyan, P. (2011). Targeting radiation-induced G(2) checkpoint activation with the Wee-1 inhibitor MK-1775 in glioblastoma cell lines. Mol Cancer Ther *10*, 2405-2414.

Sherr, C.J. (1996). Cancer cell cycles. Science *274*, 1672-77.

Tominaga, Y., Li, C., Wang, R. H., and Deng, C. X. (2006). Murine Wee1 plays a critical role in cell cycle regulation and pre-implantation stages of embryonic development. Int J Biol Sci *2*, 161-170.

Wang, Y., Li, J., Booher, R.N., Kraker, A., Lawrence, T., Leopold, W.R., Sun, Y. (2001). Radiosensitization of p53 mutant cells by PD0166285, a novel G2 checkpoint abrogator. Cancer Research *61*, 8211-17.

Watanabe, N., Broome, M., and Hunter, T. (1995). Regulation of the human WEE1Hu CDK tyrosine 15-kinase during the cell cycle. EMBO J *14*, 1878-1891.

Zhang, Y., Xie, S., Zhou, Y., Xie, Y., Liu, P., Sun, M., Xiao, H., Jin, Y., Sun, X., Chen, Z., *et al.* (2014). H3K36 histone methyltransferase Setd2 is required for murine embryonic stem cell differentiation toward endoderm. Cell Rep *8*, 1989-2002.

Zhu, X., He, F., Zeng, H., Ling, S., Chen, A., Wang, Y., Yan, X., Wei, W., Pang, Y., Cheng, H., *et al.* (2014). Identification of functional cooperative mutations of SETD2 in human acute leukemia. Nat Genet *46*, 287-293.

APPENDIX A PERFORMANCE STATUS CRITERIA

**ECOG Performance Status Scale**		**Karnofsky Performance Scale**	
Grade	Descriptions	Percent	Description
0	Normal activity. Fully active, able to carry on all pre-disease performance without restriction.	100	Normal, no complaints, no evidence of disease.
		90	Able to carry on normal activity; minor signs or symptoms of disease.
1	Symptoms, but ambulatory. Restricted in physically strenuous activity, but ambulatory and able to carry out work of a light or sedentary nature (*e.g.*, light housework, office work).	80	Normal activity with effort; some signs or symptoms of disease.
		70	Cares for self, unable to carry on normal activity or to do active work.
2	In bed <50% of the time. Ambulatory and capable of all self-care, but unable to carry out any work activities. Up and about more than 50% of waking hours.	60	Requires occasional assistance, but is able to care for most of his/her needs.
		50	Requires considerable assistance and frequent medical care.
3	In bed >50% of the time. Capable of only limited self-care, confined to bed or chair more than 50% of waking hours.	40	Disabled, requires special care and assistance.
		30	Severely disabled, hospitalization indicated. Death not imminent.
4	100% bedridden. Completely disabled. Cannot carry on any self-care. Totally confined to bed or chair.	20	Very sick, hospitalization indicated. Death not imminent.
		10	Moribund, fatal processes progressing rapidly.
5	Dead.	0	Dead.

APPENDIX B PATIENT DRUG INFORMATION HANDOUT AND WALLET CARD

Information for Patients, Their Caregivers and Non-Study Healthcare Team on Possible Interactions with Other Drugs and Herbal Supplements

The patient ____________________________ is enrolled on a clinical trial using the experimental study drug **AZD1775 (MK-1775)**. This clinical trial is sponsored by the National Cancer Institute. This form is addressed to the patient, but includes important information for others who care for this patient.

These are the things that you as a prescriber need to know:

AZD1775 (MK-1775) interacts with certain specific enzymes in the liver and certain transport proteins that help move drugs in and out of cells.

- The enzymes in question are ***CYP 3A4 and 2C19***. AZD1775 (MK-1775) is metabolized by CYP3A4 and may be affected by other drugs that inhibit or induce these enzymes. AZD1775 (MK-1775) is an inhibitor of CYP 3A4 and 2C19 and may affect the metabolism of other drugs.
- The proteins in question are ***OATP1B1, OATP1B3, MATE1, MATE2K, P-gp, and BCRP.*** AZD1775 (MK-1775) is a substrate of P-gp and BCRP and may be affected by other drugs that inhibit or induce these transporters. AZD1775 (MK-1775) is an inhibitor of OATP1B1, OATP1B3, MATE1, MATE2K, P-gp, and BCRP and may affect transport of other drugs in and out of cells.

To the patient: Take this paper with you to your medical appointments and keep the attached information card in your wallet.

AZD1775 (MK-1775) may interact with other drugs which can cause side effects. For this reason, it is very important to tell your study doctors of any medicines you are taking before you enroll onto this clinical trial. It is also very important to tell your doctors if you stop taking any regular medicines, or if you start taking a new medicine while you take part in this study. When you talk about your current medications with your doctors, include medicine you buy without a prescription (over-the-counter remedy), or herbal supplements such as St. John's Wort. It is helpful to bring your medication bottles or an updated medication list with you.

Many health care providers can write prescriptions. You must tell all of your health care providers (doctors, physician assistants, nurse practitioners, or pharmacists) you are taking part in a clinical trial.

These are the things that you and they need to know:

AZD1775 (MK-1775) must be used very carefully with other medicines that need certain **liver**

enzymes or transport proteins to be effective or to be cleared from your system. Before you enroll onto the clinical trial, your study doctor will work with your regular health care providers to review any medicines and herbal supplements that are considered "strong inducers/inhibitors or substrates of **CYP 3A4, 2C19, OATP1B1, OATP1B3, MATE1, MATE2K, P-gp, and BCRP."**

- Please be very careful! Over-the-counter drugs (including herbal supplements) may contain ingredients that could interact with your study drug. Speak to your doctors or pharmacist to determine if there could be any side effects.

- Your regular health care provider should check a frequently updated medical reference or call your study doctor before prescribing any new medicine or discontinuing any medicine. Your study doctor's name is

____________________________________ and he or she can be contacted at

____________________________________.

STUDY DRUG INFORMATION WALLET CARD	
You are enrolled on a clinical trial using the experimental study drug **AZD1775 (MK-1775).** This clinical trial is sponsored by the NCI. **AZD1775 (MK-1775)** may interact with drugs that are **processed by your liver, or use certain transport proteins in your body**. Because of this, it is very important to:<br>➢ Tell your doctors if you stop taking any medicines or if you start taking any new medicines.<br>➢ Tell all of your health care providers (doctors, physician assistants, nurse practitioners, or pharmacists) that you are taking part in a clinical trial.<br>➢ Check with your doctor or pharmacist whenever you need to use an over-the-counter medicine or herbal supplement.	**AZD1775 (MK-1775)** interacts with **CYP 3A4, 2C19, OATP1B1, OATP1B3, MATE1, MATE2K, P-gp, and BCRP**, and must be used very carefully with other medicines that interact with these enzymes and proteins.<br>➢ Before you enroll onto the clinical trial, your study doctor will work with your regular health care providers to review any medicines and herbal supplements that are considered **"strong inducers/inhibitors or substrates of CYP 3A4, 2C19, OATP1B1, OATP1B3, MATE1, MATE2K, P-gp, and BCRP"**<br>➢ Before prescribing new medicines, your regular prescribers should go to a frequently-updated medical reference for a list of drugs to avoid, or contact your study doctor.<br>➢ Your study doctor's name is ________________________<br>and can be contacted at .

APPENDIX C STUDY DRUG DIARY

- You will take AZD1775 by mouth once a day on days 1-5 and 8-12 for a total of ten doses every 21 days. The drug is given in cycles; each cycle is 21 days long.
- Take AZD1775 on an empty stomach, two hours before a meal
  OR two hours after a meal.
- Capsules should be swallowed whole.
- If you forget a dose, and if more than 12 hours past your scheduled time, the missed capsules should not be taken. If it is within 12 hours of the scheduled time, you should take the medication and resume regular dosing schedule the next day.
- Do not retake the dose if you vomit it. Wait until the next scheduled dose of AZD1775. If no dose is scheduled for the following day, the dose will not be 'made up'. If your vomiting continues, please contact the study doctor.
- Please bring the empty bottle or any leftover capsules and your pill calendar to your next clinic visit.

Patient Name: ______________________________ Cycle # ___________

Patient ID:_________________________ Prescribed Dose: _______ mg

Day of Treatment	Date (e.g. 06-Jan-2019)	Time (e.g. 14:35)	Missed Meds? (Yes or No)	Dose Taken (mg)
Day 1		:	☐ Yes ☐ No	
Day 2		:	☐ Yes ☐ No	
Day 3		:	☐ Yes ☐ No	
Day 4		:	☐ Yes ☐ No	
Day 5		:	☐ Yes ☐ No	
Day 6				
Day 7				
Day 8		:	☐ Yes ☐ No	
Day 9		:	☐ Yes ☐ No	
Day 10		:	☐ Yes ☐ No	
Day 11		:	☐ Yes ☐ No	
Day 12		:	☐ Yes ☐ No	
Day 13				
Day 14				
Day 15				
Day 16				
Day 17				
Day 18				
Day 19				
Day 20				
Day 21				

Please return the completed drug diary and unused drug and/or empty bottles to the study site during your next scheduled visit.

Patient Signature: ______________________________ Date: _______________________
