# Supplementary material for: A Phase II Trial of the WEE1 Inhibitor Adavosertib in SETD2-Altered Advanced Solid Tumor Malignancies (NCI 10170)
Source: Cancer Res Commun. 2024 Jul 23;4(7):1793–801. doi: 10.1158/2767-9764.CRC-24-0213 (PMC11264598; doi:10.1158/2767-9764.CRC-24-0213)

**Supplementary Figure S2.** Example of no loss of H3K36me3 by IHC (A) and the loss of H3K36me3 by IHC (B). Images are at 40x power. The image below is a TIFF format.


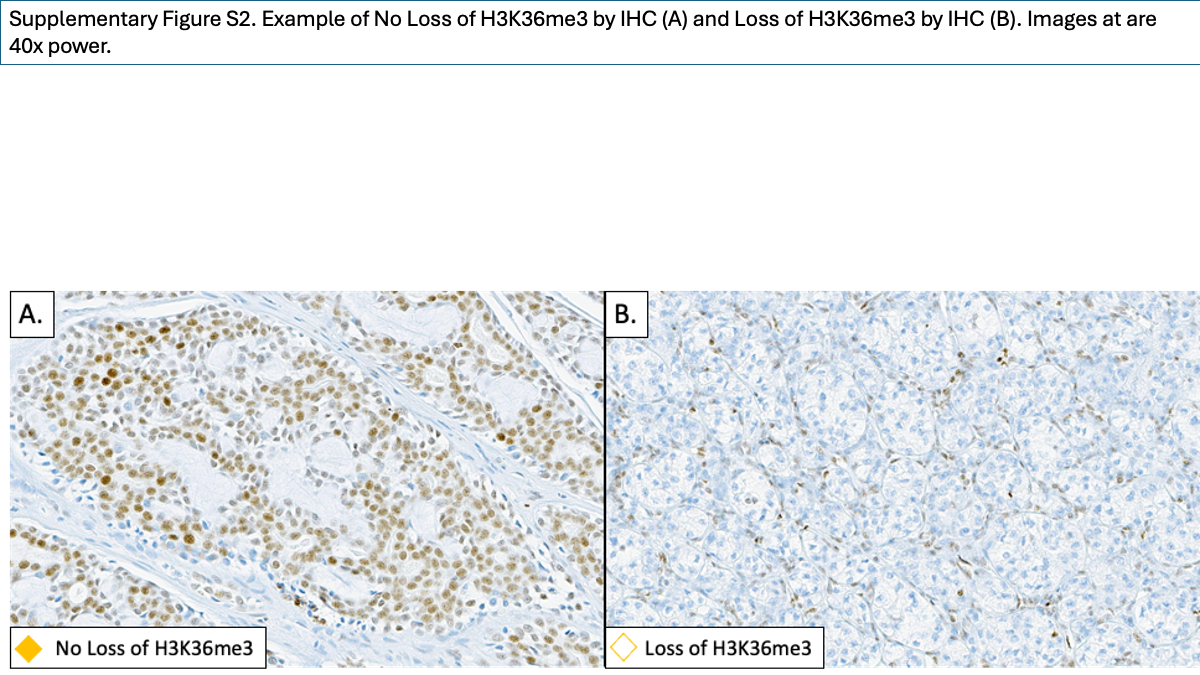

Supplement: Supplementary Figure S2 — shows an example of no loss of H3K36me3 by IHC (A) and loss of H3K36me3 by IHC (B). Images are at 40x power. Of note, the portal did not allow for uploading of a TIFF file alone as a "supplemental data" file, so the image included in this document is a TIFF file. We can easily provide the TIFF file if needed as well. [file crc-24-0213_supplementary_figure_s2_supps2.docx]
