# Supplementary material for: A Phase II Trial of the WEE1 Inhibitor Adavosertib in SETD2-Altered Advanced Solid Tumor Malignancies (NCI 10170)
Source: Cancer Res Commun. 2024 Jul 23;4(7):1793–801. doi: 10.1158/2767-9764.CRC-24-0213 (PMC11264598; doi:10.1158/2767-9764.CRC-24-0213)

**Supplementary Figure S3**. All 8 patients with H3K36me3 IHC analysis. (A) Cohort A: Other Solid Tumor. (B) Cohort B: ccRCC. Images are at 40x Power. The image below is a TIFF format.


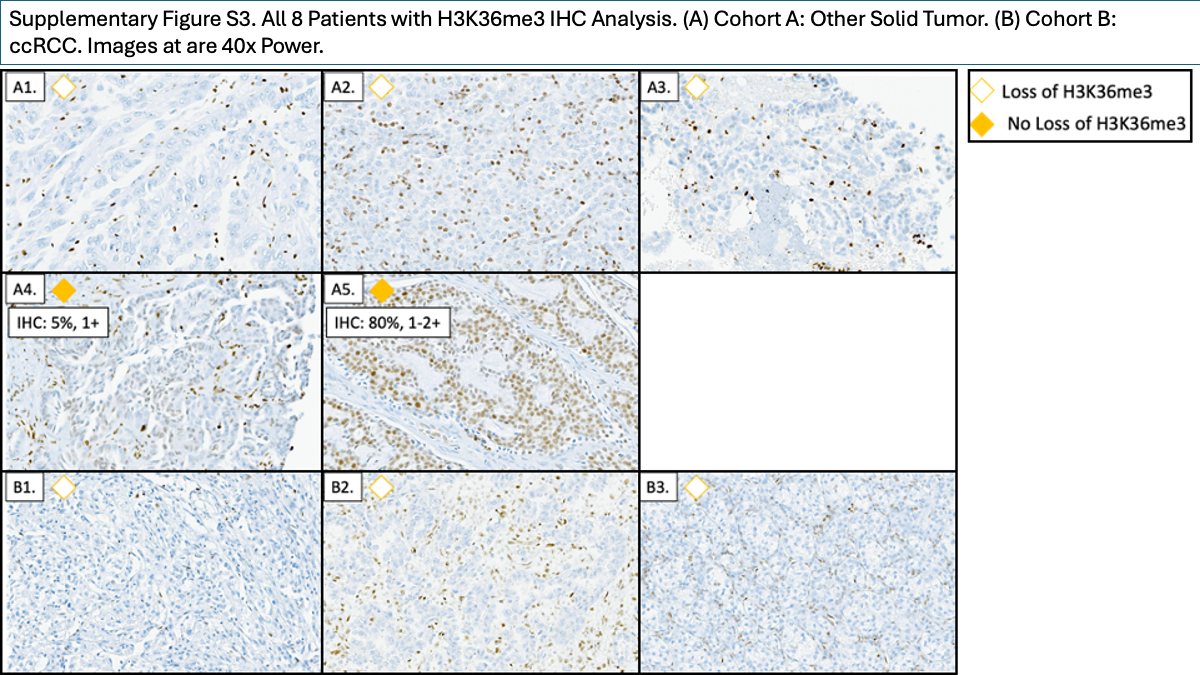

Supplement: Supplementary Figure S3 — shows all 8 patients with H3K36me3 IHC analysis. (A) Cohort A: Other Solid Tumor. (B) Cohort B: ccRCC. Images are at 40x power. Of note, the portal did not allow for uploading of a TIFF file alone as a "supplemental data" file, so the image included in this document is a TIFF file. We can easily provide the TIFF file if needed as well. [file crc-24-0213_supplementary_figure_s3_supps3.docx]
