# Supplementary material for: A Phase II Trial of the WEE1 Inhibitor Adavosertib in SETD2-Altered Advanced Solid Tumor Malignancies (NCI 10170)
Source: Cancer Res Commun. 2024 Jul 23;4(7):1793–801. doi: 10.1158/2767-9764.CRC-24-0213 (PMC11264598; doi:10.1158/2767-9764.CRC-24-0213)
Supplement: Supplementary Table S4 — is a table showing the representativeness of study participants. [file crc-24-0213_supplementary_table_s4_supps4.docx]

**Supplementary Table S4.** Representativeness of Study Participants.

| Cancer Type(s)/subtype(s)/stage(s)/condition | Clear Cell Renal Cell Carcinoma (ccRCC) in one of two cohorts. The second cohort consisted of various solid tumor malignancies including: poorly-differentiated carcinoma, salivary gland carcinoma, rectal adenocarcinoma, neuroendocrine carcinoma, pancreatic adenocarcinoma, lung adenocarcinoma, non-clear cell renal cell carcinoma. |
| --- | --- |
| Considerations related to: | |
| Sex | Per the American Cancer Society, new kidney cancer diagnoses in the United States occur in 5% of biological males and 3% of biological females (3%). Estimated deaths from kidney cancer occur in 3% of biological males and 2% of biological females. Incidence of pancreas cancer is similar between biological males (3%) and biological females (3%). Incidence of lung cancer is also similar between biological males (11%) and biological females (12%). |
| Age | Kidney cancer is often diagnosed at a median age of 64 years. Pancreatic cancer is often diagnosed at between age 60-80 years. Lung cancer age at diagnosis is variable pending smoking status, other environmental exposures, and mutation status. |
| Race/ethnicity | According to the American Cancer Society In the United States, stage distribution by race for kidney cancers were as follows: localized (67% all races, 74% black, 66% white); regional (15% all races, 10% black, 16% white); distant (13% all races, 11% black, 14% white); and unstaged (5% amongst all groups). The 5-year relative survival rates by race for kidney cancer were as follows: localized (93% all races, 91% black, 94% white); regional (74% all races, 65% black, 74% white); distant (17% all races, 13% black, 17% white); all stages (78% all races, 77% black, 78% white).  When the ACS evaluated incidence of kidney cancer by race, rates of kidney cancer incidence was higher in various subgroups when compared to non-Hispanic white patients (listed as 24.3/100,000 population in biological males and 12.1/100,000 population in biological females). The incidence rates (per 100,000 population) by race in biological males and females respectively are as follows: Black (26.4, 13.7); American Indian/Alaska Native (43.9/23.9); Asian American Pacific Islander (11.6/5.5); Hispanic/Latinx (23.5/13.3).  In another study evaluating age-adjusted incidence rates of kidney cancer, incidence by race (per 100,000 person years) was approximately 11.7 for non-Hispanic white; 12.7 of non-Hispanic African American; 5.9 of Non-Hispanic Asian Pacific Islander; 14.1 of non-Hispanic American Indian/Alaska Native; and 11.6 Hispanic. Of these, kidney cancer incidence was approximately two-fold higher in biological males when compared to females. In metastatic kidney cancer, studies have been mixed regarding outcome differences by race/ethnicity, though some retrospective analyses of large databases such as SEER have noticed differences in overall survival at 5 years when comparing to population-based controls, specifically in African Americans, followed by Hispanics, Caucasians, then Asian/Pacific Islanders. In these studies, African Americans exhibited the highest Hazard Ratio for death. |
| Geography | Kidney cancer deaths in the United states account for 9,450/322,800 biological males and 4,940/288,920 biological females. Our study enrolled patients on the East Coast, West Coast, Mid-West, and South). |
| Other Considerations: | A 2022 study previously evaluated National Cancer Institute (NCI)-sponsored therapeutic clinical trials between 2015-2019 and incidence data from the Centers for Disease Control and Prevention (CDC) between 2015-2017 for kidney cancer trials and race/ethnicity recruitment compared to non-Hispanic white patients. For kidney cancer, they found that Black patients (Odds Ratio 0.42, 95% confidence interval 0.33-0.54) and Hispanic patients (Odds ratio 0.68, 95% confidence interval 0.55-0.83) were under-represented in therapeutic clinical trials when compared to non-Hispanic white patients. Multivariable logistic regression analysis comparing the years 2000-2004 and 2015-2019 noted no change in the participation in kidney cancer trials of black patients (Odds ratio 1.17 p=0.293); increased participation in trials for Hispanic patients (Odds ratio 2.54, 95% confidence interval 1.88-3.43) and Asian/Pacific Islander patients (Odds ratio 2.27, 95% confidence interval 1.34-3.83).  Overall, underestimation of minorities and other underrepresented groups in clinical trials relative to cancer incidence within these patient populations further highlight the continued need for systemic changes to help improve minority/underrepresented person recruitment to clinical trials in the future. |
| Overall Representativeness of this Study | The age distribution of our study is relatively similar to the age of diagnosis in the literature.  Efforts were made to accrue patients from various geographic locations (West Coast, East Coast, mid-West, the South), in hopes this would help accrual of a diverse patient population for this small 18-patient phase 2 study. However, the patients accrued to the study were predominantly non-Hispanic white patients.  One of the two cohorts allowed patients with all types of solid tumor malignancies, though all patients were required to have a pathogenic *SETD2* alteration/mutation to be included in the study. |

References:

1. Siegel RL, Giaquinto AN, Jemal A. Cancer statistics, 2024. CA Cancer J Clin. 2024 Jan-Feb;74(1):12-49. doi: 10.3322/caac.21820. Epub 2024 Jan 17. Erratum in: CA Cancer J Clin. 2024 Feb 16; PMID: 38230766.
2. Palumbo C, Pecoraro A, Knipper S, Rosiello G, Luzzago S, Deuker M, Tian Z, Shariat SF, Simeone C, Briganti A, Saad F, Berruti A, Antonelli A, Karakiewicz PI. Contemporary Age-adjusted Incidence and Mortality Rates of Renal Cell Carcinoma: Analysis According to Gender, Race, Stage, Grade, and Histology. Eur Urol Focus. 2021 May;7(3):644-652. doi: 10.1016/j.euf.2020.05.003. Epub 2020 May 23. PMID: 32456993.
3. Jivanji D, Jamieson S, Mallory C, Wong V, Barrau S, Atri E, Castro G, Barengo NC, Nieder AM. The Association Between Race and 5-year Survival in Patients With Clear Cell Renal Cell Carcinoma: A Cohort Study. Urology. 2021 Feb;148:185-191. doi: 10.1016/j.urology.2020.10.055. Epub 2020 Dec 5. PMID: 33285213.
4. Javier-DesLoges J, Nelson TJ, Murphy JD, McKay RR, Stewart TF, Kader AK, Derweesh I, Martinez ME, Rose BS. An evaluation of trends in the representation of patients by age, sex, and diverse race/ethnic groups in bladder and kidney cancer clinical trials. Urol Oncol. 2022 May;40(5):199.e15-199.e21. doi: 10.1016/j.urolonc.2022.03.013. Epub 2022 Apr 14. PMID: 35431133; PMCID: PMC10441556.
